# Osteoblastic sclerostin loop3-LRP4 interaction required by sclerostin to inhibit bone formation

**DOI:** 10.1038/s41413-026-00511-x

**Published:** 2026-04-24

**Authors:** Luyao Wang, Xiaohui Tao, Hewen Jiang, Shijian Ding, Ning Zhang, Xin Yang, Shenghang Wang, Yihao Zhang, Nanxi Li, Haitian Li, Zhanghao Li, Xiaoxin Wen, Meiheng Sun, Chuanxin Zhong, Heiwa So, Jin Liu, Yuanyuan Yu, Hua Yue, Xianghang Luo, Péter Ferdinandy, Tao Zhang, Shu Zhang, Zhenlin Zhang, Aiping Lu, Baoting Zhang, Ge Zhang

**Affiliations:** 1 Law Sau Fai Institute for Advancing Translational Medicine in Bone and Joint Diseases (TMBJ), Hong Kong Baptist University, Hong Kong SAR, China; 2 Guangdong-Hong Kong-Macao Greater Bay Area International Research Platform for Aptamer-based Translational Medicine and Drug Discovery (HKAP), Hong Kong SAR, China; 3 Institute of Integrated Bioinformedicine and Translational Science (IBTS), Hong Kong Baptist University, Hong Kong SAR, China; 4 School of Chinese Medicine, Faculty of Medicine, The Chinese University of Hong Kong, Hong Kong SAR, China; 5 Department of Osteoporosis and Bone Diseases, Shanghai Clinical Research Center of Bone Diseases, Shanghai Sixth People’s Hospital Affiliated to Shanghai Jiao Tong University School of Medicine, Shanghai, China; 6 Xiangya Hospital of Central South University, Hu Nan, China; 7 Department of Pharmacology and Pharmacotherapy, Semmelweis University, Budapest, Hungary; 8 Shanghai Institute of Technical Physics Chinese Academy of Sciences, Shanghai, China; 9 The Key Laboratory of Aerospace Medicine, Ministry of Education, Air Force Medical University, Xi’an, Shanxi China

**Keywords:** Pathogenesis, Metabolic bone disease, Bone

## Abstract

Sclerostin negatively regulates bone formation. The marketed antibody against sclerostin loop2 promoted bone formation but may have caused severe cardiovascular events in clinical use. In our published studies, sclerostin loop3 was found to be involved in inhibitory effects of sclerostin on bone formation, whereas cardiovascular protective effects of sclerostin in mice were independent of loop3. It is necessary to investigate how sclerostin loop3 participates in the inhibitory effects of sclerostin on bone formation to facilitate developing precise strategies that promote bone formation without increasing cardiovascular risk. In this study, sclerostin loop3 was identified to bind to LRP4, thereby facilitating binding of sclerostin to LRP6 in osteoblasts. Blockade of sclerostin loop3-LRP4 interaction by both *Lrp4* mutation (*Lrp4m*) and blocking peptide (LRP4-Pep) diminished the antagonistic effect of sclerostin on Wnt/β-catenin signaling in osteoblasts in vitro. Consistently, *Lrp4m* promoted bone formation in *Lrp4m* mice in vivo. Mechanistically, osteoblast-conditional correction of *Lrp4m* to wild-type *Lrp4* resulted in significantly lower bone formation than *Lrp4m* mice, indicating that the promotive effects of *Lrp4m* on bone formation acted in osteoblasts in vivo. Moreover, re-expression of sclerostin dramatically inhibited bone formation in *sost*^*−/−*^ mice, whilst the inhibitory effects of sclerostin were significantly weaker in *sost*^*−/−*^*.Lrp4m* mice. Pharmacologically, LRP4-Pep diminished the inhibitory effects of sclerostin on bone formation in *SOST*^*ki*^ mice. Taken together, osteoblastic sclerostin loop3-LRP4 interaction, as an anchor, was required by sclerostin to bind to LRP6, thereby inhibiting bone formation. Translationally, blockade of sclerostin loop3-LRP4 interaction in osteoblasts would provide precise therapeutic strategies to promote bone formation without increasing cardiovascular risk.

## Introduction

Sclerostin, which negatively regulates bone formation, has become a novel bone anabolic target. There are three loops within its central region, including loop1, loop2, and loop3.^1^ The marketed therapeutic antibody mainly targeting sclerostin loop2 was demonstrated to promote bone formation for postmenopausal osteoporosis, whereas it may have caused severe cardiovascular events in clinical trials (BRIDGE and ARCH).^2–7^ US-FDA (Food and Drug Administration) gives a black-boxed warning on the risk of heart attack, stroke and cardiovascular death (FDA Press Announcements). EMA (European Medicines Agency) stated that it must not be used to patients who had diseases history of heart attack and stroke (European Medicines Agency Documents). In one nonclinical study, the therapeutic sclerostin antibody was reported to have no effect on the total atherosclerosis plaque volume in *ApoE*^*−/−*^ mice with AngII infusion.^8^ In another nonclinical study reported by US-FDA, the therapeutic sclerostin antibody increased the incidence of plaques with necrosis of all types (2–5) and upregulated the local expression of inflammatory cytokines and chemokines in ovariectomized (OVX) *ApoE*^*−/−*^ mice with high-fat diet. Despite continuing controversy, cardiovascular risk of sclerostin inhibition cannot be definitively ruled out. In our published work, it was notably found that sclerostin loop3 contributed to the inhibitory effect of sclerostin on bone formation, while the inhibitory effects of sclerostin on inflammation, atherosclerosis and aortic aneurysm in *ApoE*^*−/−*^ mice were independent of sclerostin loop3.^9^ Sclerostin loop3 emerges as a precise therapeutic target for promoting bone formation, without cardiovascular concerns.^9–11^ Nevertheless, how sclerostin loop3 participates in the inhibitory effect of sclerostin on bone formation remains unclear, which is a hurdle to develop the precise bone anabolic strategy without cardiovascular risk.

Sclerostin was known to antagonize bone anabolic Wnt/β-catenin signaling pathway in osteoblasts through the interaction between sclerostin loop2 and low-density lipoprotein receptor-related protein 5/6 (LRP5/6).^12^ Clinically, relevant variants affecting LRP5 or LRP6 were detected with low-bone-mass disorders, like osteoporosis pseudoglioma syndrome.^13,14^ Low-density lipoprotein receptor-related protein 4 (LRP4) is another member of the LDL receptor family. Interestingly, *Lrp4* mutations were identified in patients with high-bone-mass disorders, such as sclerosteosis and van Buchem diseases.^15^ LRP4-null mutation or osteoblastic mutations increased trabecular and cortical bone mass, which was associated with elevated bone formation and impaired bone resorption.^15^ It was reported that osteoblastic LRP4 facilitated the binding of sclerostin to LRP6 for antagonizing bone formation.^16,17^

In this study, we identified LRP4 as the receptor of sclerostin loop3 in osteoblasts, followed by identification of their interaction residues within LRP4 to develop genetic blocking approach (*Lrp4m*) and pharmacologic blocking approach (LRP4-Pep) for the following structure-function studies. Either *Lrp4m* or LRP4-Pep was employed to block sclerostin loop3-LRP4 interaction for determining whether sclerostin loop3-LRP4 interaction was required by sclerostin to bind to LRP6 and to antagonize Wnt/β-catenin signaling and osteogenic potential in osteoblasts in vitro. Then, the promotive effect of *Lrp4m* on bone formation was validated in vivo. Thereafter, to determine whether the promotive effect of *Lrp4m* on bone formation acted in osteoblasts, osteoblastic *Lrp4m* was conditionally corrected to wild-type *Lrp4* in *Lrp4m/OB-Lrp4* mice. Subsequently, bone formation, bone mass, bone microarchitecture and bone mechanical properties were determined and compared between *Lrp4m/OB-Lrp4* mice and *Lrp4m* mice. Moreover, *sost*^*−/−*^*.Lrp4m* mouse model was established to shield the effect of endogenous sclerostin. Genetically, the role of sclerostin loop3-LRP4 interaction in the inhibitory effect of sclerostin on bone formation in vivo was determined by comparing the bone formation between *sost*^*−/−*^*.Lrp4m* mice and *sost*^*−/−*^ mice, with and without AAV-induced re-expression of sclerostin. Pharmacologically, the role of sclerostin loop3-LRP4 interaction in the inhibitory effect of sclerostin on bone formation in vivo was determined by comparing the bone formation in *SOST*^*ki*^ mice between with and without treatment of LRP4-Pep. Bridging molecular insight to therapeutic application, specific blockade of sclerostin loop3-LRP4 interaction in osteoblasts would provide precise bone anabolic strategy without cardiovascular risk.

## Results

### Sclerostin loop3 bound to LRP4, rather than LRP6, in osteoblasts

**Fig. 1:**
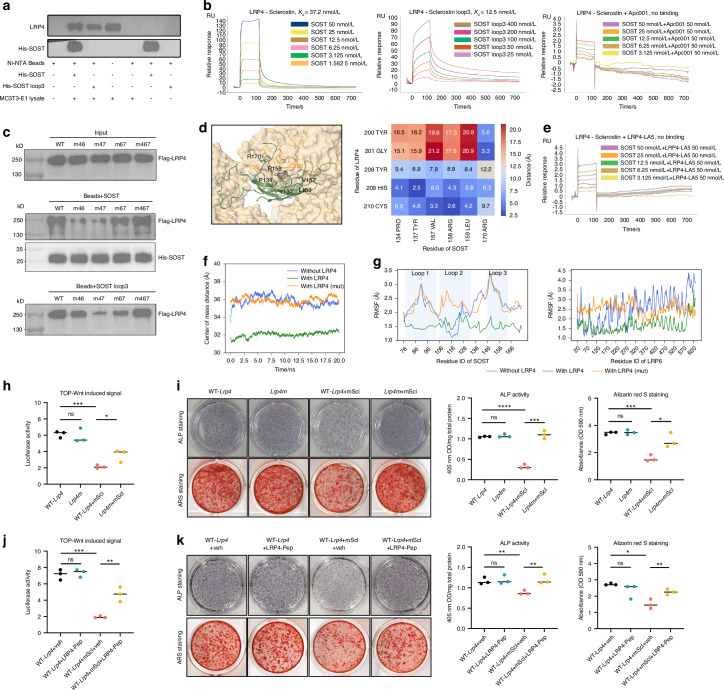
Blockade of sclerostin loop3-LRP4 interaction diminished the antagonistic effects of sclerostin on Wnt/β-catenin signaling and osteogenic potential in osteoblasts in vitro. **a** Binding analysis for the interaction of LRP4 to full-length sclerostin (FL SOST) and sclerostin loop3 (SOST loop3) in osteoblasts (MC3T3-E1 cells) by pull-down assay. **b** The binding affinity of LRP4 to sclerostin loop3 (the middle panel), and the binding affinity of LRP4 to sclerostin with (the left panel) and without (the right panel) the pretreatment of validated sclerostin loop3-specific aptamer Apc001, respectively, by SPR analysis. **c** The binding ability of LRP4 muteins to sclerostin loop3 by pull-down assay for identifying the binding residues on LRP4 to sclerostin loop3. **d** Atomic-resolution structural prediction of the interaction interface between SOST and LRP4 LA5 (P190-S226) by AlphaFold3 server, highlighting the predicted key interacting residues and their spatial arrangement (left). Heatmap depicting the minimum distances between key residues of SOST and LRP4 (right). **e** The binding affinity of LRP4 to SOST with the pretreatment of LRP4-LA5 by SPR analysis. **f** Time evolution of the center of mass distance between SOST and LRP6 under three conditions (without LRP4, with wild-type LRP4, and with LRP4m (encoding LRP4-Y200A, G201A, Y208A, H209A, C210A). **g** Root Mean Square Fluctuation (RMSF) analysis of SOST (left) and LRP6 (right) with different conditions (without LRP4, with wild-type LRP4, and with LRP4m). **h** The influence of *Lrp4m* (encoding LRP4-Y200A, G201A, Y208A, H209A, C210A) in the antagonistic effects of sclerostin on Wnt/β-catenin signaling in MC3T3-E1 cells. **i** The influence of *Lrp4m* in the antagonistic effects of sclerostin on ALP activity (the left upper panel) and mineralized nodule formation (the left lower panel) in MC3T3-E1 cells. Bar chat of ALP activity (middle) and Alizarin Red S Staining (right). **j** The influence of LRP4-Pep (LA5, P190-S226, PCNLEEFQCAYGRCILDIYHCDGDDDCGDWSDESDCS) in the antagonistic effects of sclerostin on Wnt/β-catenin signaling in MC3T3-E1 cells. **k** The influence of LRP4-Pep in the antagonistic effects of sclerostin on ALP activity (the left upper panel) and mineralized nodule formation (the left lower panel) in MC3T3-E1 cells. Bar chat of ALP activity (middle) and Alizarin Red S Staining (right). The unpaired *t*-test was used to determine the intergroup differences. ^ns^*P* > 0.05; *P < 0.05; **P < 0.01; ***P < 0.001; ****P < 0.000 1

To investigate how sclerostin loop3 participates in the antagonistic effect of sclerostin on bone formation, we identified the transmembrane receptor of sclerostin loop3 in osteoblasts (MC3T3-E1 cells) by combining pull-down assay and surface plasmon resonance (SPR). In pull-down assay, MC3T3-E1 cell lysates were incubated with Ni-NTA Magnetic Agarose beads carrying full-length His-sclerostin (His-SOST) and His-sclerostin loop3 (His-loop3) peptide, respectively. LRP4 was pulled down by both His-SOST and His-loop3 (Fig. 1a), suggesting that sclerostin loop3 bound to LRP4 in osteoblasts. Additionally, SPR analysis validated the binding of LRP4 to sclerostin (*K*_*d*_ = 37.2 nmol/L) and sclerostin loop3 (*K*_*d*_ = 12.5 nmol/L), respectively (Fig. 1b). The binding of sclerostin to LRP4 was not detected after pretreating sclerostin with our tailored-made sclerostin loop3-specific aptamer Apc001^9^ (Fig. 1b). Further, neither interaction between sclerostin loop3 and LRP6 nor interaction between sclerostin loop2 and LRP4 was detected by SPR analysis (Fig. S1a).

### To determine whether the interaction between sclerostin loop3 and LRP4 was required by sclerostin to antagonize Wnt/β-catenin signaling and inhibit bone formation

We identified the interaction residues within LRP4 to sclerostin loop3, developed *Lrp4* mutation tool (*Lrp4m*) and blocking peptide tool (LRP4-Pep) to block sclerostin loop3-LRP4 interaction for the following structure-function studies.

#### Identification of the interaction residues within LRP4 to sclerostin loop3

The interaction of sclerostin/sclerostin loop3 to full-length (FL) and truncated LRP4 (Table S1) in MC3T3-E1 lysates were analyzed, respectively, by pull-down assay. The data showed that sclerostin loop3 bound to LA5 domain (LDL-receptor class A5, P190-S226) within LRP4 (Fig. S1b). Further, SPR analysis validated the binding of LRP4-LA5 to sclerostin (*K*_*d*_ = 14.3 nmol/L) and sclerostin loop3 (*K*_*d*_ = 13.5 nmol/L) (Fig. S1c). Subsequently, the interaction of sclerostin/sclerostin loop3 to LRP4 muteins with series mutations on residues within LRP4-LA5 (Table S2) were analyzed by pull-down assay. The data showed that the binding ability of sclerostin/sclerostin loop3 to LRP4-m46 (Y200A, G201A, L205A, D206A, I207A), LRP4-m47 (Y200A, G201A, Y208A, H209A, C210A) and LRP4-m67 (L205A, D206A, I207A, Y208A, H209A, C210A) was dramatically weaker than that of sclerostin/sclerostin loop3 to wild-type LRP4 (Fig. 1c and Fig. S1d). It suggested that Y200, G201, L205, D206, I207, Y208, H209 and C210 were the key residues within LRP4 for interaction between sclerostin loop3 and LRP4, which was validated by atomic-resolution structural prediction using AlphaFold3 server (Fig. 1d).

#### Lrp4 mutation tool for genetic blockade of sclerostin loop3-LRP4 interaction

The effects of the *Lrp4* mutations, including *Lrp4-m46* (encoding LRP4-Y200A, G201A, L205A, D206A, I207A), *Lrp4-m47* (encoding LRP4-Y200A, G201A, Y208A, H209A, C210A) and *Lrp4-m67* (encoding LRP4-L205A, D206A, I207A, Y208A, H209A, C210A), on Wnt/β-catenin signaling were determined in MC3T3-E1 cells in vitro, in the absence of sclerostin. There were no significant differences in the TOP-Wnt luciferase signaling between cells transfected with wild-type *Lrp4* plasmids and cells transfected with *Lrp4-m47* plasmids in vitro, in the absence of sclerostin (Fig. S2a). Accordingly, *Lrp4-m47* mutation was named as *Lrp4m* for genetically blocking sclerostin loop3-LRP4 interaction in the following studies.

#### LRP4 peptide tool for pharmacologic blockade of sclerostin loop3-LRP4 interaction

SPR analysis revealed that the interaction of LRP4 to sclerostin was blocked after pretreating sclerostin with LRP4-LA5 (Fig. 1e). There were no significant differences in the TOP-Wnt luciferase signaling in osteoblasts (MC3T3-E1 cells) between with and without treatment of LRP4-LA5, in the absence of sclerostin (Fig. S2b). Hence, LRP4-LA5 (P190-S226, PCNLEEFQCAYGRCILDIYHCDGDDDCGDWSDESDCS) was named as LRP4-Pep for pharmacologic blockade of sclerostin loop3-LRP4 interaction in the following studies.

### Genetically, *Lrp4m* mutation reduced sclerostin-LRP6 binding, inhibited the antagonistic effects of sclerostin on Wnt/β-catenin signaling and osteogenic potential in osteoblasts in vitro

It was reported that sclerostin bound to LRP5/6 as well as LRP4, and antagonized the canonical Wnt/β-catenin signaling pathway.^18^ Osteoblastic LRP4 facilitated the binding of sclerostin to LRP6 for inhibiting bone formation.^16,17^ In this study, the binding of sclerostin to both LRP4 and LRP6 in osteoblasts were observed by confocal microscopy in vitro. Notably, the binding of sclerostin to LRP6 in osteoblasts was dramatically reduced upon *Lrp4m* (Fig. S3). It indicated that the binding of sclerostin to LRP6 was dependent on sclerostin loop3-LRP4 binding.

Moreover, molecular dynamics (MD) simulation and root mean square fluctuations (RMSF) analysis were performed to determine the effect of *Lrp4m* on the center of mass distance and stability between sclerostin and LRP6, respectively. In MD simulation, the center of mass distance between sclerostin and LRP6 was lower with the presence of wild-type LRP4 (~32 Å), when compared to the LRP4-absent system and the LRP4m mutein (LRP4-Y200A, G201A, Y208A, H209A, C210A) system (~35–37 Å) (Fig. 1f). Consistently in RMSF analysis, the wild-type LRP4 dramatically stabilized the interaction of sclerostin (SOST) to LRP6, whereas the LRP4-absent system and the LRP4m mutein system failed to stabilize the interaction of sclerostin (SOST) with LRP6 (Fig. 1g). Accordingly, the binding of sclerostin loop3 to LRP4 facilitated the binding of sclerostin to LRP6.

Further, to determine the effect of *Lrp4m* on Wnt/β-catenin signaling and osteogenic potential in osteoblasts, *sost*^*−/−*^ osteoblasts (MC3T3-E1 cells) were stably transfected with wild-type *Lrp4* (WT*-Lrp4*) lentiviral particles or *Lrp4m* lentiviral particles. In *sost*^*−/−*^ osteoblasts overexpressing wild-type LRP4, the TOP-Wnt luciferase signaling, the mRNA expression of β-catenin, ALP and OCN, as well as ALP activity and mineralized nodule formation were significantly decreased after treatment of recombinant sclerostin. After treatment of recombinant sclerostin, *sost*^*−/−*^ osteoblasts overexpressing LRP4m showed significantly higher TOP-Wnt luciferase signaling, higher mRNA expression of β-catenin, ALP and OCN, as well as higher ALP activity and mineralized nodule formation than *sost*^*−/−*^ osteoblasts overexpressing wild-type LRP4 (Fig. 1h and i, Fig. S4a). It indicated that the antagonistic effects of sclerostin on Wnt/β-catenin signaling and osteogenic potential were attenuated upon *Lrp4m*.

Together, *Lrp4m*-induced genetic blockade of sclerostin loop3-LRP4 interaction reduced sclerostin-LRP6 binding, inhibited the antagonistic effects of sclerostin on Wnt/β-catenin signaling and osteogenic potential in osteoblasts in vitro.

### Pharmacologically, LRP4-Pep pretreatment inhibited the antagonistic effects of sclerostin on Wnt/β-catenin signaling and osteogenic potential in osteoblasts in vitro

The *sost*^*−/−*^ osteoblasts (MC3T3-E1 cells) were stably transfected with wild-type *Lrp4* (WT*-Lrp4*) lentiviral particles, then treated by recombinant sclerostin (mScl) with/without pretreatment of LRP4-Pep. The data showed that the antagonistic effect of sclerostin on Wnt/β-catenin signaling in osteoblasts was dramatically attenuated with the pretreatment of the exogenous LRP4-Pep in vitro (Fig. 1j). Moreover, the antagonistic effects of sclerostin on the mRNA expression of β-catenin, ALP and OCN, as well as ALP activity and mineralized nodule formation in osteoblasts were significantly attenuated with the pretreatment of the exogenous LRP4-Pep in vitro (Fig. 1k, Fig. S4b). It indicated that LRP4-Pep-induced pharmacologic blockade of sclerostin loop3-LRP4 interaction inhibited the antagonistic effects of sclerostin on Wnt/β-catenin signaling and osteogenic potential in osteoblasts in vitro.

### *Lrp4m* promoted bone formation in *Lrp4m* mice

**Fig. 2:**
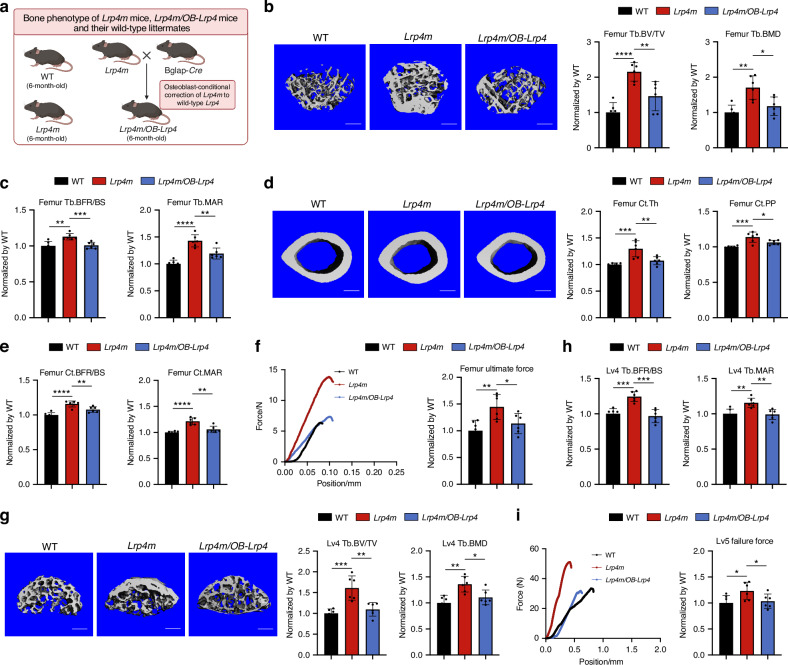
The bone phenotypes of *Lrp4m* mice, *Lrp4m/OB-Lrp4* mice and WT littermates. **a** The diagram of experimental design. **b** Representative images showing three-dimensional trabecular bone microarchitecture by *micro-CT* reconstruction at the distal femur of *Lrp4m* mice, *Lrp4m/OB-Lrp4* mice and WT littermates. Scale bars, 100 μm (the left panel). Bar charts of the structural parameters of Tb.BV/TV and Tb.BMD from ex vivo *micro-CT* examination at the distal femur (the right panel). **c** Analysis of dynamic bone histomorphometric parameters of Tb.BFR/BS and Tb.MAR at the distal femur. **d** Representative images showing three-dimensional cortical bone microarchitecture by *micro-CT* reconstruction at the femoral mid-shaft. Scale bars, 100 μm (the left panel). Bar charts of the structural parameters of Ct.Th and Ct.PP from *ex vivo micro-CT* examination at the femoral mid-shaft (the right panel). **e** Analysis of dynamic bone histomorphometric parameters of Ct.BFR/BS and Ct.MAR at the femoral mid-shaft. **f** Representative curves showing the mechanical properties of the femora, determined by three-point bending test (left). Bar charts of femur ultimate force (right). **g** Representative images showing three-dimensional trabecular bone microarchitecture by *micro-CT* reconstruction at the fourth lumbar vertebrae (Lv4). Scale bars, 100 μm (the left panel). Bar charts of the structural parameters of Tb.BV/TV and Tb.BMD from ex vivo *micro-CT* examination at the Lv4 (the right panel). **h** Analysis of dynamic bone histomorphometric parameters of Tb.BFR/BS and Tb.MAR at the Lv4. **i** Representative curves showing the mechanical properties of the fifth lumbar vertebrae (Lv5), determined by compression test (left). Bar charts of the failure force of the Lv5 (right). Data were expressed as mean ± standard deviation. *n* = 6 per group. The unpaired t-test was used to determine the intergroup differences. ^ns^*P* > 0.05; *P < 0.05; **P < 0.01; ***P < 0.001; ****P < 0.000 1

To determine the effect of osteoblastic *Lrp4m* on bone formation in vivo, we initially aimed to generate osteoblast-conditional *Lrp4m* mouse model. However, technical limitations hindered its development. As an alternative approach, we developed systematic *Lrp4m* mouse model (encoding LRP4*-*Y200A, G201A, Y208A, H209A, C210A) (Fig. 2a and Figs. S5 and S6a). *Micro-CT* analysis and bone histomorphometric analysis were utilized for the measurement of trabecular bone (below the growth plate) at the metaphysis of the distal femur, the fourth lumbar vertebrae and the proximal tibia, as well as the cortical bone at the femoral mid-shaft in *Lrp4m* mice and the wild-type littermates. The three-point bending test was used to determine the mechanical properties of the femora in *Lrp4m* mice and the wild-type littermates, while the compression test was used to determine the mechanical properties of the fifth lumbar vertebrae. Enzyme-Linked Immunosorbent Assay (ELISA) was used to determine the serum levels of bone formation biomarkers.

In *micro-CT* analysis, the data showed that *Lrp4m* mice had significantly higher trabecular bone volume ratio (Tb.BV/TV, +115.0%, ****P < 0.000 1), trabecular volumetric bone mineral density (Tb.BMD, +70.3%, **P < 0.01), trabecular bone thickness (Tb.Th, +72.6%, *P < 0.05) and lower trabecular bone spacing (Tb.Sp, −42.1%, ***P < 0.001) at the trabecular bone of distal femur, higher Tb.BV/TV (+61%, ***P < 0.001), Tb.BMD ( + 35.9%, **P < 0.01), Tb.Th ( + 36.7%, **P < 0.01) and lower Tb.Sp (−31.9%, **P < 0.01) at the trabecular bone of the fourth lumbar vertebrae (Lv4), as well as higher Tb.BV/TV ( + 56.9%, **P < 0.01), Tb.BMD ( + 43.1%, **P < 0.01), Tb.Th ( + 40.2%, *P < 0.05) and lower Tb.Sp (−34.3%, *P < 0.05) at the trabecular bone of proximal tibia, in comparison with their wild-type littermates (Fig. 2b and g, Fig. S7a, e and h). In addition, the *Lrp4m* mice had significantly higher cortical bone thickness (Ct.Th, +29.8%, ***P < 0.001) and cortical bone periosteal perimeter (Ct.PP, +13.7%, ***P < 0.001) at the cortical bone of femoral mid-shaft, when compared to their wild-type littermates (Fig. 2d).

Consistently, the bone histomorphometric analysis data showed that the *Lrp4m* mice had significantly higher bone formation rate (Tb.BFR/BS, +12.8%, **P < 0.01) and higher mineral apposition rate (Tb.MAR, +42.9%, ****P < 0.000 1) at the trabecular bone of distal femur, higher Tb.BFR/BS ( + 24.3%, ***P < 0.001) and higher Tb.MAR ( + 15.6%, **P < 0.01) at the trabecular bone of Lv4, as well as higher Tb.BFR/BS ( + 51.0%, *P < 0.05) and higher Tb.MAR ( + 58.6%, ***P < 0.001) at the trabecular bone of proximal tibia, when compared to their wild-type littermates (Fig. 2c and h, Fig. S7b, f and i). Moreover, the *Lrp4m* mice had significantly higher bone formation rate (Ct.BFR/BS, +15.8%, ****P < 0.000 1) and higher mineral apposition rate (Ct.MAR, +21.4%, ****P < 0.000 1) at the cortical bone of the femoral mid-shaft, in comparison with their wild-type littermates (Fig. 2e, Fig. S7c). It indicated that *Lrp4m* notably promoted bone formation at both trabecular bone and cortical bone in *Lrp4m* mice.

In the three-point bending test at femora, the data showed that the femur ultimate force ( + 44.5%, **P < 0.01), femur stiffness ( + 60.0%, *P < 0.05) and femur fracture energy ( +146.1%, ***P < 0.001) in *Lrp4m* mice were significantly higher than those in their wild-type littermates (Fig. 2f, Fig. S7d). In compression test at the fifth lumbar vertebrae (Lv5), the data showed that the Lv5 failure force ( + 23.2%, *P < 0.05) and Lv5 ultimate strength ( + 119.5%, ****P < 0.000 1) in *Lrp4m* mice were significantly higher than those in their wild-type littermates (Fig. 2i, Fig. S7g).

In ELISA analysis, the serum levels of bone formation biomarkers including procollagen type 1 N-terminal pro-peptide (P1NP, +65.3%, ***P < 0.001) and osteocalcin (OCN, +74.0%, **P < 0.01) were significantly higher in the *Lrp4m* mice than those in their wild-type littermates (Fig. S7j).

Together, *Lrp4m* promoted bone formation, enhanced bone mass and bone microarchitecture, improved bone mechanical properties at both trabecular bone and cortical bone in *Lrp4m* mice.

Moreover, we determined the effect of *Lrp4m* on bone resorption in vivo. The bone histomorphometric data showed that the osteoclast surface per bone surface (Oc.S/BS) at both trabecular bone of distal femur (−14.2%, *P < 0.05) and cortical bone of femoral mid-shaft (−16.2%, *P < 0.05) were significantly lower in *Lrp4m* mice, when compared to their wild-type littermates. In ELISA analysis, the serum level of β-CTX was lower (−21.3%, ^ns^*P* > 0.05) in *Lrp4m* mice, in comparison with their wild-type littermates (Fig. S8).

Further, we determined the effect of *Lrp4m* on muscle in vivo by the maximal forelimb grip strength test, gastrocnemius muscle-to-body weight ratio assay (M/B ratio), and muscle fiber cross-sectional area (CSA) measurement. No differences were determined in the maximal forelimb grip strength, gastrocnemius M/B ratio or muscle fiber CSA between *Lrp4m* mice and their wild-type littermates (Fig. S9).

### *Lrp4m/OB-Lrp4* mice (Osteoblast-conditional correction of *Lrp4m* to wild-type *Lrp4*) had significantly lower bone formation than *Lrp4m* mice

To determine whether the promotive effects of *Lrp4m* on bone formation would act in osteoblasts in vivo, we crossbred *Lrp4m* mice with Bglap-Cre mice to develop the *Lrp4m*/OB-*Lrp4* mice (OB: osteoblasts), in which only osteoblasts had no mutations in *Lrp4* (Fig. 2a and Figs. S5 & S6b**)**.

In *micro-CT* analysis, the *Lrp4m/OB-Lrp4* mice had significantly lower Tb.BV/TV (−32.0%, **P < 0.01), Tb.BMD (−30.9%, *P < 0.05), Tb.Th (−19.3%, *P < 0.05) and higher Tb.Sp ( + 23.9%, *P < 0.05) at the trabecular bone of distal femur, significantly lower Tb.BV/TV (−31.9%, **P < 0.01), Tb.BMD (−18.6%, *P < 0.05), Tb.Th (−26.8%, **P < 0.01) and higher Tb.Sp ( + 56.6%, **P < 0.01) at the trabecular bone of the Lv4, as well as significantly lower Tb.BV/TV (−24.9%, *P < 0.05), Tb.BMD (−18.3%, *P < 0.05), Tb.Th (−28.6%, **P < 0.01) and higher Tb.Sp ( + 27.6%, *P < 0.05) at the trabecular bone of proximal tibia, in comparison with *Lrp4m* mice (Fig. 2b and g, Fig. S7a, e and h**)**. In addition, the Ct.Th (−17.3%, **P < 0.01) and Ct.PP (−6.7%, *P < 0.05) at the cortical bone of the femoral mid-shaft were significantly lower in *Lrp4m/OB-Lrp4* mice than those in *Lrp4m* mice (Fig. 2d).

Consistently in bone histomorphometric analysis, the *Lrp4m/OB-Lrp4* mice had significantly lower Tb.BFR/BS (−10.8%, ***P < 0.001) and Tb.MAR (−16.5%, **P < 0.01) at trabecular bone of distal femur, significantly lower Tb.BFR/BS (−22.2%, ***P < 0.001) and Tb.MAR (−14.4%, **P < 0.01) at trabecular bone of the Lv4, as well as significantly lower Tb.BFR/BS (−27.5%, *P < 0.05) and Tb.MAR (−37.6%, ***P < 0.001) at trabecular bone of proximal tibia, when compared to *Lrp4m* mice (Fig. 2c and h, Fig. S7b, f and i). Moreover, the *Lrp4m/OB-Lrp4* mice had significantly lower Ct.BFR/BS (−7.0%, **P < 0.01) and Ct.MAR (−12.9%, **P < 0.01) at the cortical bone of femoral mid-shaft, in comparison with the *Lrp4m* mice (Fig. 2e, Fig. S7c).

In the three-point bending test at the femora, the data showed that *Lrp4m/OB-Lrp4* mice had significantly lower femur ultimate force (−21.5%, *P < 0.05), femur stiffness (−27.9%, *P < 0.05) and femur fracture energy (−30.0%, *P < 0.05) than *Lrp4m* mice (Fig. 2f, Fig. S7d). In compression test at the Lv5, the data showed that *Lrp4m/OB-Lrp4* mice had significantly lower Lv5 failure force (−16.1%, *P < 0.05) and Lv5 ultimate strength (−38.2%, **P < 0.01) than *Lrp4m* mice (Fig. 2i, Fig. S7g).

In ELISA analysis, the serum levels of P1NP (−36.7%, ***P < 0.001) and OCN (−27.2%, *P < 0.05) in *Lrp4m/OB-Lrp4* mice were significantly lower than those in *Lrp4m* mice (Fig. S7j).

### Genetically, *Lrp4m*-induced blockade of sclerostin loop3-LRP4 interaction diminished the inhibitory effect of sclerostin on bone formation in vivo

**Fig. 3:**
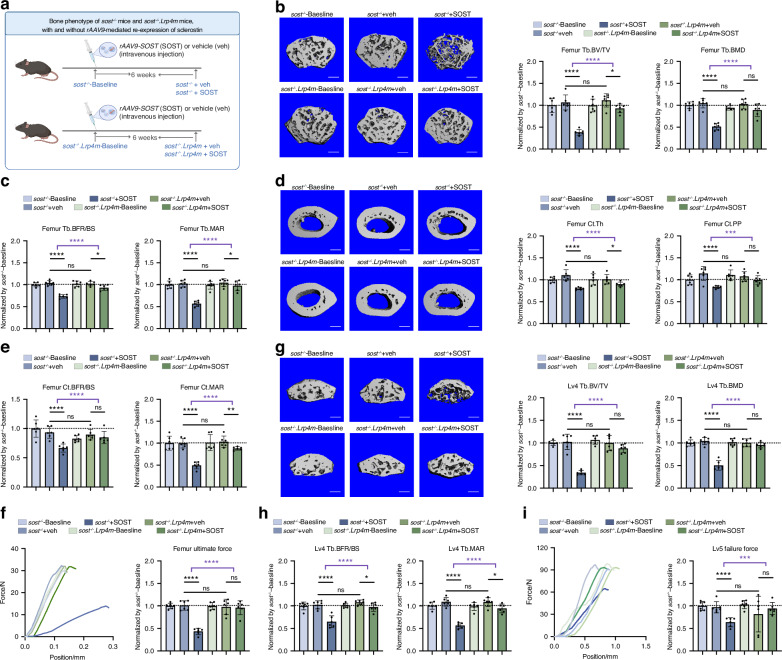
The bone formation of *sost*^*−/−*^ mice and *sost*^*−/−*^*.Lrp4m* mice, with and without *rAAV9*-mediated re-expression of sclerostin. **a** The diagram of experimental design. **b** Representative images showing three-dimensional trabecular bone microarchitecture by *micro-CT* reconstruction at the distal femur in *sost*^*−/−*^ mice and *sost*^*−/−*^*.Lrp4m* mice, with and without *rAAV9*-mediated re-expression of sclerostin (SOST). Scale bars, 100 μm (the left panel). Bar charts of the structural parameters of Tb.BV/TV and Tb.BMD from ex vivo *micro-CT* examination at the distal femur (the right panel). **c** Analysis of dynamic bone histomorphometric parameters of Tb.BFR/BS and Tb.MAR at the distal femur. **d** Representative images showing three-dimensional cortical bone microarchitecture by *micro-CT* reconstruction at the femoral mid-shaft. Scale bars, 100 μm (the left panel). Bar charts of the structural parameters of Ct.Th and Ct.PP from *ex vivo micro-CT* examination at the femoral mid-shaft (the right panel). **e** Analysis of dynamic bone histomorphometric parameters of Ct.BFR/BS and Ct.MAR at the femoral mid-shaft. **f** Representative curves showing the mechanical properties of the femora, determined by three-point bending test (left). Bar charts of the femur ultimate force (right). **g** Representative images showing three-dimensional trabecular bone microarchitecture by *micro-CT* reconstruction at the fourth lumbar vertebrae (Lv4). Scale bars, 100 μm (the left panel). Bar charts of the structural parameters of Tb.BV/TV and Tb.BMD from ex vivo *micro-CT* examination at the Lv4 (the right panel). **h** Analysis of dynamic bone histomorphometric parameters of Tb.BFR/BS and Tb.MAR at the Lv4. **i** Representative curves showing the mechanical properties of the fifth lumbar vertebrae (Lv5), determined by compression test (left). Bar charts of the failure force at the Lv5 (right). Data were expressed as mean ± standard deviation. *n* = 6 per group. The unpaired t-test was used to determine the intergroup differences; ^ns^*P* > 0.05; *P < 0.05; **P < 0.01; ***P < 0.001; ****P < 0.000 1 in black color. The unpaired t-test was also used to determine the difference between Means (*sost*^*−/−*^*.Lrp4m* + SOST) - (*sost*^*−/−*^*.Lrp4m* + veh) ± SEM *vs*. Means (*sost*^*−/−*^ + SOST) - (*sost*^*−/*−^ + veh) ± SEM; ^ns^*P* > 0.05; *P < 0.05; **P < 0.01; ***P < 0.001; ****P < 0.000 1 in purple color

To determine whether the promotive effect of *Lrp4m* on bone formation could be induced by genetic blockade of sclerostin loop3-LRP4 interaction in vivo, the *sost*^*−/−*^*.Lrp4m* mouse model was constructed by crossbreeding *Lrp4m* mice with *sost*^*−/−*^ mice for shielding the effects of endogenous sclerostin (Fig. S6c and d). There were no significant differences in bone formation, bone mass, bone microarchitecture integrity and bone mechanical properties between *sost*^*−/−*^*.Lrp4m* mice and *sost*^*−/−*^ mice (Fig. 3, Fig. S10). Then, *rAAV9-SOST* was intravenously injected in *sost*^*−/−*^*.Lrp4m* mice and *sost*^*−/−*^ mice for re-expression of sclerostin (SOST). The blank *rAAV9* vector was enrolled as negative control (vehicle: veh).

In *micro-CT* analysis, when compared to mice in *sost*^*−/−*^ *+* veh group, the *sost*^*−/−*^ mice with re-expression of sclerostin (*sost*^*−/−*^ + SOST) had significantly lower Tb.BV/TV (−64.0%, ****P < 0.000 1), Tb.BMD (−51.1%, ****P < 0.000 1), Tb.Th (−27.3%, ***P < 0.001) and higher Tb.Sp ( + 102.8%, ****P < 0.000 1) at the trabecular bone of distal femur, significantly lower Tb.BV/TV (−66.5%, ****P < 0.000 1), Tb.BMD (−51.9%, ****P < 0.000 1), Tb.Th (−36.8%, ***P < 0.00 1) and higher Tb.Sp ( + 143.8%, ****P < 0.000 1) at the trabecular bone of the Lv4, as well as significantly lower Tb.BV/TV (−51.7%, ****P < 0.000 1), Tb.BMD (−37.9%, ****P < 0.000 1), Tb.Th (−48.2%, ****P < 0.000 1) and higher Tb.Sp ( + 178.6%, ****P < 0.000 1) at the trabecular bone of proximal tibia, (Fig. 3b and g, Fig. S10a, e and h). Moreover, Ct.Th (−27.6%, ****P < 0.000 1) and Ct.PP (−27.2%, ****P < 0.000 1) at cortical bone of the femoral mid-shaft in mice from the *sost*^*−/−*^ + SOST group were significantly lower than those in mice from the *sost*^*−/−*^ + veh group (Fig. 3d).

In bone histomorphometric analysis, Tb.BFR/BS (−29.3%, ****P < 0.000 1) and Tb.MAR (−41.6%, ****P < 0.000 1) at trabecular bone of distal femur (Fig. 3c, Fig. S10b), Tb.BFR/BS (−36.8%, ****P < 0.000 1) and Tb.MAR (−48.2%, ****P < 0.000 1) at trabecular bone of the Lv4 (Fig. 3h, Fig. S10f), Tb.BFR/BS (−28.1%, ***P < 0.00 1) and Tb.MAR (−31.1%, ***P < 0.001) at trabecular bone of proximal tibia (Fig. S10i), as well as Ct.BFR/BS (−28.6%, ****P < 0.000 1) and Ct.MAR (−50.5%, ****P < 0.000 1) at cortical bone of femoral mid-shaft (Fig. 3e, Fig. S10c) were significantly lower in *sost*^*−/−*^ + SOST group, when compared to *sost*^*−/−*^ + veh group.

In the three-point bending test at the femora, the femur ultimate force (−57.1%, ****P < 0.000 1), femur stiffness (−72.6%, ****P < 0.000 1) and femur fracture energy (−35.6%, **P < 0.01) were significantly lower in *sost*^*−/−*^ + SOST group than those in *sost*^*−/−*^ + veh group (Fig. 3f, Fig. S10d). In the compression test at the Lv5, the failure force (−34.3%, ****P < 0.000 1) and ultimate strength (−50.1%, **P < 0.01) were significantly lower in *sost*^*−/−*^ + SOST group than those in *sost*^*−/−*^ + veh group (Fig. 3i, Fig. S10g).

In ELISA analysis, the serum levels of P1NP (−62.0%, ***P < 0.001) and OCN (−41.5%, **P < 0.01) were significantly lower in *sost*^*−/−*^ + SOST group than those in *sost*^*−/−*^ + veh group (Fig. S10j).

The differences in the parameters above between *sost*^*−/−*^ + SOST group and *sost*^*−/−*^ + veh group were significantly higher than the differences in the parameters above between *sost*^*−/−*^*.Lrp4m* + SOST group and *sost*^*−/−*^*.Lrp4m* + veh group (Fig. 3, Fig. S10). It indicated that *Lrp4m* diminished the inhibitory effect of sclerostin on bone formation in vivo.

### Pharmacologically, LRP4-Pep-induced blockade of sclerostin loop3-LRP4 interaction diminished the inhibitory effect of sclerostin on bone formation in vivo in a dose dependent manner

To pharmacologically determine the role of sclerostin loop3-LRP4 interaction in the inhibitory effect of sclerostin on bone formation, the first and last three amino acids of our developed LRP4-Pep were changed from L-type to D-type amino acids (end-protected) to improve the hydrolytic stability in vivo.^19,20^ The administration interval of the modified LRP4-Pep was defined as once per day, based on its elimination half-life (T_1/2_ = 5.8 h). Then, the *sost*^*−/−*^ mice were subcutaneously injected with vehicle (veh) and the exogenous LRP4-Pep (5 mg/kg, 10 mg/kg and 20 mg/kg), once per day for six weeks. There were no significant differences in parameters regarding bone mass, bone microarchitecture and bone formation in *sost*^*−/−*^ mice with/without treatment of LRP4-Pep (Fig. S11). It indicated that LRP4-Pep itself had no effect on bone formation, in the absence of sclerostin.

**Fig. 4:**
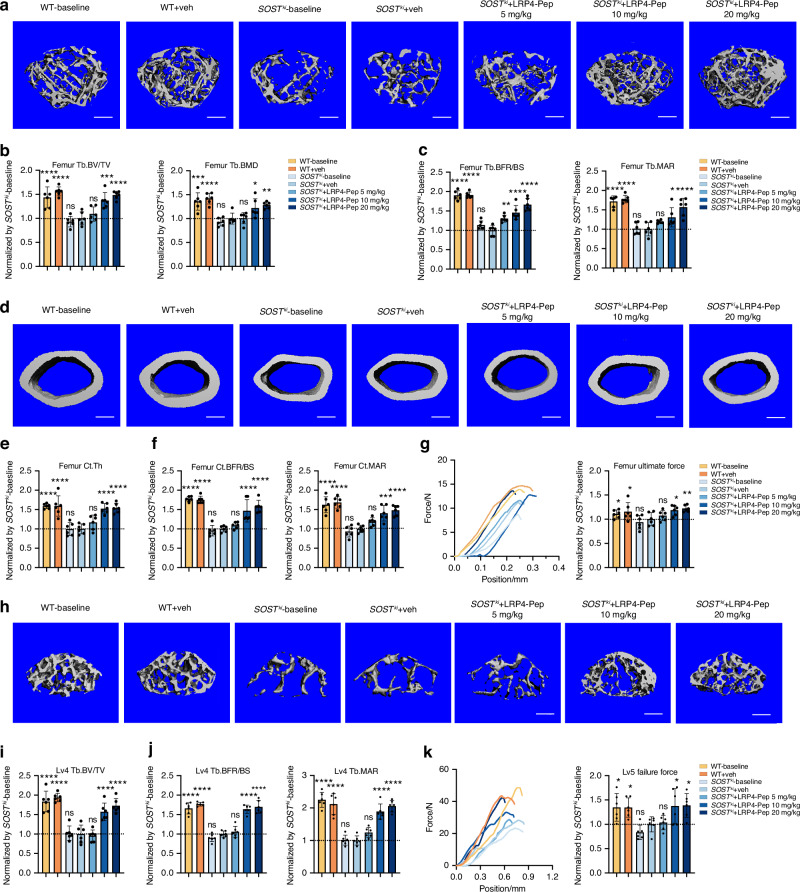
The influence of the exogenous LRP4-Pep in the antagonistic effect of sclerostin on bone formation in *SOST*^*ki*^ mice. **a** Representative images showing three-dimensional trabecular bone microarchitecture by *micro-CT* reconstruction at the distal femur in *SOST*^*ki*^ mice, with/without treatment of exogenous LRP4-Pep at a dose of 5 mg/kg, 10 mg/kg and 20 mg/kg, respectively. Scale bars, 500 μm. **b** Bar charts of the structural parameters of Tb.BV/TV and Tb.BMD from ex vivo *micro-CT* examination at the distal femur. **c** Analysis of dynamic bone histomorphometric parameters of Tb.BFR/BS and Tb.MAR at the distal femur. **d** Representative images showing three-dimensional cortical bone microarchitecture by *micro-CT* reconstruction at the femoral mid-shaft. Scale bars, 100 μm. **e** Bar charts of the structural parameter of Ct.Th from ex vivo *micro-CT* examination at the femoral mid-shaft. **f** Analysis of dynamic bone histomorphometric parameters of Ct.BFR/BS and Ct.MAR at the femoral mid-shaft. **g** Representative curves showing the mechanical properties of the femur, determined by three-point bending test (left). Bar charts of femur ultimate force (right). **h** Representative images showing three-dimensional trabecular bone microarchitecture by *micro-CT* reconstruction at the fourth lumbar vertebrae (Lv4). Scale bars, 100 μm. **i** Bar charts of the structural parameter of Tb.BV/TV from *ex vivo micro-CT* examination at the Lv4. **j** Analysis of dynamic bone histomorphometric parameters of Tb.BFR/BS and Tb.MAR at the Lv4. **k** Representative curves showing the mechanical properties of the fifth lumbar vertebrae (Lv5) by compression test (left). Bar charts of failure force at the Lv5 (right). Data were expressed as mean ± standard deviation. *n* = 6 per group. One-way ANOVA with Tukey’s post-hoc test vs. *SOST*^*ki*^ + veh group was used to determine the intergroup differences. ^ns^*P* > 0.05; *P < 0.05; **P < 0.01; ***P < 0.001; ****P < 0.000 1

The *SOST*^*ki*^ mice (Fig. S6e) were subcutaneously injected with vehicle (veh) and the exogenous LRP4-Pep (5 mg/kg, 10 mg/kg and 20 mg/kg), once per day for six weeks (Fig. S12a). In *micro-CT* analysis, Tb.BV/TV, Tb.BMD and Tb.Th at trabecular bone of distal femur (Fig. 4a and b, Fig. S12b), the Lv4 (Fig. 4h and i, Fig. S12f) and proximal tibia (Fig. S12i) in *SOST*^*ki*^ mice were significantly enhanced after treatment of exogenous LRP4-Pep, while Tb.Sp was dramatically decreased, in a dose dependent manner. In addition, Ct.Th at cortical bone of the femoral mid-shaft in *SOST*^*ki*^ mice were significantly enhanced after treatment of exogenous LRP4-Pep, in a dose dependent manner (Fig. 4d and e).

In bone histomorphometric analysis, Tb.BFR/BS and Tb.MAR at trabecular bone of distal femur (Fig. 4c, Fig. S12c), the Lv4 (Fig. 4j, Fig. S12g), and proximal tibia (Fig. S12j), as well as Ct.BFR/BS and Ct.MAR at cortical bone of femoral mid-shaft (Fig. 4f, Fig. S12d) in *SOST*^*ki*^ mice were significantly enhanced after treatment of exogenous LRP4-Pep, in a dose dependent manner.

In the three-point bending test at femora, the femur ultimate force, femur stiffness and femur fracture energy in *SOST*^*ki*^ mice were significantly enhanced after treatment of exogenous LRP4-Pep, in a dose dependent manner (Fig. 4g, Fig. S12e). In the compression test at the Lv5, the failure force and ultimate strength in *SOST*^*ki*^ mice were significantly enhanced after treatment of exogenous LRP4-Pep, in a dose dependent manner (Fig. 4k, Fig. S12h).

In ELISA analysis, the serum levels of P1NP and OCN in *SOST*^*ki*^ mice were significantly enhanced after treatment of exogenous LRP4-Pep, in a dose dependent manner (Fig. S12k).

Further, we determined the effect of LRP4-Pep on muscle in vivo by the maximal forelimb grip strength test, gastrocnemius muscle-to-body weight ratio (M/B ratio) assay, and muscle fiber cross-sectional area (CSA) measurement. No differences were determined in the maximal forelimb grip strength, gastrocnemius M/B ratio or muscle fiber CSA in wild-type mice between with and without LRP4-Pep treatment (Fig. S9).

### LRP4-Pep promoted bone formation, increased bone mass and improved bone microarchitecture integrity in OVX mice with established osteoporosis

To further determine the effect of LRP4-Pep on bone mass and bone microarchitecture in ovariectomized (OVX) mice with established osteoporosis in vivo, OVX mice were treated accordingly (LRP4-Pep 10 mg/kg/day or saline). Micro-CT was conducted for measurement of the trabecular bone at the distal femur, as well as the cortical bone at the femoral mid-shaft. Before treatment, the Tb.BV/TV, Tb.vBMD and Tb.Th at trabecular bone of the distal femur were −21.1% (***P < 0.001), −24.7% (***P < 0.001) and −13.2% (*P < 0.05) lower, Tb.Sp was +19.8% (**P < 0.01) higher in the OVX baseline (OVX-Baseline) group than those in the SHAM baseline (SHAM-Baseline) group, indicating that the osteoporosis was established with substantially lower trabecular bone mass and worse trabecular bone microarchitecture in OVX mice. Compared to mice in OVX + veh group, mice in OVX + LRP4-Pep group had significantly higher Tb.BV/TV ( + 17.2%, *P < 0.05), Tb.BMD ( + 24.1%, *P < 0.05) and Tb.Th ( + 14.5%, **P < 0.01) at trabecular bone of distal femur, and lower Tb.Sp (−15.5%, **P < 0.01) (Fig. S13a). In addition, before treatment, the Ct.Th at cortical bone of the femoral mid-shaft were −10.8% (**P < 0.01) lower in the OVX-Baseline group than that in the SHAM-Baseline group, indicating that osteoporosis was established with substantially lower cortical bone mass in OVX mice. Compared to mice in OVX + veh group, mice in OVX + LRP4-Pep group had +9.4% (*P < 0.05) higher Ct.Th at cortical bone of the femoral mid-shaft (Fig. S13b).

To determine the effect of LRP4-Pep on bone formation in OVX mice, bone histomorphometric analysis was conducted. Before treatment, the Tb.BFR/BS and Tb.MAR at trabecular bone of the distal femur were −27.6% (****P < 0.000 1), −16.9% (***P < 0.001) lower in the OVX-Baseline group than those in the SHAM-Baseline group. After treatment, mice in OVX + LRP4-Pep group had +16.7% (**P < 0.01) higher Tb.BFR/BS and +20.3% (**P < 0.01) higher Tb.MAR at trabecular bone of distal femur than mice in OVX + veh group (Fig. S13c). In addition, before treatment, the Ct.BFR/BS and Ct.MAR at cortical bone of the femoral mid-shaft were −18.2% (***P < 0.001) and −10.0% (*P < 0.05) lower in the OVX-Baseline group than those in the SHAM-Baseline group. After treatment, mice in OVX + LRP4-Pep group had +9.2% (*P < 0.05) higher Ct.BFR/BS and +12.5% (**P < 0.01) higher Ct.MAR at cortical bone of femoral mid-shaft than mice in OVX + veh group (Fig. S13d).

## Discussion

**Fig. 5:**
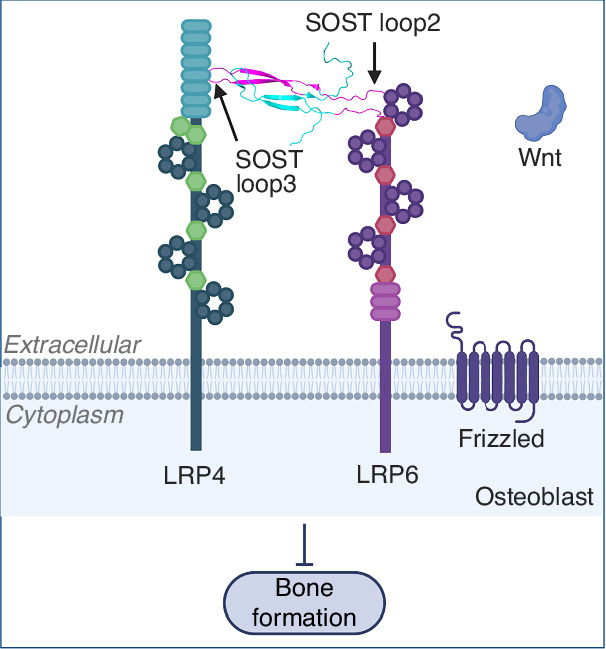
Schematic diagram showing our findings on the important anchor role of osteoblastic sclerostin loop3-LRP4 interaction in facilitating scleorstin-LRP6 interaction, antagonizing Wnt/β-catenin signaling and inhibiting bone formation

Although it was known that sclerostin bound to LRP4 and that LRP4 was required by sclerostin to inhibit bone formation,^21^ the precise mechanism regarding which specific domain or region within sclerostin/LRP4 is involved in this process remains unclear. For the first time in this work, sclerostin loop3 was identified to bind to LRP4, rather than LRP6, in osteoblasts. On the other hand, sclerostin loop2 bound to LRP6, rather than LRP4, in osteoblasts. Both genetic studies by *Lrp4m*-based approaches and pharmacologic studies by LRP4-Pep-based approaches consistently indicated that osteoblastic sclerostin loop3-LRP4 interaction, as an anchor, was required by sclerostin to bind to LRP6, thereby antagonizing Wnt/β-catenin signaling in osteoblasts in vitro, as well as inhibiting bone formation, decreasing bone mass, impairing bone microarchitecture, and reducing bone strength in vivo (Fig. 5).

Sclerostin was known to antagonize bone anabolic Wnt/β-catenin signaling pathway in osteoblasts through the interaction between sclerostin loop2-NxI motif and the first YWTD β-propeller domain of LRP5/6.^12^ LRP4 is another single-spanning transmembrane protein of the LDLR-related protein family that shares the structure of four tandem YWTD β-propeller/EGF-like repeats and LDL receptor class A (LA) repeats as LRP5/6, but differs in structural organization and function.^17,22^ The *Lrp4*-null or osteoblastic mutation elevated bone formation, increased trabecular and cortical bone mass in mice,^15^ indicating the critical role of LRP4 in inhibiting bone formation. As a postulated anchor, osteoblastic LRP4 facilitated the binding of sclerostin to LRP6 for inhibiting bone formation.^16,17^ Nevertheless, how LRP4 facilitated the binding of sclerostin to LRP6 remains unclear. In this study, we advanced the knowledge that sclerostin loop3, rather than sclerostin loop2, bound to LRP4 in osteoblasts. On the other hand, sclerostin loop2, rather than sclerostin loop3, bound to LRP6 in osteoblasts. Unlike sclerostin loop2 which bound to the first YWTD β-propeller domain of LRP6,^12^ sclerostin loop3 was notably identified to bind to the fifth LA repeat (LA5) of LRP4. Sclerostin loop3-LRP4 interaction in osteoblasts, as an anchor, facilitated the binding of sclerostin to LRP6, thereby antagonizing Wnt/β-catenin signaling and inhibiting bone formation both in vitro and in vivo. The above findings were consistent with our published data that targeting sclerostin loop3 by our tailor-made sclerostin loop3 aptamer Apc001 to block sclerostin loop3-LRP4 interaction promoted bone formation, while had no influence in aortic inflammation, atherosclerosis and aortic aneurysm progression in mouse models.^9,10^ High bone mass was reported in patients with *Lrp4* gene mutations (R1170W, R1170Q and W1186S) in the third YWTD β-propeller domain.^23^ In our molecular dynamics simulation, both R1170W mutation and W1186S mutation could dramatically reduce sclerostin loop3-LRP4 LA5 interaction, which could explain the high bone phenotype of the gene mutation carriers (Fig. S14).

The marketed therapeutic antibody mainly targeting sclerostin loop2 for postmenopausal osteoporosis may have caused severe cardiovascular events in clinical trials.^2–7,24^ Due to the cardiovascular risk, both US-FDA and EMA restrict its clinical use (FDA Press Announcements, European Medicines Agency Documents). In one nonclinical study, the therapeutic sclerostin antibody was reported to have no effect on the total atherosclerosis plaque volume in *ApoE*^*−/−*^ mice with AngII infusion.^8^ In another nonclinical study reported by US-FDA, the therapeutic sclerostin antibody increased the incidence of plaques with necrosis of all types (2–5) and upregulated the local expression of inflammatory cytokines and chemokines in ovariectomized (OVX) *ApoE*^*−/−*^ mice with high-fat diet. Despite continuing controversy, cardiovascular risk of sclerostin inhibition cannot be definitively ruled out. In our published work,^9,10^ it was found that sclerostin participated in inhibiting bone formation and preventing cardiovascular events via different loops. Both sclerostin loop2 and sclerostin loop3 participated in the inhibitory effect of sclerostin on bone formation, while the cardiovascular events preventive effects of sclerostin in *ApoE*^*−/−*^ mice were independent of sclerostin loop3. Sclerostin loop2 was critical in the cardiovascular protective action of sclerostin, evidenced by the data that specific blockade of sclerostin antibody-sclerostin loop2 interaction attenuated the sclerostin antibody-caused aggravation of inflammatory responses, atherosclerosis and aortic aneurysm in *ApoE*^*−/−*^ mice. Although osteoblastic sclerostin loop2-LRP6 interaction was known to be critical for inhibiting bone formation, the cardiovascular risk likely limited the clinical application of inhibitors targeting sclerostin loop2. The *ApoE*^*−/−*^ mouse model induces atherosclerosis, which could be the core and primary pathological basis of the cardiovascular events (myocardial infarction/cardiac ischemic events) observed in patients with treatment of Romosozumab (BRIDGE and ARCH)^2–7,24^ (Atherosclerotic plaques in the arterial wall would be destabilized and ruptured, thus triggering myocardial infarction/cardiac ischemic events^25,26^). However, the *ApoE*^*−*^ mouse model may not fully mimic the cardiovascular events observed in humans. Therefore, clinical trials need to be designed to observe the cardiovascular responses of *ApoE*-mutant populations to the marketed antibody against sclerostin loop2. In addition, *ApoE* mutations in patients in phase III clinical trials of the marketed antibody against sclerostin loop2 warrant retrospective analysis. It could facilitate developing clinical guidelines for precision medicine with the marketed sclerostin antibody, e.g., which patients might benefit from it and which patients might be at a high risk of cardiovascular events.

Our work refined the structure and function mode between sclerostin and LRP4/5/6 in osteoblast, discovered the important anchor role of sclerostin loop3-LRP4 interaction in facilitating scleorstin-LRP6 interaction and inhibiting bone formation. Translationally, either targeting sclerostin loop3 or targeting the potential binding pockets within LRP4 LA5^27^ (Fig. S15) to specifically block sclerostin loop3-LRP4 interaction could be the next generation of precise therapeutic strategies to promote bone formation with no safety concern in cardiovascular system.

## Materials and methods

### Experimental design

#### Study 1: Analysis of the interaction between sclerostin and LRP4 in osteoblasts

The transmembrane receptor for sclerostin loop3 in osteoblasts (MC3T3-E1 cells) were identified by pull-down assay. Then, the binding of LRP4/LRP6 to sclerostin loop3, sclerostin loop2 and full-length sclerostin were determined by surface plasmon resonance (SPR) analysis. Further, the interaction residues within LRP4 to sclerostin loop3 were identified in combination with pull-down assay and SPR analysis. The interaction mode was predicted by AlphaFold3 server.

#### Study 2: Determination of whether sclerostin loop3-LRP4 interaction was required by sclerostin to antagonize Wnt/β-catenin signaling and osteogenic potential in osteoblasts in vitro

Based on the identified interaction residues within LRP4 to sclerostin loop3, a series of *Lrp4* mutations and LRP4 peptides were developed. All wild-type/mutant proteins and peptides in this study were of murine origin. After identifying the mutation and peptide that did not inherently alter Wnt/β-catenin signaling and osteogenic potential in osteoblasts (MC3T3-E1 cells), *Lrp4m* (genetic tool) and LRP4-Pep (pharmacological tool) were designed to block sclerostin loop3-LRP4 interaction in the following studies.

Firstly, the binding of sclerostin loops to LRP4 or LRP6 in osteoblasts (MC3T3-E1 cells) were observed by confocal microscopy in vitro. Then, molecular dynamics simulation and root mean square fluctuations (RMSF) analysis were performed to determine the effect of *Lrp4m* on the center of mass distance and stability between sclerostin and LRP6, respectively.

To determine the effect of *Lrp4m* on Wnt/β-catenin signaling and osteogenic potential in osteoblasts, *sost*^*−/−*^ MC3T3-E1 cells were divided into 4 groups (*n* = 3 per group), including (1) WT-*Lrp4*, (2) *Lrp4m*, (3) WT-*Lrp4 +* mScl, and (4) *Lrp4m +* mScl. The cells in group (1) and (3) were stably transfected with wild-type *Lrp4* (WT-*Lrp4*) lentiviral particles. The cells in group (2) and (4) were stably transfected with *Lrp4m* lentiviral particles. 48 h later, cells in group (3) and (4) were treated with recombinant sclerostin (mScl, 50 nmol/L). To determine the effect of LRP4-Pep on Wnt/β-catenin signaling and osteogenic potential in osteoblasts, *sost*^*−/−*^ MC3T3-E1 cells were divided into 4 groups (*n* = 3 per group), stably transfected with wild-type *Lrp4* (WT-*Lrp4*) lentiviral particles, then treated with PBS, LRP4-Pep, recombinant sclerostin with PBS (mScl + veh) or recombinant sclerostin with LRP4-Pep (mScl + LRP4-Pep). All groups of cells were collected after 48 h for determination of Wnt/β-catenin signaling by TOP-Wnt-induced luciferase reporter assay, after 3 days for determination of mRNA expression levels of β-catenin, after 7 days for determination of mRNA or protein expression levels of ALP by RT-PCR or ALP staining, or after 21 days for determination of mRNA or protein expression levels of OCN by RT-PCR or Alizarin Red S solution staining, respectively.^9^

#### Study 3: Genetically determining whether osteoblastic sclerostin loop3-LRP4 interaction was required by sclerostin to inhibit bone formation in vivo

To determine the effect of osteoblastic *Lrp4m* on bone formation in vivo, we initially aimed to generate an osteoblast-conditional *Lrp4m* mouse model. However, technical limitations hindered its development. As an alternative approach, we developed systematic *Lrp4m* mice (C57BL/6 J). Then, *Lrp4m* mice were further crossbred with Bglap-Cre mice to construct the *Lrp4m/OB-Lrp4* mouse model (OB: osteoblasts; C57BL/6 J) in which *Lrp4m* in osteoblasts was corrected to wild-type *Lrp4*.

6-month-old male *Lrp4m* mice, *Lrp4m/OB-Lrp4* mice and wild-type (WT) littermates (*n* = 6 per group) were euthanatized. Serum was collected for determination of the expression levels of bone formation markers and bone resorption markers.^28,29^ The right femur, right tibia, and the fourth vertebrae (Lv4) were fixed with 4% PFA, respectively, for examination of the bone mass and bone microarchitecture by *Micro-CT* analysis.^9,10^ Subsequently, the above undecalcified samples were collected for bone histomorphometric analysis.^10,30^ The left femurs and the fifth lumbar vertebrae (Lv5) were wrapped with PBS-soaked gauze and stored at −80 °C for further examination of the bone mechanical properties by three-point bending test and compression test, respectively.^9,10,31^ Meanwhile, the muscle phenotype of *Lrp4m* mice and their wild-type littermates were determined by maximal forelimb grip strength test, gastrocnemius muscle-to-body weight ratio (M/B ratio) assay and muscle fiber cross-sectional area (CSA) measurement.^32,33^

To further determine whether the promotive effect of *Lrp4m* on bone formation was induced by genetic blockade of sclerostin loop3-LRP4 interaction in vivo, the *sost*^*−/−*^*.Lrp4m* mouse model (C57BL/6 J) was constructed by crossbreeding *Lrp4m* mice with *sost*^*−/−*^ mice for shielding the effects of endogenous sclerostin (SOST). Then, *rAAV9-SOST* was intravenously injected in 6-month-old male *sost*^*−/−*^*.Lrp4m* mice and *sost*^*−/−*^ mice, respectively, for re-expression of sclerostin (SOST).^34^ The groups were as follows (*n* = 6 per group): (1) *sost*^*−/−*^-Baseline, (2) *sost*^*−/−*^ + veh, (3) *sost*^*−/−*^ + SOST, (4) *sost*^*−/−*^*.Lrp4m*-Baseline, (5) *sost*^*−/−*^*.Lrp4m* + veh and (6) *sost*^*−/−*^*.Lrp4m* + SOST. For group (1) and group (4), mice were euthanatized at 6-month-old. For the other groups, mice were euthanatized six weeks after injection of *rAAV9-SOS*T (SOST) or vehicle (veh). Serum, distal femur, proximal tibia and vertebrae of mice in each group were harvested for analysis as described above.^9,10,28–31^

#### Study 4: Pharmacologically validating whether sclerostin loop3-LRP4 interaction was required by sclerostin to inhibit bone formation in vivo

To pharmacologically determine the role of sclerostin loop3-LRP4 interaction in the inhibitory effect of sclerostin on bone formation, the first and last three amino acids of our developed LRP4-Pep were changed from L-type to D-type amino acids (end-protected) to improve the hydrolytic stability in vivo.^19,20^ The administration interval of the modified LRP4-Pep was defined as once per day, based on its elimination half-life (T_1/2_ = 5.8 h). The 6-month-old male *sost*^*−/−*^ mice were subcutaneously injected with vehicle (veh) and the exogenous LRP4-Pep (5 mg/kg, 10 mg/kg and 20 mg/kg), once per day for six weeks. It was determined that LRP4-Pep itself had no effect on bone formation in *sost*^*−/−*^ mice, in the absence of sclerostin. Subsequently, the 6-month-old male *SOST*^*ki*^ mice and wild-type (C57BL/6 J) mice were randomly divided as the following groups (*n* = 6 per group): (1) WT-Baseline, (2) WT + veh, (3) *SOST*^*ki*^-Baseline, (4) *SOST*^*ki*^ + veh, (5) *SOST*^*ki*^ + LRP4-Pep 5 mg/kg, (6) *SOST*^*ki*^ + LRP4-Pep 10 mg/kg and (7) *SOST*^*ki*^ + LRP4-Pep 20 mg/kg. For group (1) and (3), mice were sacrificed at 6 months old. For the remaining groups, *SOST*^*ki*^ mice or wild-type mice were subcutaneously injected with vehicle or exogenous LRP4-Pep (P190-S226) at dosage of 5 mg/kg, 10 mg/kg, 20 mg/kg, respectively, once per day for six weeks. After euthanasia, the serum, distal femur, proximal tibia and vertebrae of mice in each group were harvested for analysis as described in Study 3.^9,10,28–31^ Meanwhile, the effect of LRP4-Pep on muscle was determined as described in Study 3.^32,33^

#### Study 5: Pharmacologically determining the effect of LRP4-Pep on bone formation in OVX mice

The 10-week-old female C57BL/6 J mice were ovariectomized (OVX) or sham-operated (SHAM), then left untreated for six weeks. At sixteen-week-old, OVX mice in OVX-Baseline group and SHAM mice in SHAM-Baseline group were sacrificed as baseline before treatment. The remaining OVX mice and SHAM mice were subcutaneously administrated with saline and exogenous LRP4-Pep (P190-S226, 10 mg/kg), respectively, once per day for four weeks (*n* = 6 per group). After euthanasia, the serum, distal femur, proximal tibia and vertebrae of mice in each group were harvested for analysis as described in Study 3.^9,10,28–31^

### Evaluation protocol

#### Surface plasmon resonance

Surface plasmon resonance (SPR) was performed on the Biacore 8 K+ system (Cytiva) and Biacore 8 K Control Software. Ligand protein was diluted in 10 mM sodium acetate pH 4.0 buffer (BR100349) and was immobilized on sensor chip CM5 (Cytiva, BR100530, Series S). Analyte protein was diluted in HBS-EP+ buffer (Cytiva, BR100669). Different concentrations of analyte were injected at a flow rate of 30 μL/min for 60 s. At the end of the sample injection, the same buffer was flowed over the sensor surface to facilitate dissociation. After a 3 min dissociation time, the sensor surface was regenerated by injecting regeneration solution (10 mmol/L glycine-HCI, pH 2.5; Cytiva, BR100356).^35^ Raw data was analyzed using Biacore Insight Evaluation Software. All *K*_*d*_ values were calculated after deducting the reference surface signal.

#### Plasmid construction, transfection and protein expression

The pCDNA 3.1-LRP4 was used as a template for the amplification of open reading frame by PCR to construct a series of wild-type (WT), truncated and mutated *Lrp4* plasmids (Tables S1&S2). Briefly, the empty vector was linearized with restriction endonuclease *EcoR* V and *Xho* I. After ligating the PCR product, the Flag-tagged DNA fragments and linear carrier with the one-step cloning kit (Vazyme, C112-01), further the ligation products were transformed into *E.coli* DH5α competent cells and incubated on the solid agar plates more than 12 h. Colony PCR and restriction enzyme digestion were used to pick up positive clones for subsequent plasmid amplification and extraction.^36^ Transfections were performed in the MC3T3-E1 cells with Lipomaster 3000 transfection Reagent (Vazyme, TL301) according to the manufacturer’s instructions.^37^

#### Pull-down assay

Cell lysates were collected using RIPA Lysis and Extraction Buffer (Thermo Scientific, 89901) supplemented with Protease Inhibitor Cocktail (CST, 5871) and centrifuged at 4 000 r/min for 30 min at 4 °C and replaced with binding buffer (PBS buffer, 20 mmol/L imidazole, pH 8.0). After pretreatment with binding buffer for three times, 20 μL Ni-NTA Magnetic Agarose Beads were incubated with 6 μg His-SOST and 6 μg His-loop3 peptide, respectively, in binding buffer for 2 h at 4 °C with gentle rotation. The beads carrying His-SOST and His-loop3 peptide were then incubated with 500 μL cell lysate supernatant at 4 °C overnight with gentle rotation. To eliminate the effects of non-specific binding between beads and cell lysate on targeted interaction identification, 20 μL beads and 500 μL cell lysate were incubated under the same conditions. After incubation, the beads were washed 3 times with 500 μL wash buffer (PBS buffer, 20 mmol/L imidazole, 0.005% Tween 20, pH 8.0), and finally eluted with elution buffer (PBS buffer, 250 mmol/L imidazole, 0.005% Tween 20, pH 8.0).^38^ Then, western blots were conducted with anti-His antibody (Abcam, ab245114, diluted at 1:1 000), anti-LRP4 antibody (Invitrogen, PA5-68218, diluted at 1:1 000), anti-LRP6 antibody (Creative Biolabs, CBYCL-470, diluted at 1:100), or anti-Flag antibody (Abcam, ab125243, diluted at 1:2 000).^39^

#### TOP-Wnt-induced luciferase reporter assay

The MC3T3-E1 cells were seeded in 24-well plates and co-transfected with the corresponding reporter plasmids including TOPFlash, sv40 and Wnt1, as well as WT *Lrp4* and mutated *Lrp4* plasmids, respectively, using Lipofectamine 3000 reagent (Thermo Scientific, L3000001). After 6-h incubation, the culture medium was changed to fresh DMEM medium containing recombinant sclerostin protein with or without LRP4-Pep. 24 h after treatment, the cells were lysed with 100 μL/well passive lysis buffer, subsequently 20 μL was taken for analysis. Luciferase assays were performed using the Dual-Luciferase Reporter Assay System (Promega, E1980) according to the manufacturer’s protocol.^40,41^

#### Lentivirus transduction and puromycin selection

WT*-Lrp4* and *Lrp4m* lentiviral particles were commercially purchased from Vector Builder Co. Ltd., Guangzhou, China. WT*-Lrp4* and *Lrp4m* stable overexpression was carried out according to the manufacturer’s protocol. Briefly, MC3T3-E1 cells were seeded at 5 × 10^5^ cells/well in a 6-well plate and incubated overnight. At 40% confluence, the culture medium was replaced with 1 mL fresh complete medium, and the amount of lentivirus (10^8^ Tu/mL) added is calculated at a multiplicity of infection (MOI) of 70 with the addition of 8 μg/mL polybrene (Sigma, TR-1003). 6 h later, 1 mL of fresh medium was added. After incubating the cultures for 72 h in a 37 °C/CO_2_ incubator, transfection rate was checked under fluorescence microscope. Stable WT*-Lrp4* and *Lrp4m* overexpressing cells were selected by adding 2.0 μg/mL puromycin (Thermo Scientific, A1113802), respectively. A plate of cells without lentiviral was used as a negative control to determine the time point at which the puromycin screen ended.

#### Real-time quantitative PCR

Real-time quantitative PCR was used to detect the expression of total mRNA, which was extracted at time points of 3, 7 or 21 days. Total RNA from cultured cells was isolated by homogenization using TRIzol (Invitrogen, 15596026) and reverse transcribed into cDNA using a HiScript III RT SuperMix kit (Vazyme, R323). Quantitative PCRs were performed on the QuantStudio 7 PRO System (Applied Biosystems). Bone formation biomarkers’ expression was measured using primers including those for GAPDH (F: AGGTCGGTGTGAACGGATTTG, R: GGGGTCGTTGATGGCAAC), β-catenin (F: GCCACAGGATTACAAGAAGCGG, R: GGCACCAATGTCCAGTCCAAG), ALP (F: AACCCAGACACAAGCATTCC, R: GCCTTTGAGGTTTTGGGTCA), and Bglap (OCN; F: CAGCCACCGAGACACCAT, R: CCAGCAGAGCGACACCCTA). Relative RNA expression of genes was determined using the 2^-ΔΔCt^ method with GAPDH as the endogenous normalizer.^42^

#### Osteogenic differentiation, ALP staining and Alizarin red staining

MC3T3-E1 cells were cultured with a density at 3 × 10^5^ cells per well in α-MEM for 12 h. After reaching 80% confluence, the medium was replaced by α-MEM medium with osteogenic components (10 mmol/L β-glycerophosphate, 0.1 μmol/L dexamethasone and 0.25 mmol/L ascorbate) for further culturing. The medium was changed every 48–72 h. After 7 days of osteogenic differentiation, alkaline phosphatase (ALP) staining and activity was performed with BCIP/NBT Alkaline Phosphatase Kit (Beyotime) with p-nitrophenyl phosphate as the chromogenic substrate, according to manufacturer’s instructions.^43–45^ After 21 days of osteogenic differentiation, Alizarin Red S solution (40 mmol/L, pH 4.2, Cyagen, ALIR-10001) was used to evaluate extracellular matrix mineralization in mature osteoblasts.^44^ Cells were then washed three times with distilled water and examined for the presence of calcium deposits. Then, the calcium-bound dye was removed with pH neutral 10% cetylpyridinium chloride in 10 mmol/L sodium phosphate buffer. Mineralization was quantified by a SpectraMax i3x Multi-Mode Microplate Reader (Molecular devices) at 550 nm wavelength.^46,47^

#### Immunofluorescence analysis (IF)

MC3T3-E1 cells were cultured with a density of 1 × 10^5^ cells per well in α-MEM at the confocal dish (Thermo, 150680) for 12 h. After being fixed with 4% PFA, the cells were incubated with anti-LRP6 antibody (Creative Biolabs, CBYCL-470, 1:100) and anti-sclerostin antibody (Thermo, PA5-113315, 1:100) overnight at 4 °C, followed by incubation with goat anti-mouse IgG H&L (Alexa Fluor 594, 5 μg/mL) and goat anti-rabbit IgG H&L (Alexa Fluor 488, 5 μg/mL) for 2 h at 37 °C. Hoechst was used to stain the cell nuclei. The confocal microscope (Leica, STELLARIS STED) was utilized to obtain fluorescent images.^48^

#### Complex structure modeling

Protein sequences for murine SOST (Q99P68), LRP6 (O88572), and LRP4 (Q8VI56) were obtained from UniProt.^49^ Based on the structural information from PDB entry 6L6R, the SOST sequence corresponding to residues 76-175 was utilized, with residues 20-630 and the extracellular domain (residues 21-1725) utilized for LRP6 and LRP4, respectively. A mutant form of LRP4 was generated by substituting residues 200, 201, 208, 209, and 210 with alanine. The protein complexes, including SOST-LRP6, wild-type SOST-LRP6-LRP4, and mutant SOST-LRP6-LRP4, were predicted utilizing AlphaFold3.^50^ ChimeraX was employed for structural visualization and interaction interface analysis of the predicted complexes.^51^

#### Molecular dynamics simulation

This study revealed the complexity of protein structural dynamics using state-of-the-art molecular dynamics simulations with GROMACS 2023.5.^52^ Initial complex structures were placed in a cubic box, solvated with the OPC water model,^53^ and neutralized with ions. The AMBER99SB-ILDN force field^54^ was employed. After energy minimization, the system underwent NVT and NPT equilibrations (100 ps each) at 300 K. Production runs were performed for 20 ns in the NPT ensemble (300 K, 1 bar) using a 2-fs time step. Trajectory analysis focused on the analysis of structural dynamics, employing the MDAnalysis package^55^ along with custom Python scripts to compute center of mass distances and root mean square fluctuations (RMSF). Data visualization for these analyses was carried out using Matplotlib.^56^

#### Mice and genotyping

The *SOST*^*ki*^ mice,^9^ the *Lrp4m* mice (encoding LRP4-Y200A, G201A, Y208A, H209A, C210A), the *sost*^*−/−*^ mice and the Bglap-Cre mice were constructed in collaboration with GemPharmatech Co., Ltd, China (Figs. S4 & S5). Mouse genotypes were determined by PCR on tail genomic DNA. The *Lrp4m* mice was genotyped using DNA and amplified using FP 5ʹ- TTGGGTGGACAGGCATCAATG -3ʹ and RP 5ʹ- CCTCCCTGAATAACTTCGTATAATGTATGC -3ʹ to generate 0 bp (wild-type) or 1 567 bp (targeted) amplicons, FP 5ʹ- GATCCCCATCAAGCTGATAACATACG 3ʹ and RP 5’- GACACTCACGGCAGCTTTCCTC -3ʹ to generate 0 bp (wild-type) or 1 929 bp (targeted) amplicons, FP 5ʹ- GGGAACGAAGCTACAACCTGGAC -3ʹ and RP 5’- CGACAGTCCTGCTCATCAGAGTC -3ʹ to generate 835 bp. The *sost*^*−/−*^ allele was genotyped using DNA and amplified using FP 5ʹ-AGTGATATGGTGAGGCTGGATGC-3ʹ and RP 5ʹ- GAACCTCAGTGATGGCTTAGTGG-3ʹ to generate 6 760 bp (wild-type) or 555 bp (homozygous) amplicons, FP 5ʹ-ACACACAATGTCTCGCCACTGT-3ʹ and RP 5’-CAGCTAACTGAAGAGACAGGGATAG-3ʹ to generate 378 bp (wild-type) or 0 bp (homozygous) amplicons. The Bglap-Cre mice was genotyped using DNA and amplified using FP 5ʹ-GGGCAGTCTGGTACTTCCAAGCT-3ʹ and RP 5ʹ-ATTGTGGTGCAGCCAAGCTGCTA-3ʹ to generate 0 bp (wild-type) or 304 bp (targeted) amplicons, FP 5ʹ-AGTCTTTCCCTTGCCTCTGCT-3ʹ and RP 5’- GGGTCTTCCACCTTTCTTCAG -3ʹ to generate 825 bp (wild-type) or 0 bp (targeted) amplicons.

#### Systemic delivery of *rAAV9* vectors

Considering the short half-life and ineffectiveness of exogenous recombinant sclerostin protein, we used the *recombinant adeno-associated virus, serotype 9 (rAAV9)* to express sclerostin in mice.^57,58^ The *rAAV9* carrying wild-type *sost* vectors were purchased from Vector Builder Co. Ltd., Guangzhou, China. The 6-month-old male *sost*^*−/*−^ and *sost*^*−/−*^*.Lrp4m* mice were intravenously injected with either *rAAV9-SOST* (5 × 10^11^ vg/mouse) or blank *rAAV9* vector as vehicle (veh), respectively, according to the manufacturer’s protocol.^57,58^

#### Synthesis and modification of LRP4-Pep peptide

LRP4-Pep peptide (P190-S226, PCNLEEFQCAYGRCILDIYHCDGDDDCGDWSDESDCS) was synthesized and purchased from GL Biochem Ltd., Shanghai, China. The peptide was end-protected by changing the front and back three amino acids from L-amino acids to D-configuration amino acids to improve hydrolytic stability.^59^ The LRP4-Pep peptide was dissolved in PBS at a storage concentration of 1 mmol/L for the following study.^57^

#### High performance liquid chromatography (HPLC)

The HPLC system was equipped with a KR100-5-C18-4.6×250 column (Kromasil, K08670357) to quantify the modified LRP4-Pep in plasma samples. The mobile phase elution gradient of phase A (0.1% Trifluoroacetic in 100% Acetonitrile) and phase B (0.1% Trifluoroacetic in 100% Water) was used. The flow rate, wavelength and volume were 1.0 mL/min, 220 nm and 10 µL, respectively. Standards were prepared in blank mice plasma containing sodium heparin with different concentrations of the modified LRP4-Pep.

#### Micro-CT analysis

*Micro-CT* analysis was used for analysis of the trabecular/cortical bone mass and bone architecture. The collected right femur, right tibia, and the fourth vertebrae (Lv4) were fixed with 4% PFA and scanned using a *Micro-CT* (version 6.5, vivaCT40, SCANCO Medical AG). Briefly, a total of 424 slices with a voxel size of 10 µm were scanned at the region of the proximal tibia/distal femur beginning at the growth plate and extending distally along the tibial/femoral diaphysis, the entire region of secondary spongiosa between proximal and distal aspects from the Lv4. Regions of interest (ROIs) were defined using Scanco evaluation software. Images of femurs, tibias and vertebrae were reconstructed and segmented (200 ms integration time, 0.8 sigma, 1 support, 260 thresholds). For the proximal tibia/distal femur 100 sequential slices beginning at 0.1 mm from the most proximal aspect of the growth plate in which both condyles were no longer visible were selected for analysis. The trabeculae were analyzed by manual contouring excluding the cortical bone. For Lv4, a central region was selected, beginning at the distal growth plate and extending proximally along the vertebral body. The freehand trabeculae ROI on 100 sequential slices was drawn to ensure that it was within the endosteal envelope. Trabecular bone parameters, including trabecular volume per total volume (Tb.BV/TV), trabecular volumetric bone mineral density (Tb.BMD), trabecular thickness (Tb.Th) and trabecular spacing (Tb.Sp) were calculated. For the femoral mid-shaft, 100 slices were measured at the exact centre and at the distal 50% of femur length using the automated thresholding algorithm. Trabeculae in contact with cortical bone were manually removed from the ROI. Cortical bone parameters, including cortical bone periosteal perimeter (Ct.PP) and cortical thickness (Ct. Th) were calculated.^9,10^

#### Bone histomorphometric analysis

Calcein (20 μg/g body weight, Sigma, C0875) was injected intraperitoneally at 10 and 2 days prior to euthanasia. The collected undecalcified right femur samples were dehydrated in increasing concentrations of sucrose (10%, 20%, 30% in PBS) for 24 h at each concentration and embedded in an optimal cutting temperature O.C.T. compound (Sakura Finetek). Frontal sections (thickness: 7 μm) of trabecular bone were obtained from the femoral metaphysis by longitudinal cryosection with CryoStar NX50 (Thermo Fisher Scientific). Cross sections (thickness: 7 μm) of cortical bone were obtained from the femoral mid-shaft. Fluorescence micrographs of the bone sections with calcein green labels were captured by a Q500MC fluorescence microscope (Leica). Hematoxylin and eosin (H&E) staining for bone surface and TRAP staining for multinuclear osteoclasts were performed. Dynamic parameters including the bone formation rate (BFR/BS) and mineral apposition rate (MAR), as well as static parameters including osteoclast surface/bone surface (Oc.S/BS) were analyzed and calculated according to the ASBMR standardized nomenclature for bone histomorphometry.^10,30,60^

#### Bone mechanical test

The left femurs and the fifth lumbar vertebrae (Lv5) of the mice were directly wrapped in PBS-soaked gauze and stored at −80 °C after sacrifice. Bones were thawed to room temperature and the compression test/three-point test were carried out by a universal testing machine (H25KS Series, Hounsfield Test Equipment Ltd) with a 2.5 kN load cell. For the three-point test, femurs were loaded in the anterior-posterior direction with the span length set as 17 mm. Load was applied with a constant displacement rate of 1 mm/min at the femur mid-shaft. After failure, the load vs. displacement curves were recorded, and the ultimate force (N), stiffness and fracture energy (J) were calculated for statistical analysis. For the compression test, Lv5s were isolated from vertebral columns and constructed into a cylinder with two parallel planes (5–7 cm) before the test. The Lv5s were positioned horizontally to the base. Load was applied constantly with a displacement rate of 1 mm/min. After failure, the load vs. displacement curves were recorded, and the failure force (N) and failure strength (MPa) were calculated for statistical analysis.^9,10,31^

#### Measurement of serum sclerostin, bone formation markers and bone resorption markers

Fasting blood samples of each group were obtained before sacrifice and serum was collected. The serum levels of sclerostin were detected using the sclerostin ELISA kits (R&D, DSST00; SEC864Mu, Cloud-Clone) in triplicate following manufacturer’s instruction.^9^ The serum levels of procollagen type 1 N-terminal pro-peptide (P1NP), osteocalcin (OCN) and β-CrossLaps of type 1 collagen containing cross-linked C-telopeptide (β-CTX) were evaluated using the mouse P1NP ELISA Kit (Sanggon Biotech, D721053), mouse osteocalcin ELISA Kit (Abcam, ab285236) and mouse β-CTX ELISA Kit (Jianglaibio, JL31101) according to the manufacturer’s instructions.^28,29^

#### Measurement of maximal forelimb grip strength

Grip strength was assessed with a computerized grip strength meter (Shanghai xinruan, Model XR501). The apparatus consisted of a rectangular-shaped metal bar connected to a force transducer. For each trial, mice were placed on the device and pulled parallel to the ground until loss of grip. The peak force of each measurement was automatically recorded in grams (G) by the device. The forelimb grip strength in each mouse was measured in triplicate with 10 s of rest between trials.^33^

#### Measurement of muscle wight and muscle size

Following dissection, gastrocnemius muscles were weighed. The gastrocnemius muscle-to-body weight ratio (M/B ratio) was calculated. Subsequently, the muscles were cryo-sectioned at 10 μm thickness. Transverse sections were stained with H&E to visualize muscle fibers (stained pink) and connective tissue (stained blue). Mean muscle fiber cross-sectional area (CSA) was quantified from mid-belly regions using ImageJ software.^32^

#### Statistical analysis

Comparisons between groups were carried out using the unpaired Student’s *t* test or One-Way ANOVA with Tukey’s post-hoc test. Error bars indicated the mean ± standard deviation. All statistical data were analyzed by GraphPad Prism (version 8; GraphPad Software), and *P* < 0.05 was considered statistically significant.

## Supplementary information

Supplementary information
