# Supplementary material for: Osteoblastic sclerostin loop3-LRP4 interaction required by sclerostin to inhibit bone formation
Source: Bone Res. 2026 Apr 24;14:45. doi: 10.1038/s41413-026-00511-x (PMC13109412; doi:10.1038/s41413-026-00511-x)
Supplement: Supplementary file 1 — Supplementary information [file 41413_2026_511_MOESM1_ESM.docx]

**Supplementary Materials for**

**Osteoblastic sclerostin loop3-LRP4 interaction required by sclerostin to inhibit bone formation**

Luyao Wang^1,2,3#^*, Xiaohui Tao^1,2,3#^, Hewen Jiang^4#^, Shijian Ding^1,2,3^, Ning Zhang^4^, Xin Yang^1,2,3^, Shenghang Wang^1,2,3^, Yihao Zhang^1,2,3^, Nanxi Li^1,2,3^, Haitian Li^1,2,3^, Zhanghao Li^1,2,3^, Xiaoxin Wen^1,2,3^, Meiheng Sun^1,2,3^, Chuanxin Zhong^1,2,3^, Heiwa So^1,2,3^, Jin Liu^1,2,3^, Yuanyuan Yu^1,2,3^, Hua Yue^5^, Xianghang Luo^6^, Péter Ferdinandy^7^, Tao Zhang^8^, Shu Zhang^9^, Zhenlin Zhang^5^, Aiping Lu^1,2,3^, Baoting Zhang^4^*, Ge Zhang^1,2,3^*

^#^ Luyao Wang, Xiaohui Tao and Hewen Jiang contributed equally to this work.

* Correspondence and requests for materials could be addressed to Ge Zhang ([zhangge@hkbu.edu.hk](mailto:zhangge@hkbu.edu.hk)), Luyao Wang ([luyaowang@hkbu.edu.hk)](mailto:luyaowang@hkbu.edu.hk)，) and Baoting Zhang ([zhangbaoting@cuhk.edu.hk](mailto:zhangbaoting@cuhk.edu.hk)).

**This PDF file includes:**

**Fig. S1.** Binding analysis for the interaction between sclerostin and LRP4

**Fig. S2.** Characterization and validation of *Lrp4m* and LRP4-Pep for blockade of the interaction between sclerostin loop3 and LRP4

**Fig. S3.** The binding of sclerostin to LRP6 within osteoblasts overexpressing wild-type LRP4 (WT-LRP4) or LRP4m *in vitro*

**Fig. S4.** Blockade of sclerostin loop3-LRP4 interaction diminished the antagonistic effects of sclerostin on Wnt/β-catenin signaling and osteogenic potential in osteoblasts *in vitro*

**Fig. S5.** Construction and sequencing of *Lrp4m* mice

**Fig. S6.** Genotyping of *Lrp4m* mouse model, *Lrp4m/OB-Lrp4* mouse model, *sost^-/-^* mouse model, *sost^-/-^.Lrp4m* mouse model and *SOST^ki^* mouse model

**Fig. S7.** The bone phenotypes of *Lrp4m* mice, *Lrp4m/OB-Lrp4* mice and WT littermates

**Fig. S8.** The bone resorption parameters of *Lrp4m* mice and WT littermates

**Fig. S9.** The effect of *Lrp4m* and LRP4-Pep on muscle in mice

**Fig. S10.** The bone formation of *sost^-/-^* mice and *sost^-/-^.Lrp4m* mice, with and without *rAAV9*-mediated re-expression of sclerostin

**Fig. S11.** Determination of the administration dosage, interval and duration of LRP4-Pep *in vivo*

**Fig. S12.** The influence of the exogenous LRP4-Pep in the antagonistic effect of sclerostin on bone formation in *SOST^ki^* mice

**Fig. S13.** The effect of the exogenous LRP4-Pep on bone formation in OVX mice

**Fig. S14.** Molecular dynamics simulation of interaction between sclerostin and wild-type/mutant LRP4s, with or without LRP6

**Fig. S15.** The binding pocket within the LA5 domain of LRP4, identified by the DoGSiteScorer server, is highlighted in red

**Table S1.** Full-length and truncated LRP4 ([Q8VI56](https://www.uniprot.org/uniprotkb/Q8VI56/entry)) containing different domains

**Table S2.** Sequence of LA5 domain of wild-type and mutated LRP4


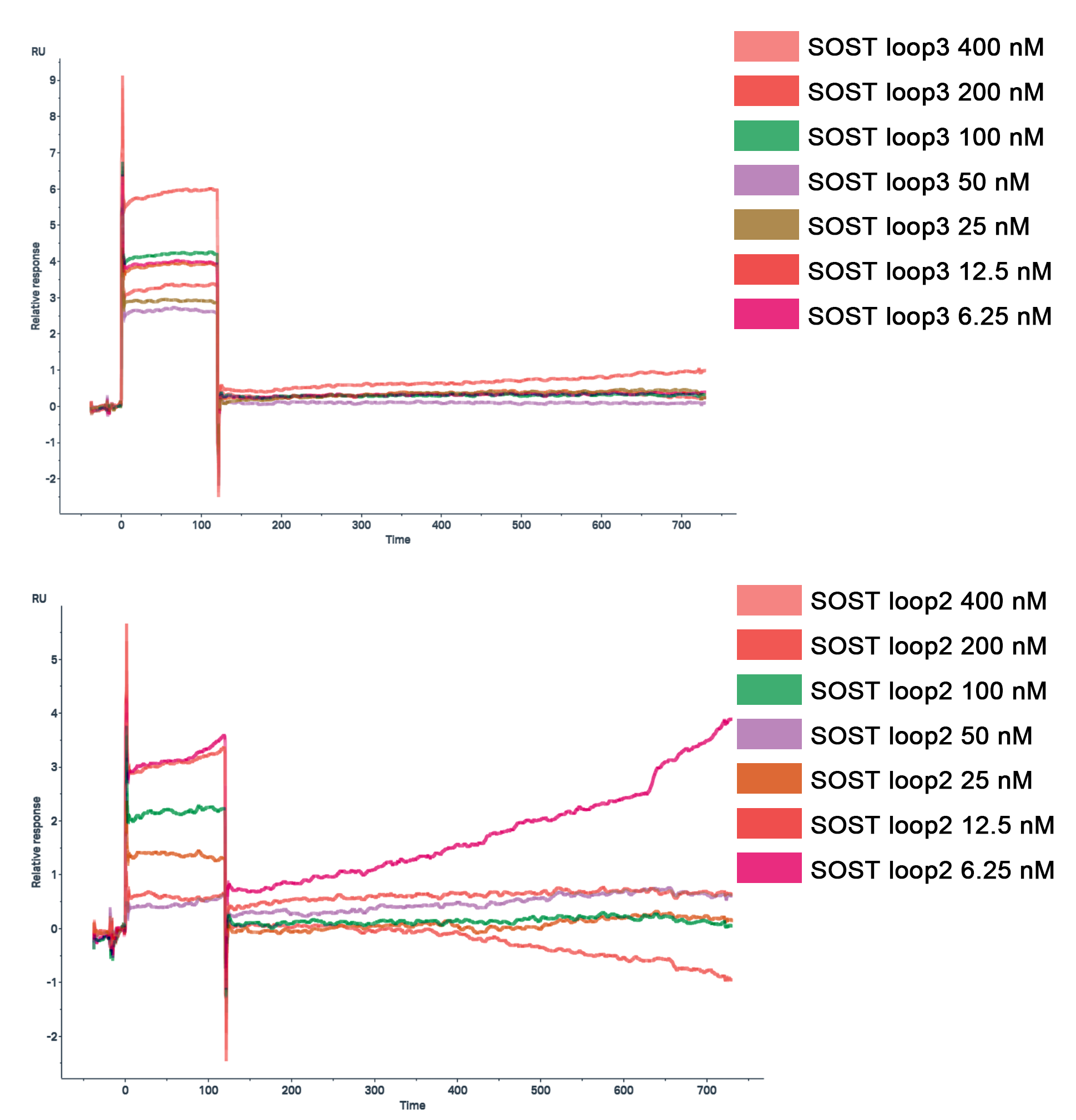


**LRP4 - SOST loop2, No Binding**

**a**


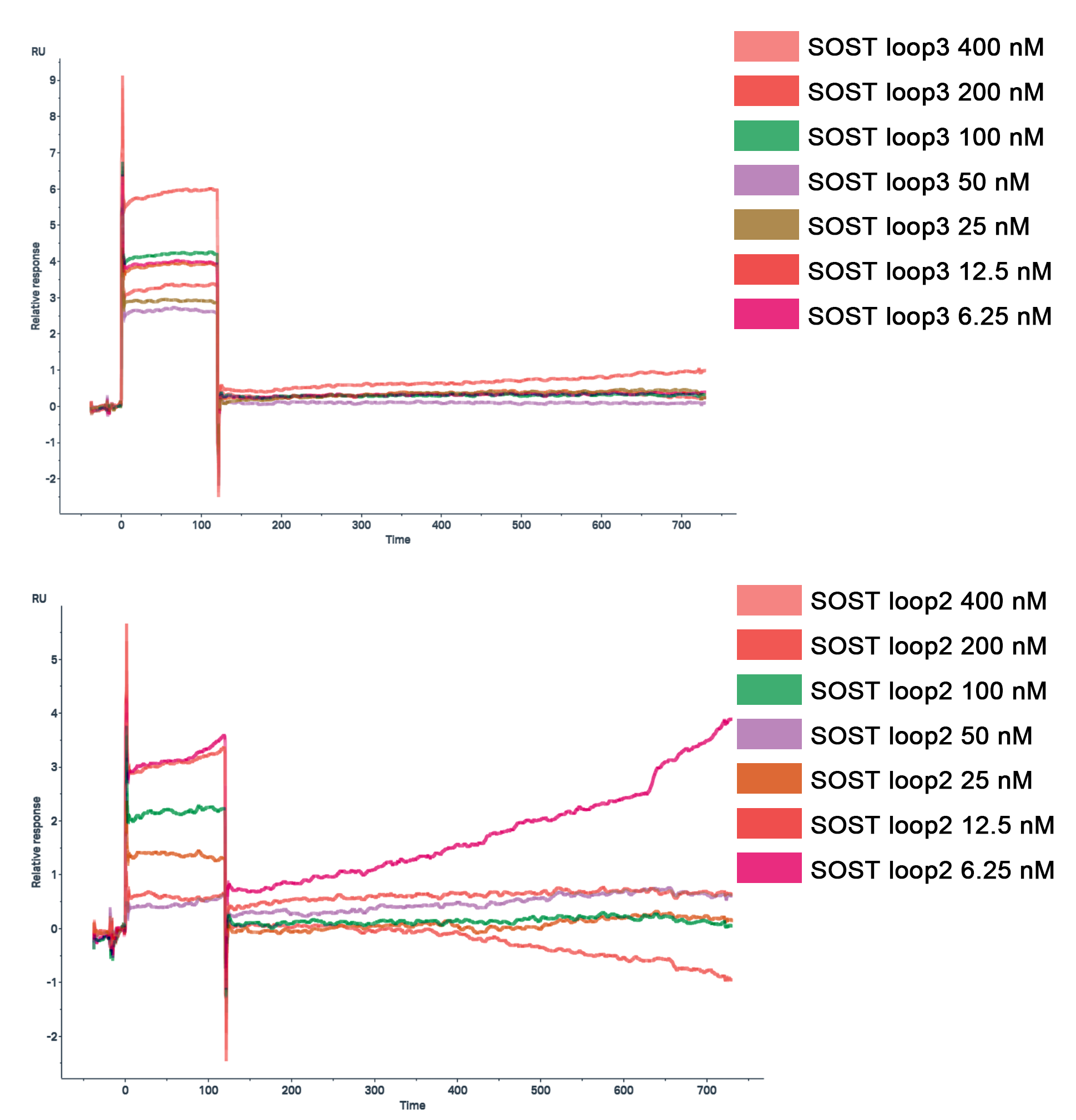


**LRP6 - SOST loop3, No Binding**


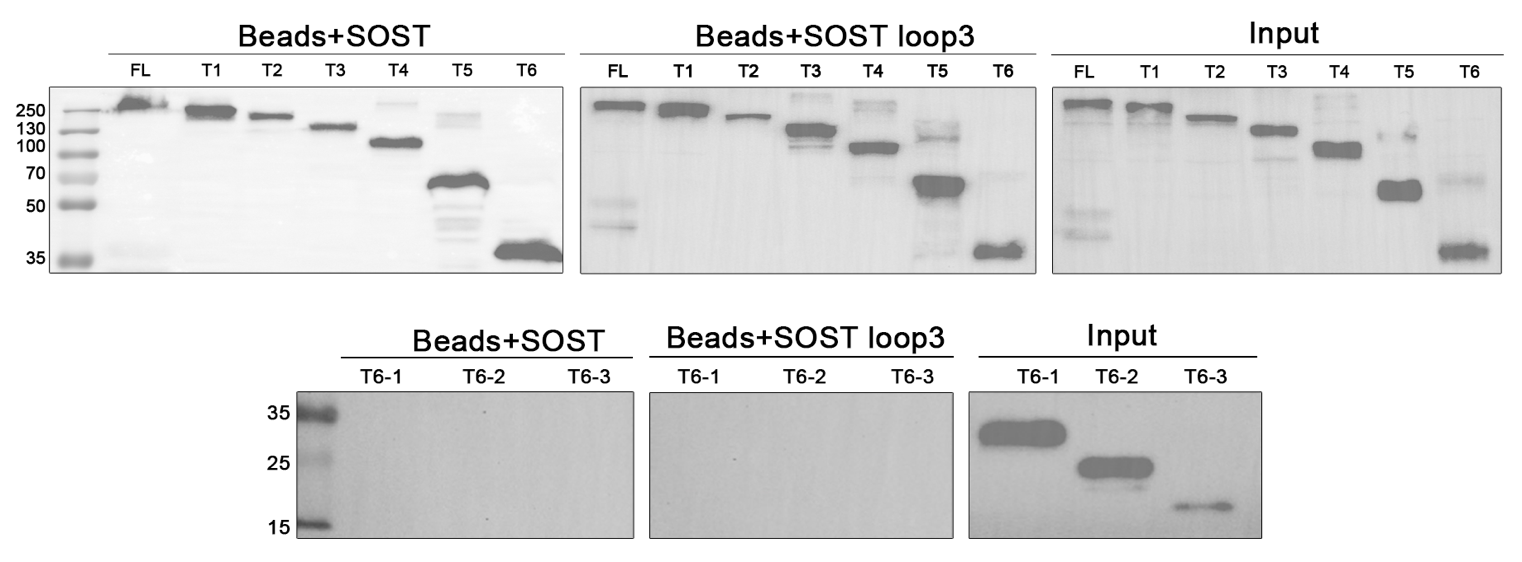


**b**

**
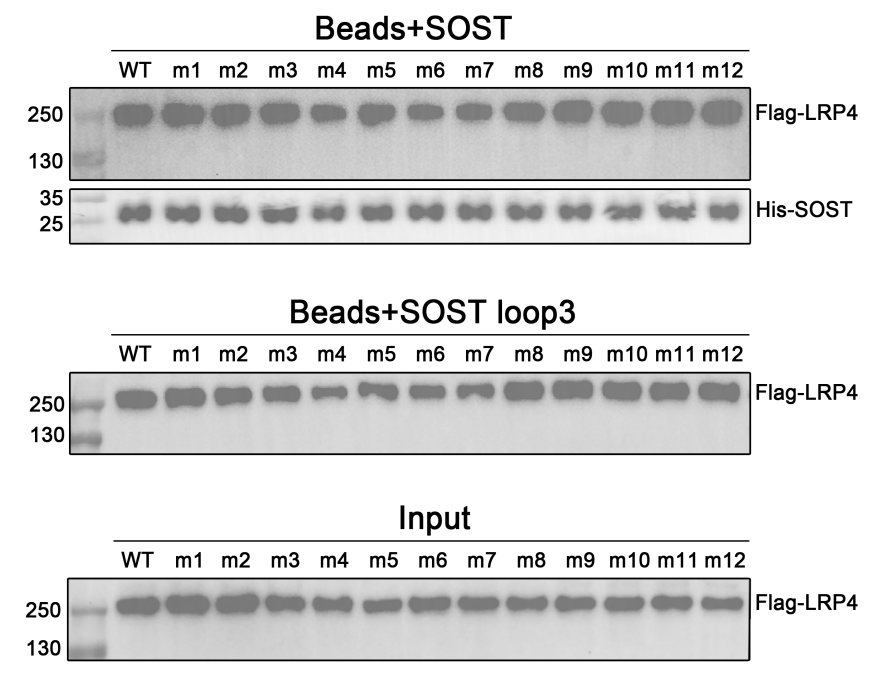
**

**Sclerostin - LRP4-LA5, *K_d_* = 14.3 nM**

**Sclerostin loop3 - LRP4-LA5, *K_d_* = 13.5 nM**


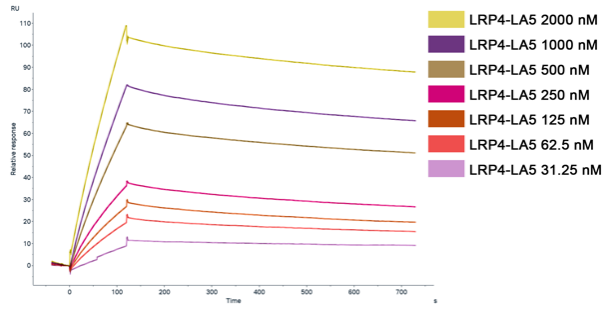

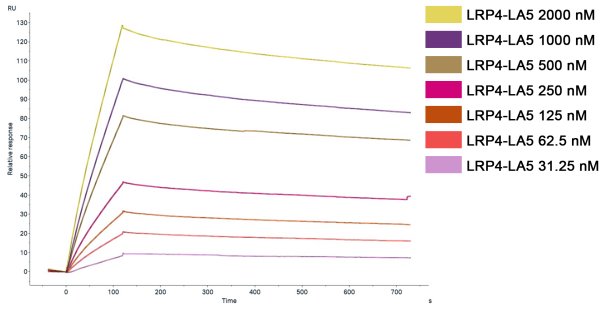


**c**

**d**

**Fig. S1. Binding analysis for the interaction between sclerostin and LRP4. (a)** The binding affinity of LRP6 to sclerostin loop3 (left), and LRP4 to sclerostin loop2 (right), respectively, by SPR analysis. **(b)** Binding analysis for the interaction of sclerostin/sclerostin loop3 to full-length and truncated LRP4 (upper: LRP4-FL, LRP4-T1, LRP4-T2, LRP4-T3, LRP4-T4, LRP4-T5 and LRP4-T6, lower: LRP4-FL, LRP4-T6-1, LRP4-T6-2 and LRP4-T6-3). **(c)** The binding affinity of sclerostin (upper) and sclerostin loop3 (lower) to LRP4-LA5 fragment by SPR analysis, respectively. **(d)** Binding analysis for the interaction of sclerostin (SOST)/sclerostin loop3 (SOST loop3) to wild-type and LRP4 muteins (WT-LRP4, LRP4-m1, LRP4-m2, LRP4-m3, LRP4-m4, LRP4-m5, LRP4-m6, LRP4-m7, LRP4-m8, LRP4-m9, LRP4-m10, LRP4-m11 and LRP4-m12).

**a**

**b**


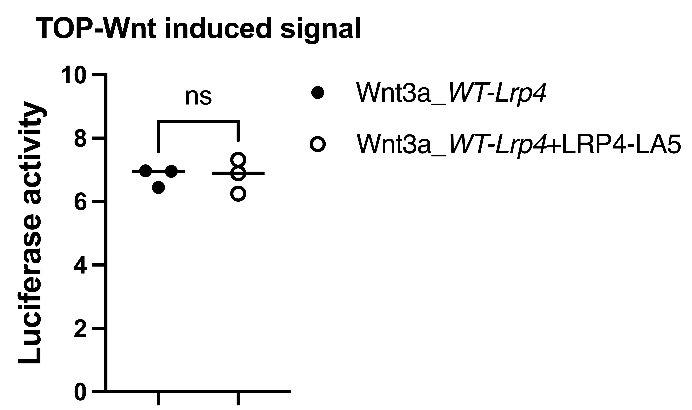

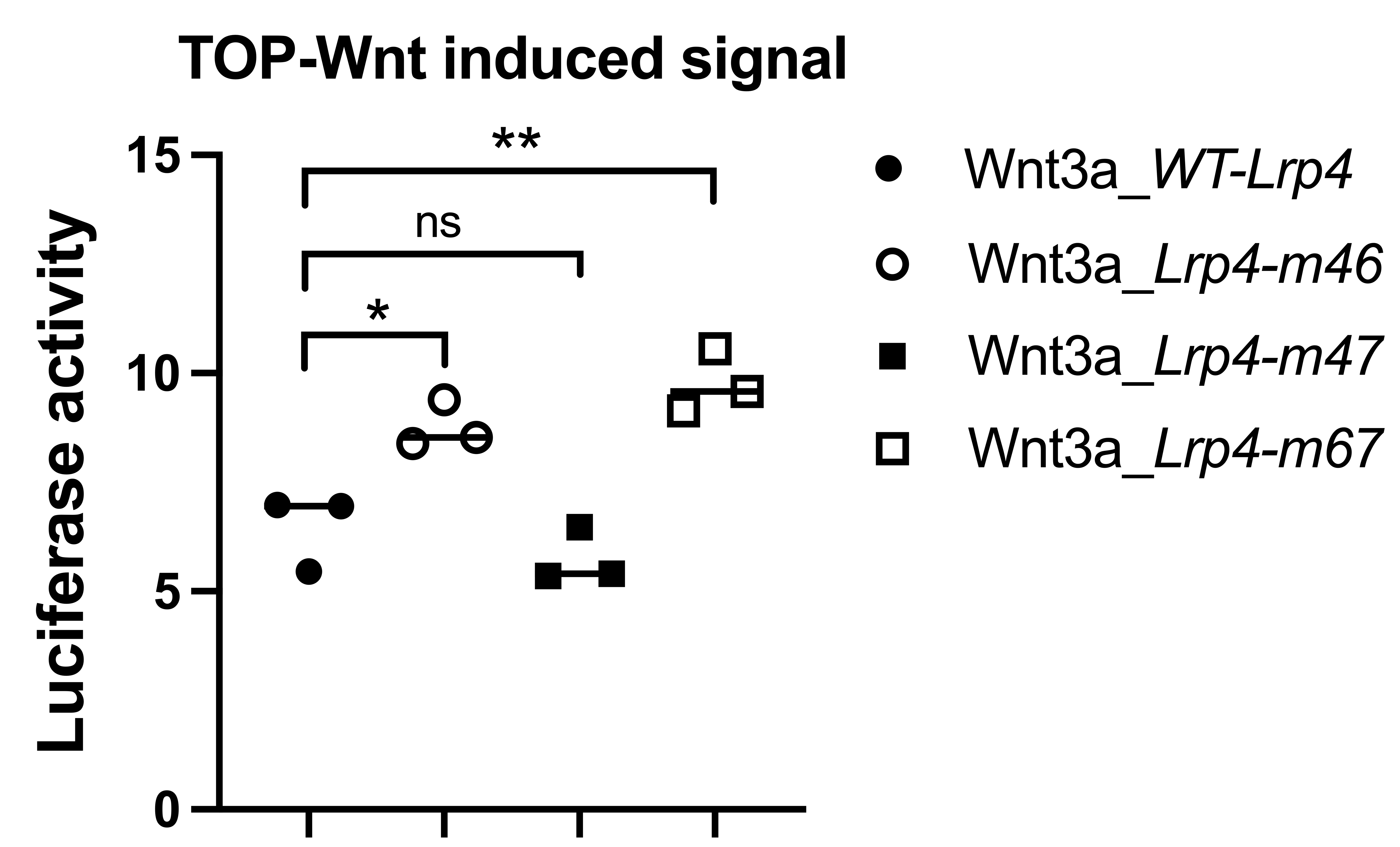


**Fig. S2. Characterization and validation of *Lrp4m* and LRP4-Pep for blockade of the interaction between sclerostin loop3 and LRP4. (a)** The effect of *Lrp4-m46* (encoding LRP4-Y200A, G201A, L205A, D206A, I207A), *Lrp4-m47* (encoding LRP4-Y200A, G201A, Y208A, H209A, C210A) and *Lrp4-m67* (encoding LRP4-L205A, D206A, I207A, Y208A, H209A, C210A) on Wnt/β-catenin signaling in osteoblasts (MC3T3-E1 cells), in the absence of sclerostin. **(b)** The effect of LRP4-LA5 peptide (P190-S226, named as LRP4-Pep) on Wnt/β-catenin signaling in MC3T3-E1 cells, in the absence of sclerostin. The unpaired t-test was used to determine the intergroup differences. ns: *P* > 0.05; * *P* < 0.05; ** *P* < 0.01.


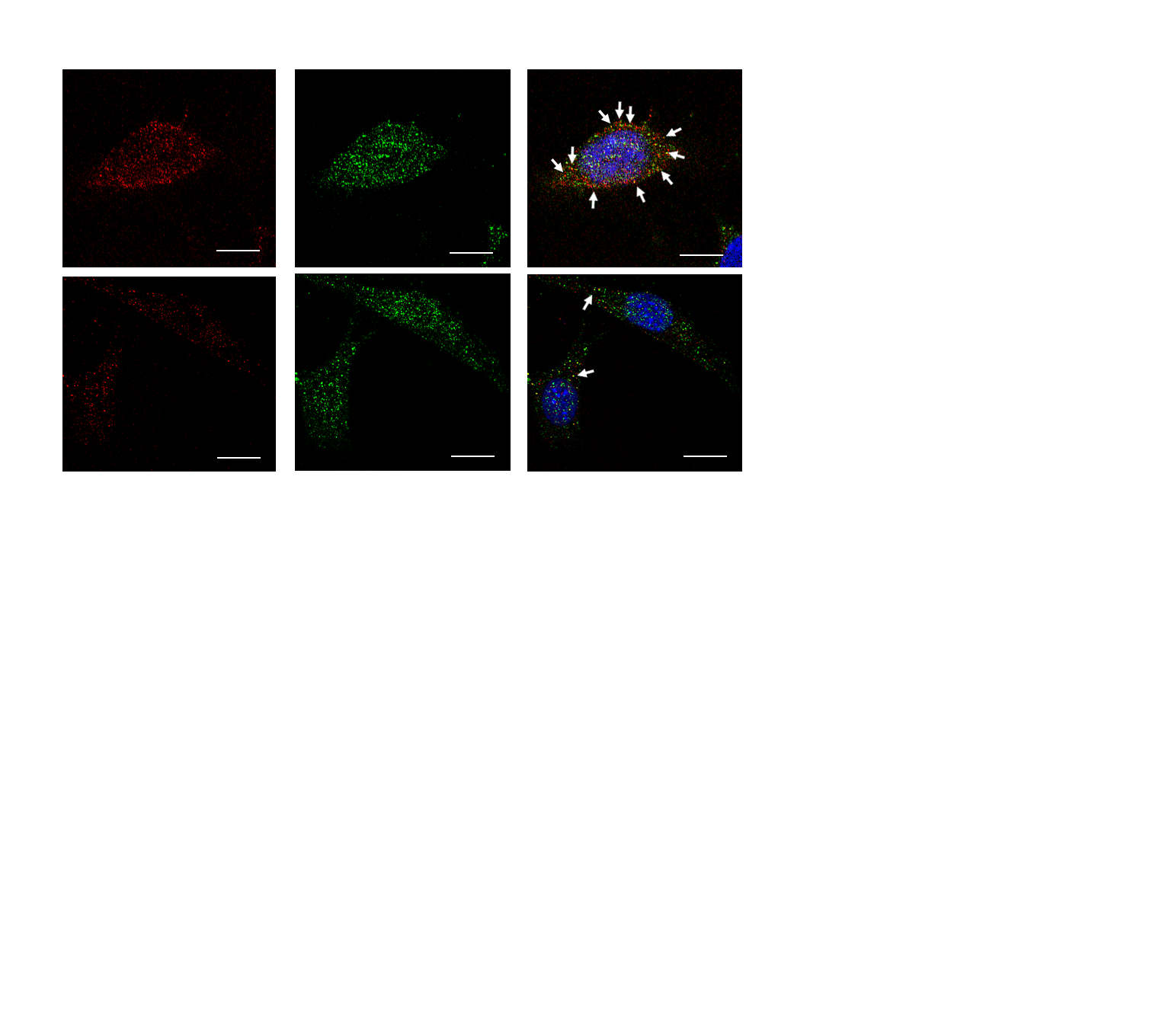


LRP4m

WT-LRP4

Sclerostin

LRP6

Merge

**Fig. S3. The binding of sclerostin to LRP6 within osteoblasts overexpressing wild-type LRP4 (WT-LRP4) or LRP4m *in vitro*.** Confocal microscopy analysis of MC3T3-E1 cells transfected with plasmids encoding WT-LRP4 (upper panel) and LRP4m (lower panel). The MC3T3-E1 cells were probed for sclerostin (Red fluorescence) and LRP6 (Green fluorescence). Arrows indicated the co-location of sclerostin and LRP6. Scale bars, 10 μm.


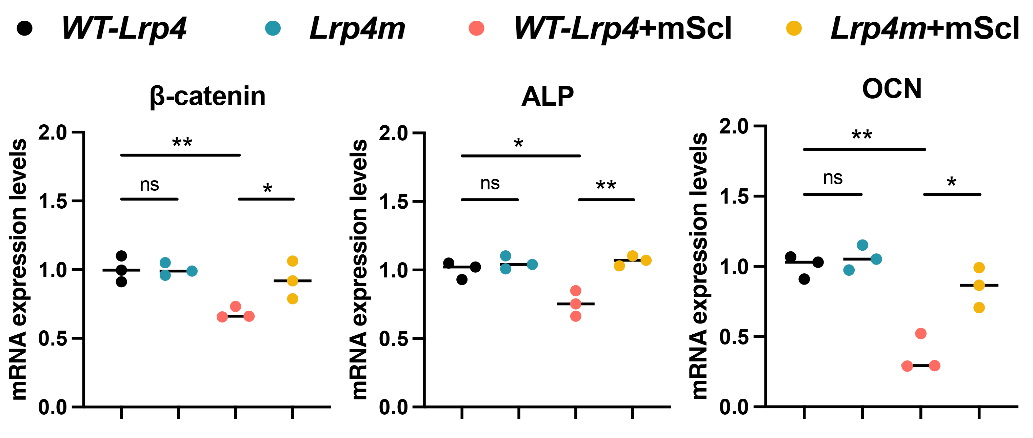


**a**

**b**


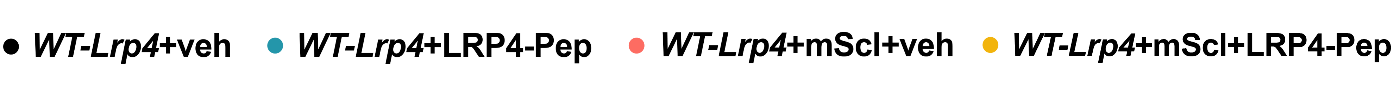

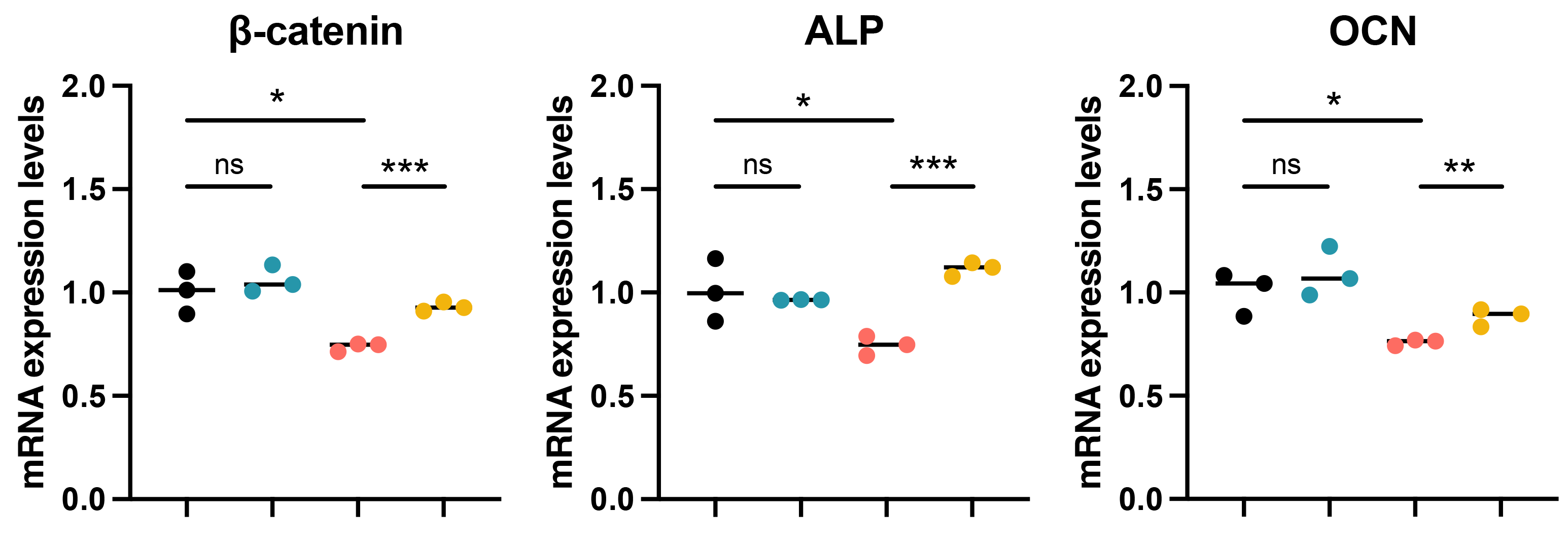


**Fig. S4. Blockade of sclerostin loop3-LRP4 interaction diminished the antagonistic effects of sclerostin on Wnt/β-catenin signaling and osteogenic potential in osteoblasts *in vitro*. (a)** The influence of *Lrp4m* (encoding LRP4-Y200A, G201A, Y208A, H209A, C210A) in the antagonistic effects of sclerostin on the mRNA expression of β-catenin (left), ALP (middle) and OCN (right) in MC3T3-E1 cells. **(b)** The influence of LRP4-Pep (LA5, P190-S226, PCNLEEFQCAYGRCILDIYHCDGDDDCGDWSDESDCS) in the antagonistic effects of sclerostin on the mRNA expression of β-catenin (left), ALP (middle) and OCN (right) in MC3T3-E1 cells. The unpaired t-test was used to determine the intergroup differences. ^ns^*P* > 0.05; **P* < 0.05; ***P* < 0.01; ****P* < 0.001; *****P* < 0.0001.


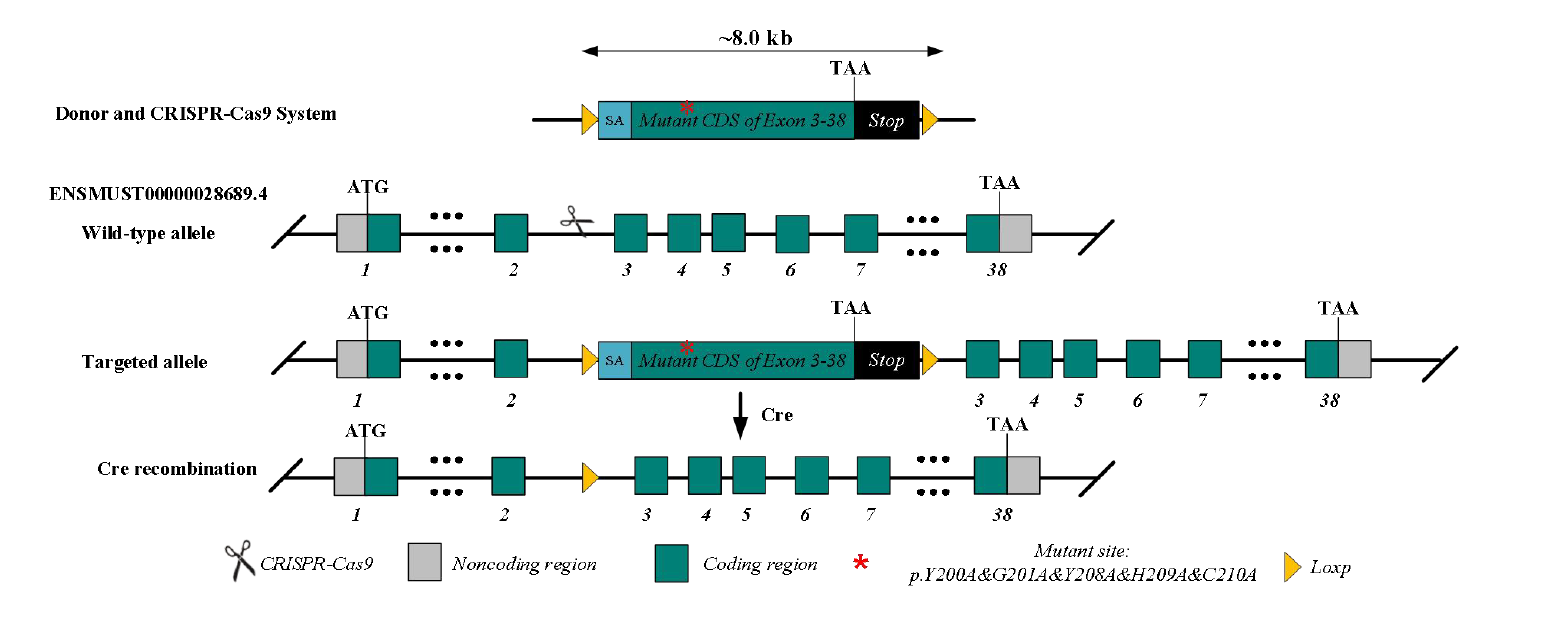


**a**

**b**


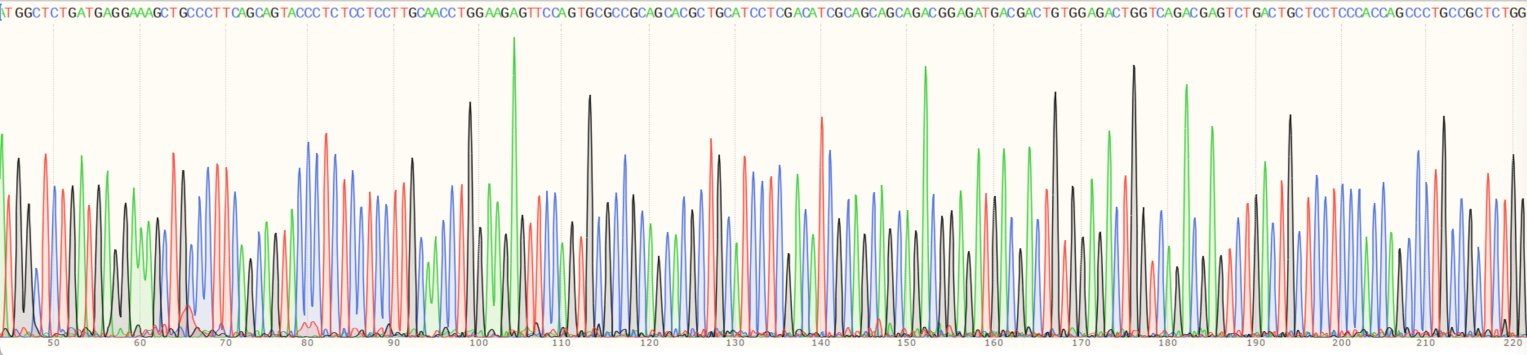


**Y208A, H209A, C210A**

**Y200A, G201A**

**Fig. S5. Construction and sequencing of *Lrp4m* mice. (a)** Construction of the *Lrp4m* mice. **(b)** DNA-sequencing of *Lrp4m* mice. Red highlights indicated the mutant base sequences within *Lrp4* ([Q8VI56](https://www.uniprot.org/uniprotkb/Q8VI56/entry)).

**
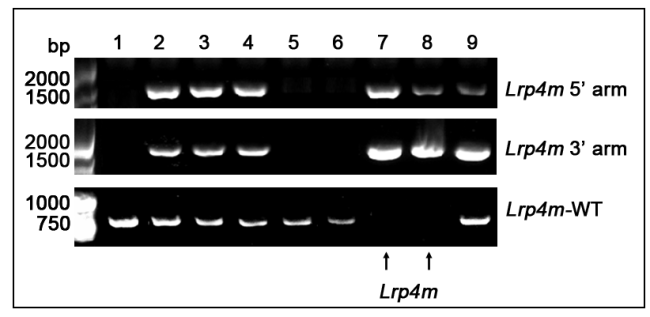

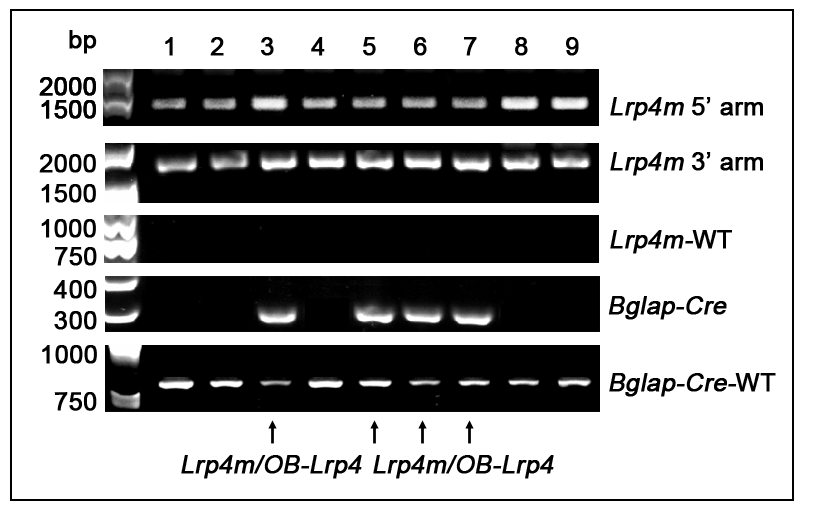

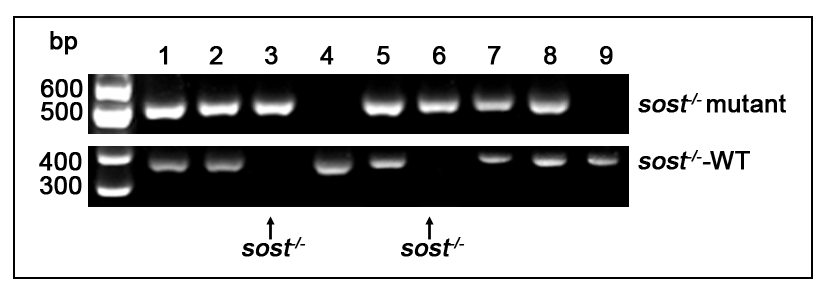

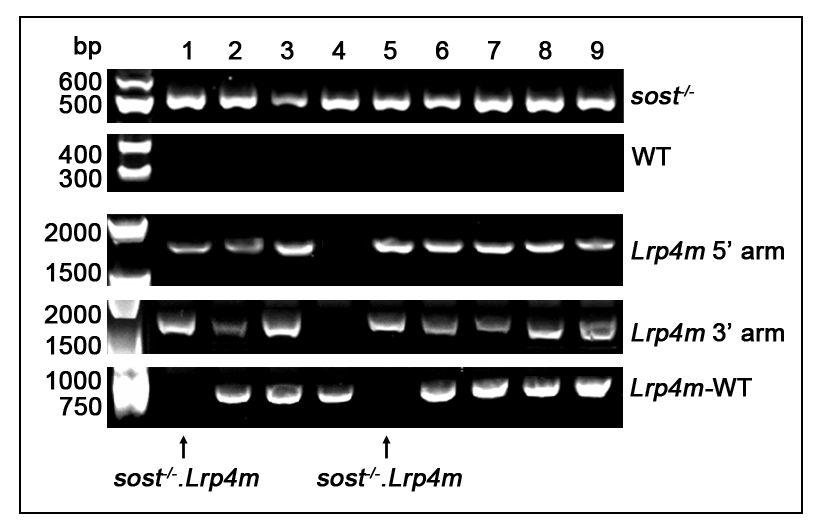

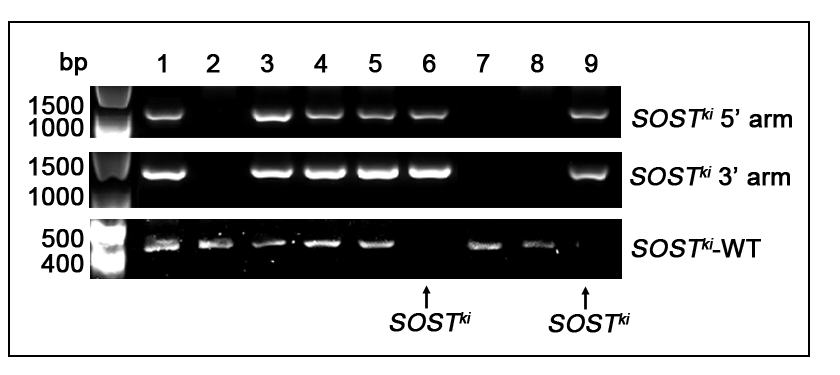
**

**b**

**a**

**c**

**d**

**e**

**Fig. S6. Genotyping of *Lrp4m* mouse model, *Lrp4m/OB-Lrp4* mouse model, *sost^-/-^* mouse model, *sost^-/-^.Lrp4m* mouse model and *SOST^ki^* mouse model. (a)** Wells 7 and 8 represented the PCR amplification products resolved by agarose gel electrophoresis from *Lrp4m* mice. **(b)** Wells 3, 5, 6 and 7 represented the PCR amplification products resolved by agarose gel electrophoresis from *Lrp4m/OB-Lrp4* mice. **(c)** Wells 3 and 6 represented the PCR amplification products resolved by agarose gel electrophoresis from *sost^-/-^* mice. **(d)** Wells 1 and 5 represented the PCR amplification products resolved by agarose gel electrophoresis from *sost^-/-^.Lrp4m* mice. **(e)** Wells 6 and 9 represented the PCR amplification products resolved by agarose gel electrophoresis from *SOST^ki^* mice.


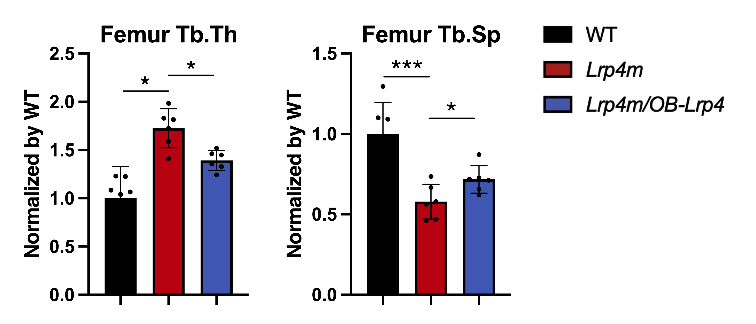


**b**

**a**

**Distal femur**

**
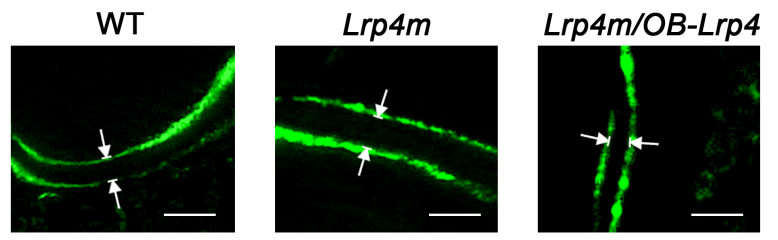
**

**d**


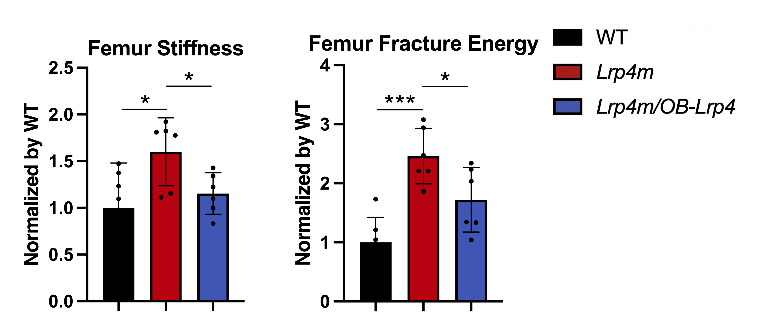


**c**

**Femoral mid-shaft**

**
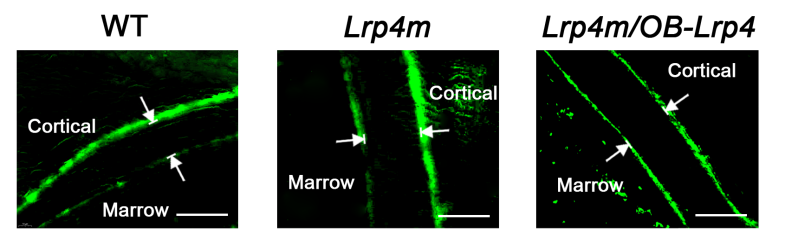
**


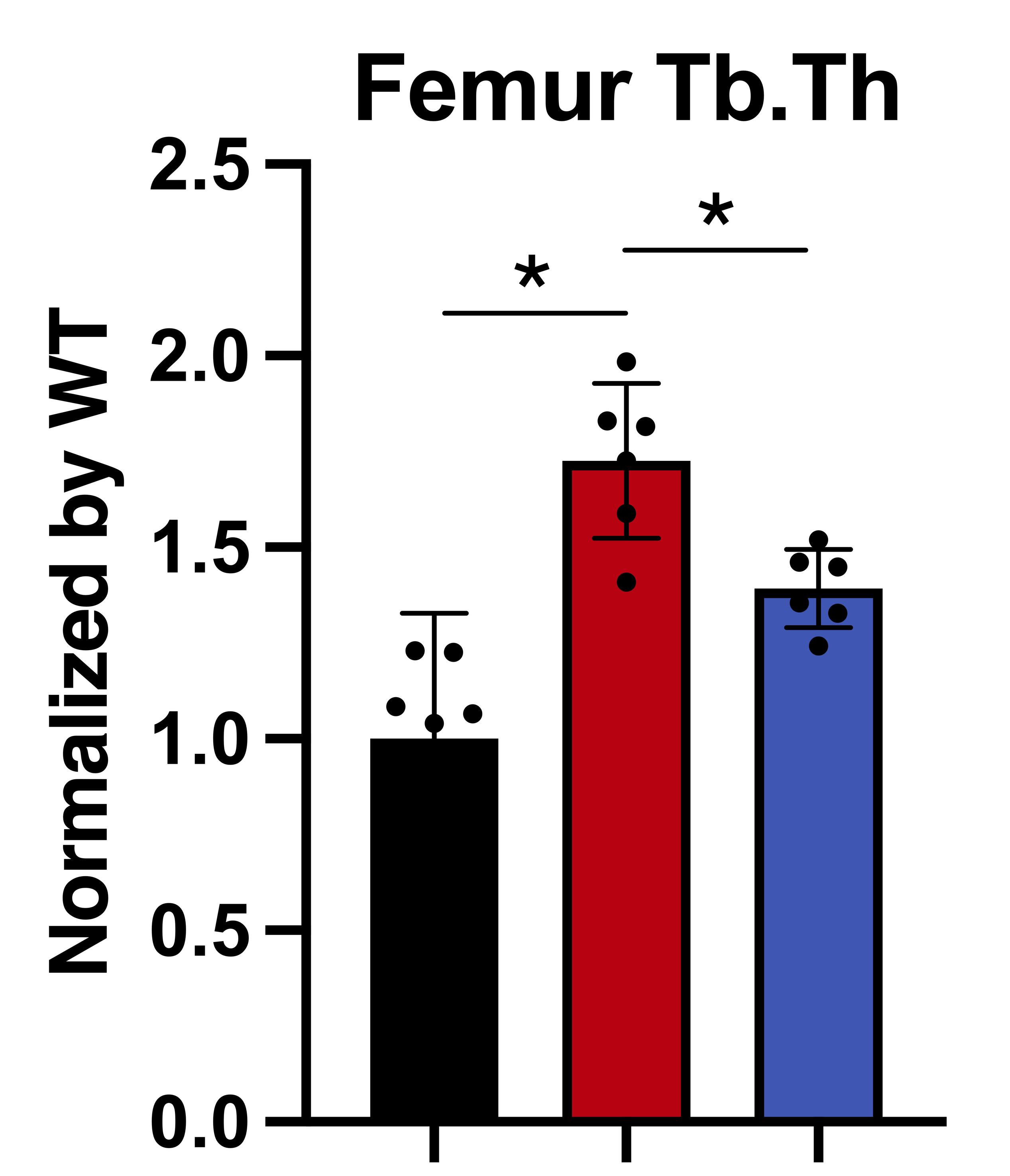

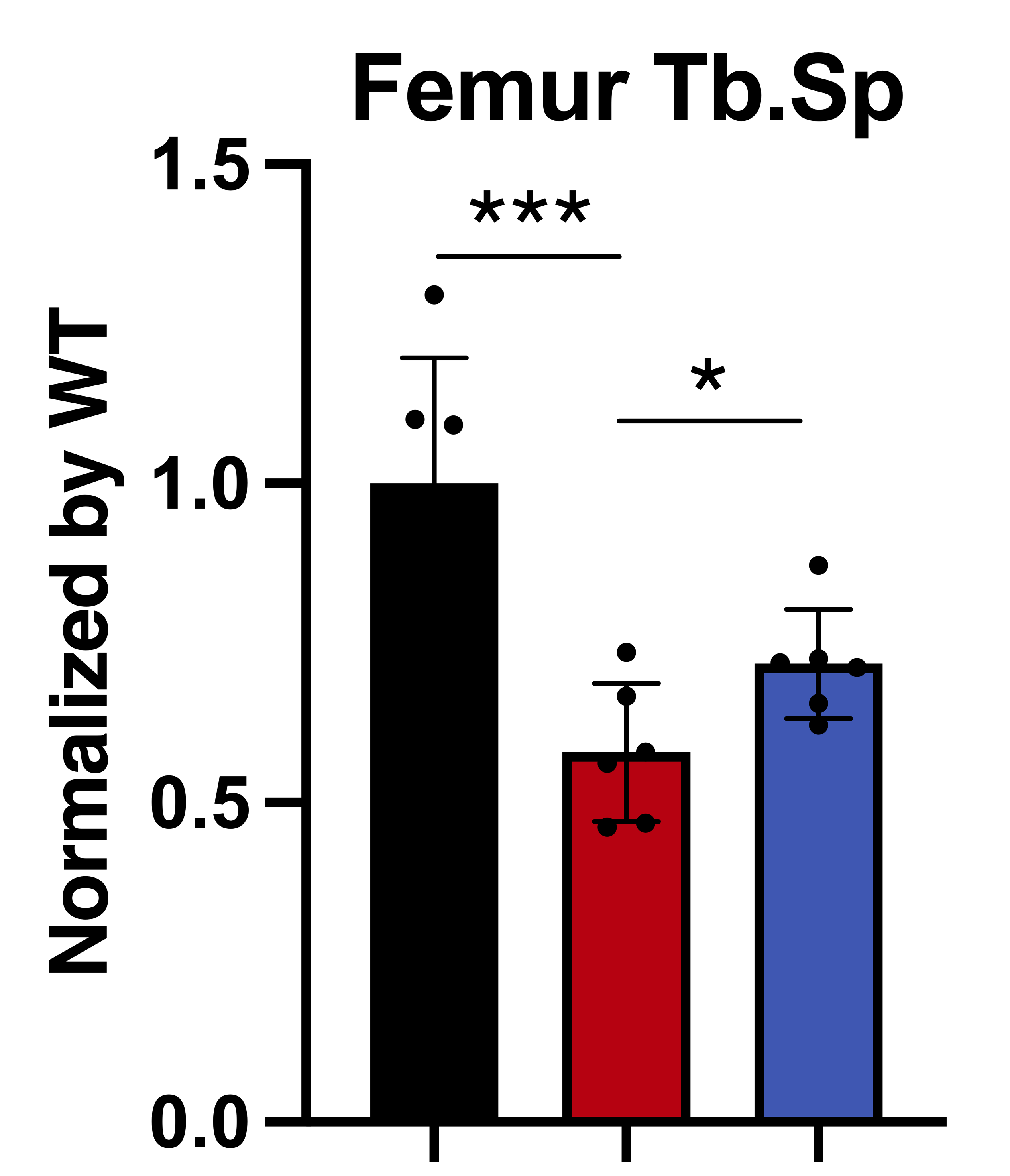

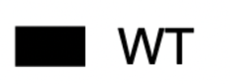

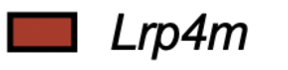

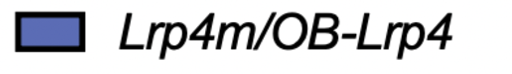

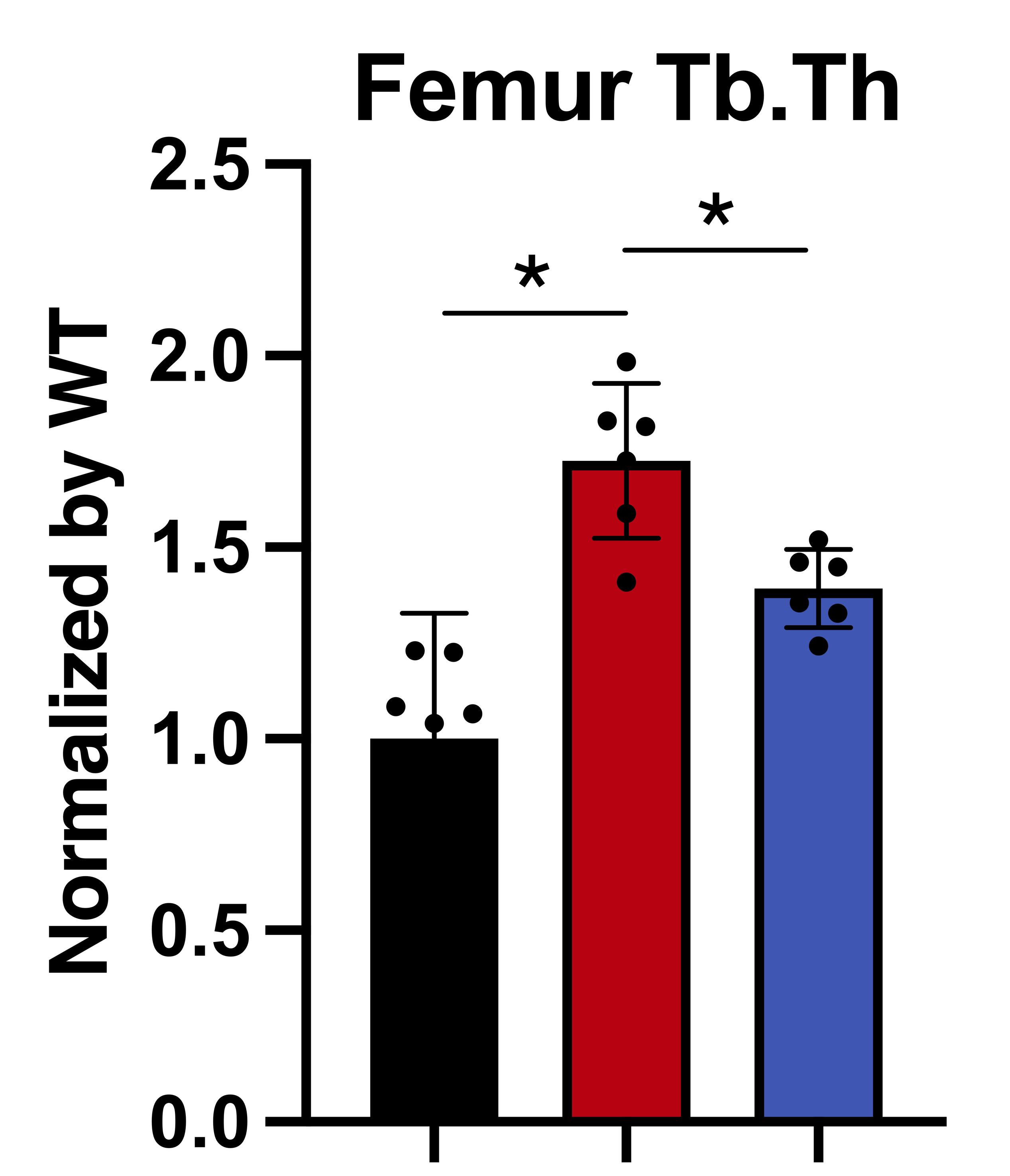

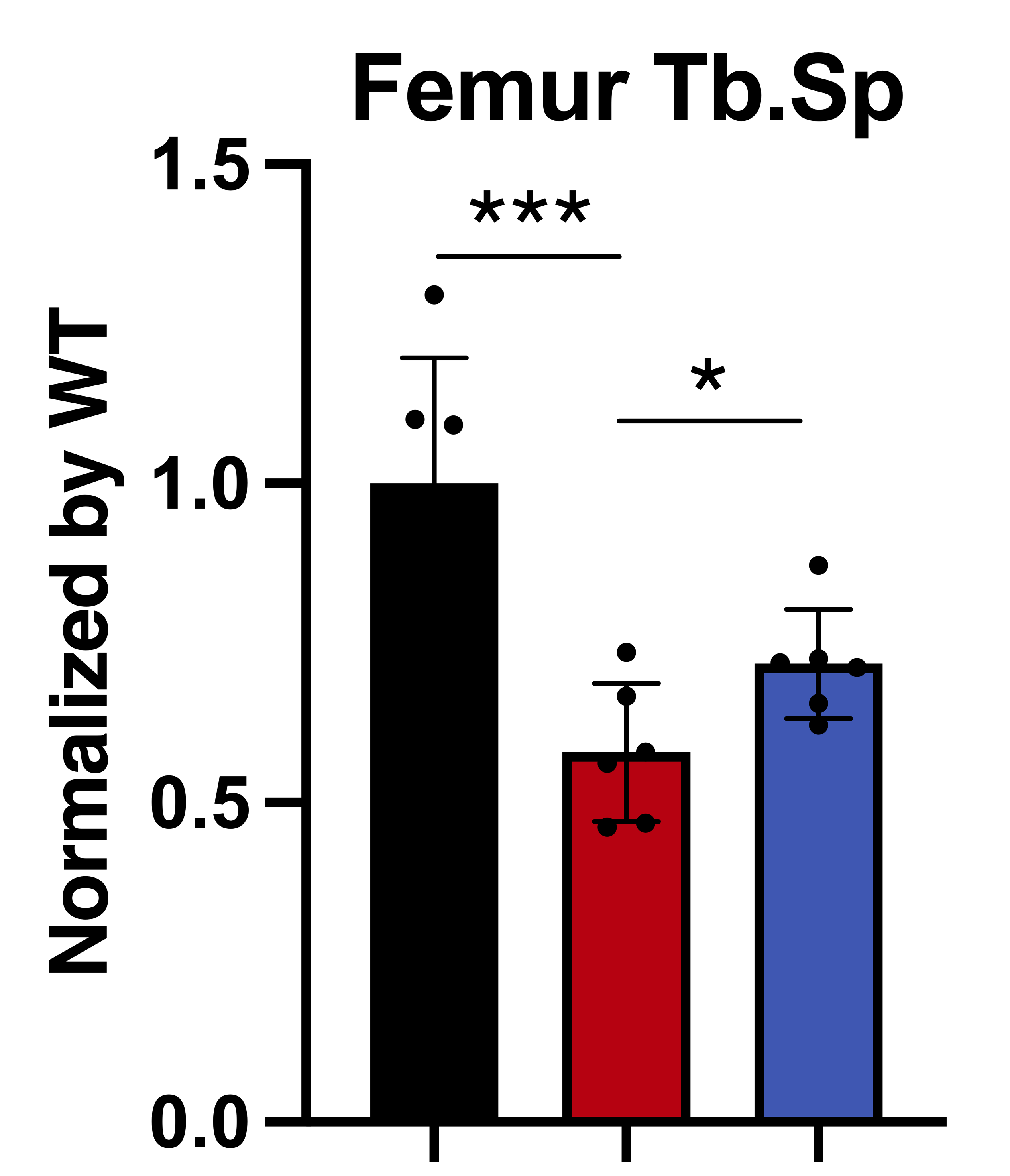

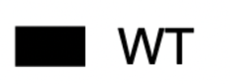

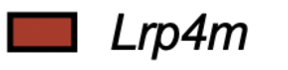

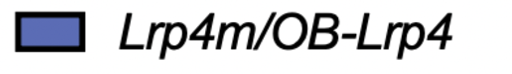

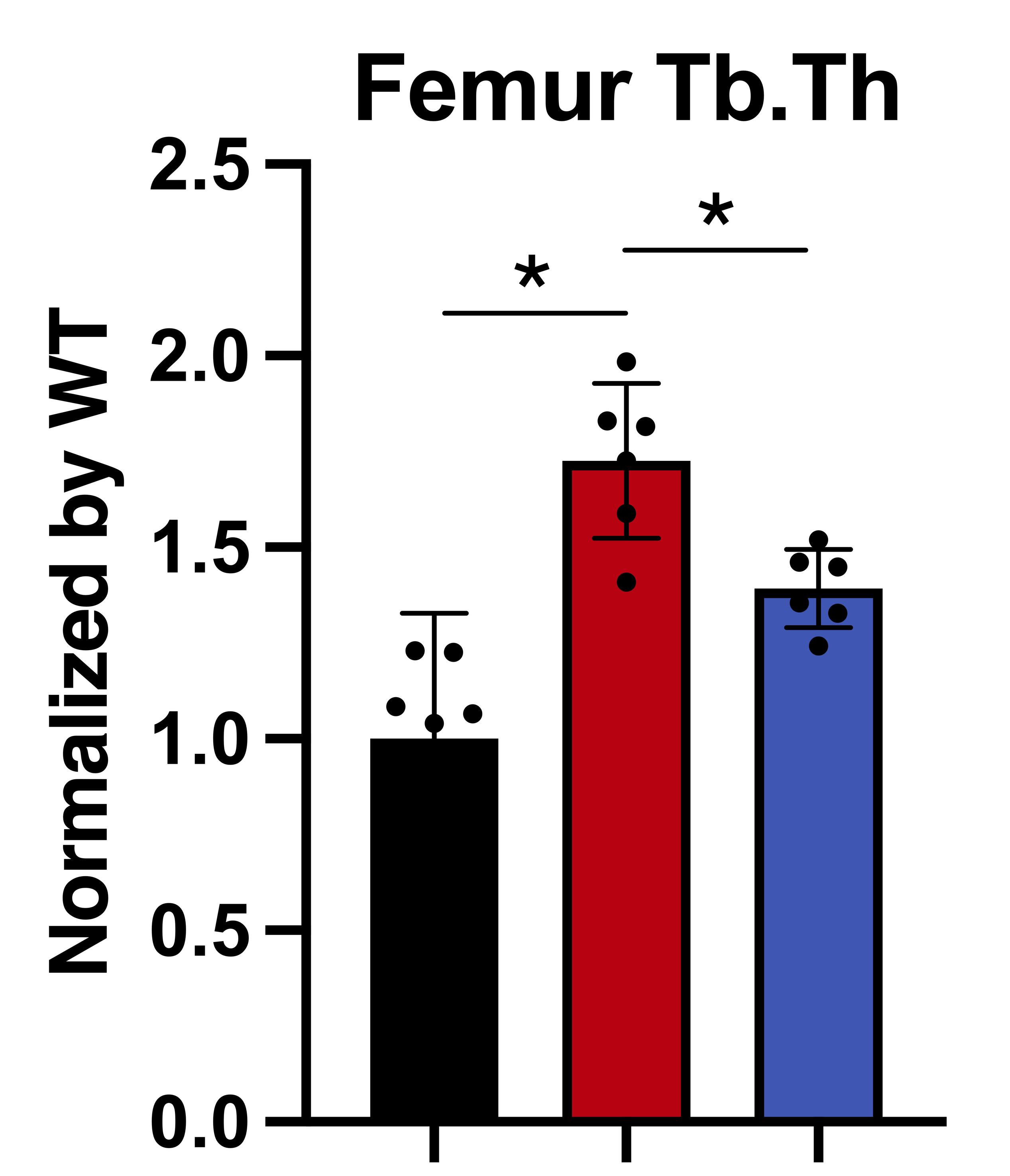

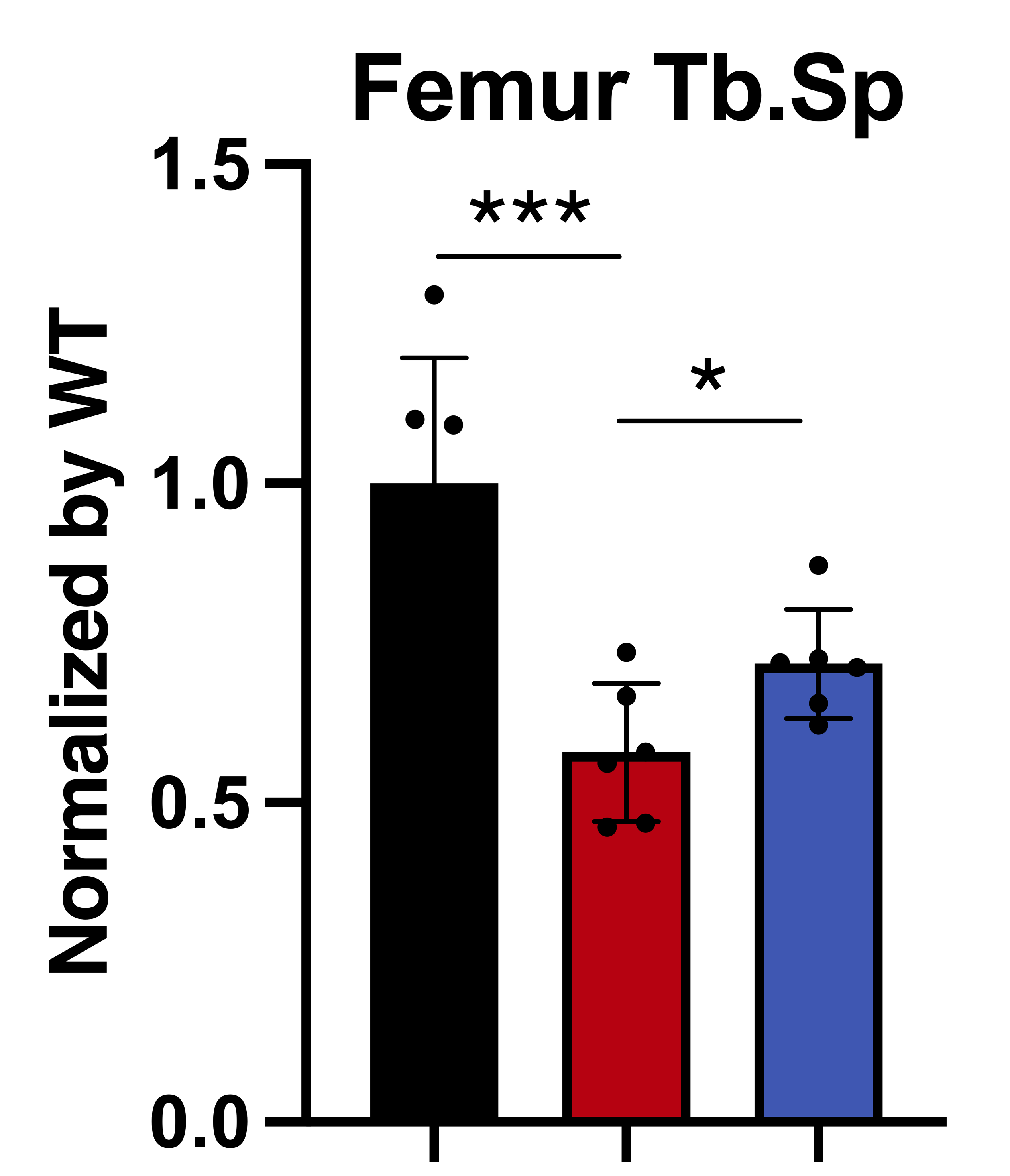

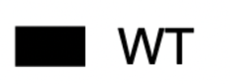

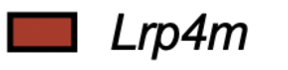

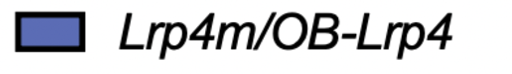


**e**


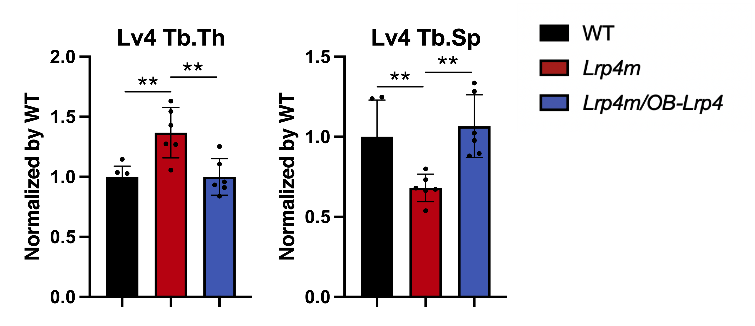


**f**

**Lv4**

**
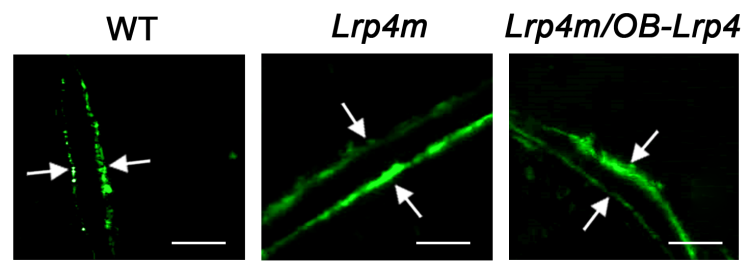
**

**g**


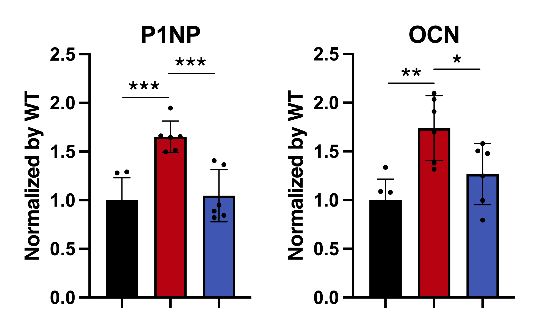

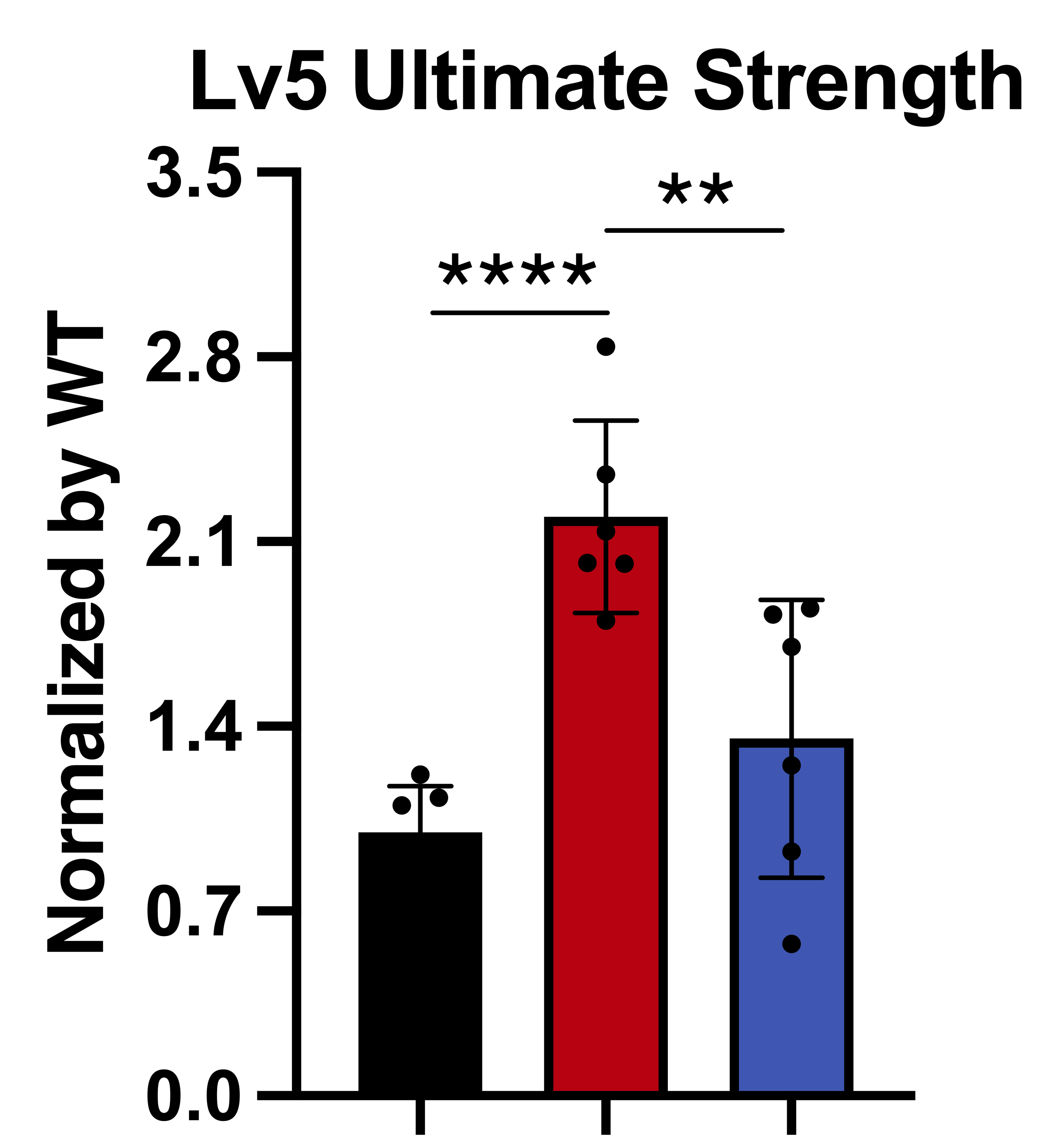


**h**


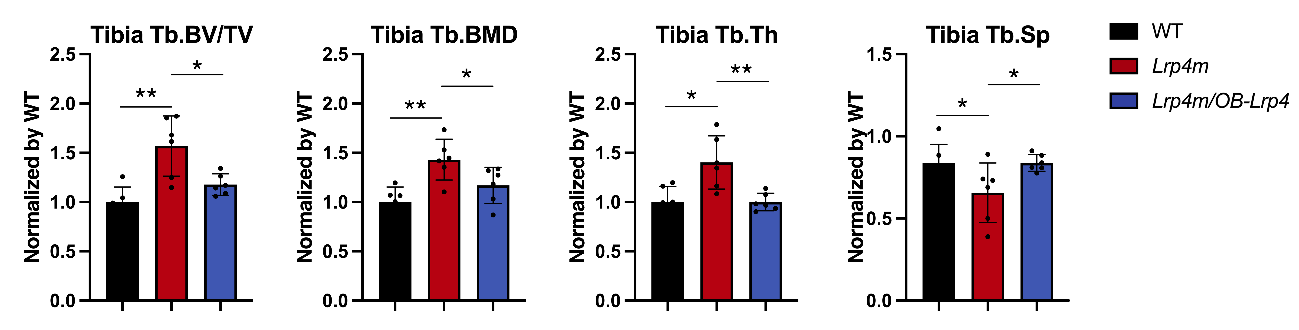


**j**

**i**


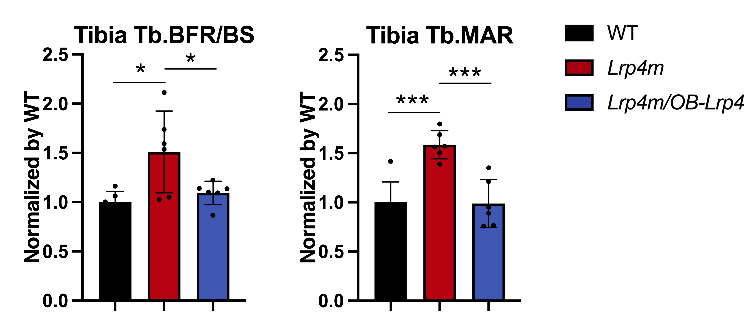


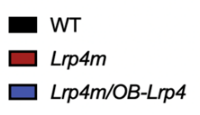


**Fig. S7. The bone phenotypes of *Lrp4m* mice, *Lrp4m/OB-Lrp4* mice and WT littermates. (a)** Bar charts of the structural parameters of Tb.Th and Tb.Sp from *ex vivo micro-CT* examination at the distal femur of *Lrp4m* mice, *Lrp4m/OB-Lrp4* mice and WT littermates. **(b)** Representative fluorescent micrographs of the trabecular bone sections showing bone formation at the distal femur, visualized by double calcein green labels. Arrows indicated the space between calcein green labeling. Scale bars, 20 μm. **(c)** Representative fluorescent micrographs of the cortical bone sections showing bone formation at the femoral mid-shaft visualized by double calcein green labels. Arrows indicated the space between calcein green labeling. Scale bars, 20 μm. **(d)** Bar charts of femur stiffness and femur fracture energy. **(e)** Bar charts of the structural parameters of Tb.Th and Tb.Sp from *ex vivo micro-CT* examination at the fourth lumbar vertebrae (Lv4). **(f)** Representative fluorescent micrographs of the trabecular bone sections showing bone formation at the Lv4, visualized by double calcein green labels. Arrows indicated the space between calcein green labeling. Scale bars, 20 μm. **(g)** Bar charts of the ultimate strength of the fifth lumbar vertebrae (Lv5). **(h)** Bar charts of the structural parameters of Tb.BV/TV, Tb.BMD, Tb.Th and Tb.Sp from *ex vivo* micro-CT examination at the proximal tibia. **(i)** Bar charts of the dynamic bone histomorphometric parameters of Tb.BFR/BS and Tb.MAR at the proximal tibia. **(j)** The serum levels of procollagen type 1 N-terminal pro-peptide (P1NP) and osteocalcin (OCN). Data were expressed as mean ± standard deviation. n = 6 per group. The unpaired t-test was used to determine the intergroup differences. ^ns^*P* > 0.05; **P* < 0.05; ***P* < 0.01; ****P* < 0.001; *****P* < 0.0001.

**a**

**b**


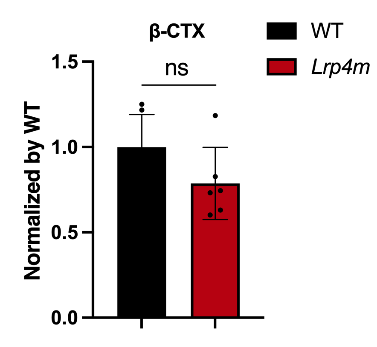

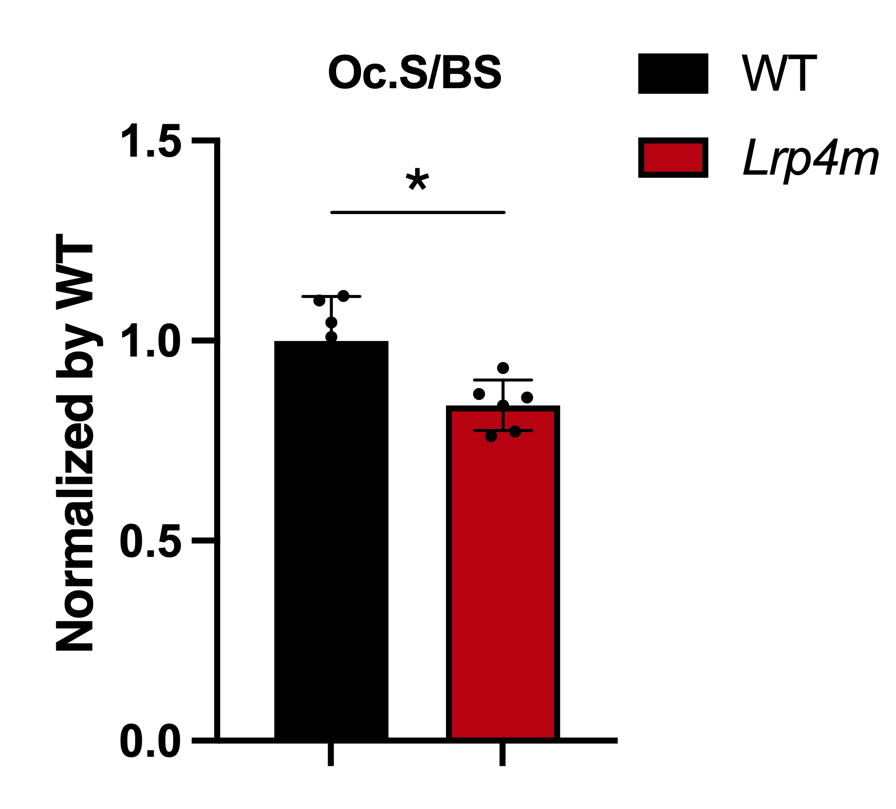

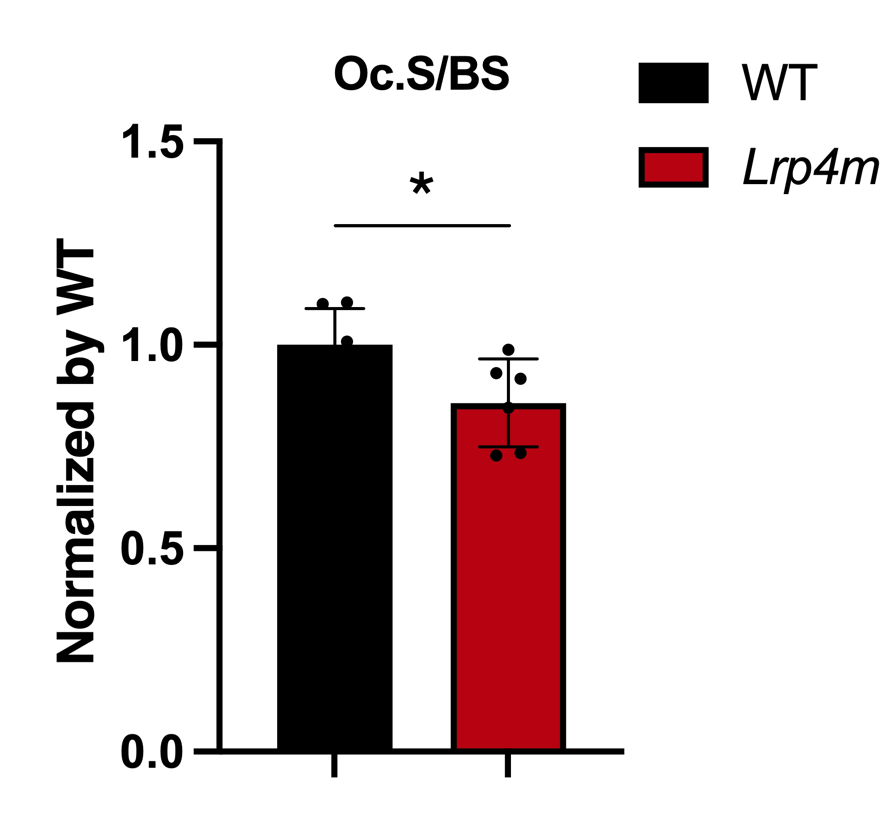


**Femoral mid-shaft**

**Distal femur**

**Fig. S8. The bone resorption parameters of *Lrp4m* mice and WT littermates. (a)** Bar charts of osteoclast surface per bone surface (Oc.S/BS) at trabecular bone of distal femur (left) and cortical bone of the femoral mid-shaft (right). **(b)** The serum level of β-CrossLaps (β-CTX). Data were expressed as mean ± standard deviation. n = 6 per group. The unpaired t-test was used to determine the intergroup differences. ^ns^*P* > 0.05; **P* < 0.05; ***P* < 0.01; ****P* < 0.001; *****P* < 0.0001.


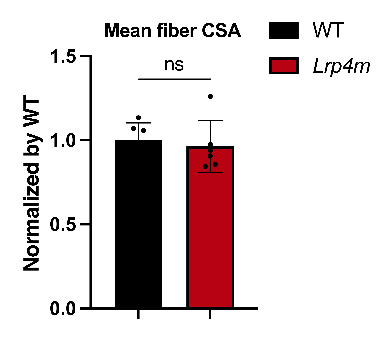

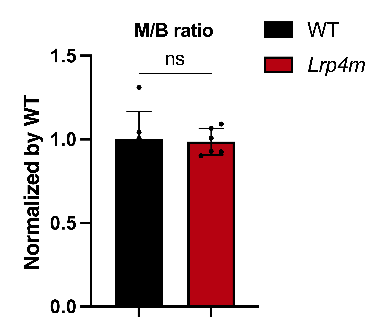

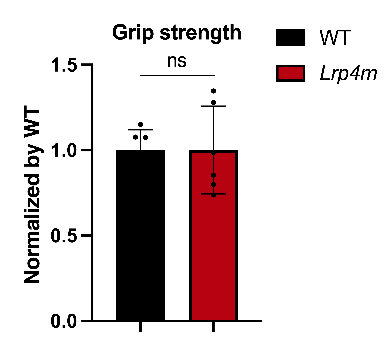


**c**

**b**

**a**


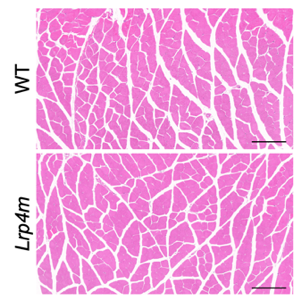


**
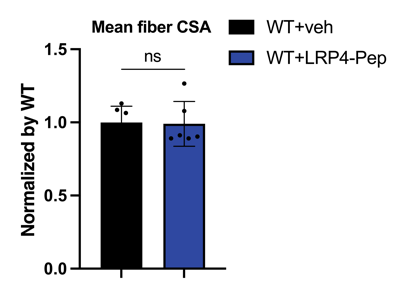

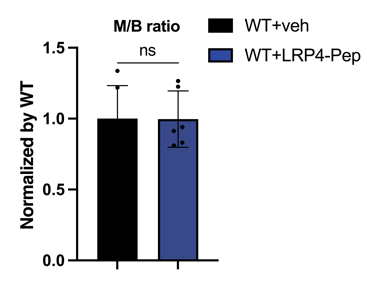

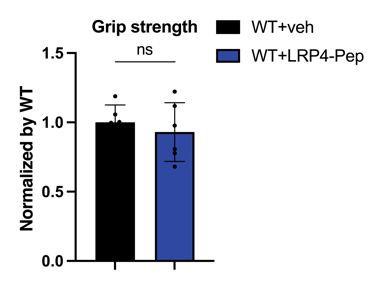
**

**f**

**d**

**e**

**
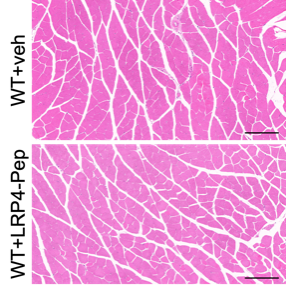
**

**Fig. S9. The effect of *Lrp4m* and LRP4-Pep on muscle in mice. (a)** Bar charts of the maximal forelimb grip strength in *Lrp4m* mice and WT littermates. **(b)** Bar charts of the gastrocnemius muscle-to-body weight ratio (M/B ratio) in *Lrp4m* mice and WT littermates. **(c)** Representative images of Hematoxylin and Eosin (H&E) staining showing the muscle fiber cross-sectional area (CSA) at the gastrocnemius muscle in *Lrp4m* mice and WT littermates (the left panel). Scale bars, 100 μm. Bar charts of the mean muscle fiber CSA (the right panel). **(d)** Bar charts of the maximal forelimb grip strength in wild-type (WT) mice with and without exogenous LRP4-Pep (10 mg/kg/day) treatment. **(e)** Bar charts of the gastrocnemius M/B ratio in WT mice with and without exogenous LRP4-Pep (10 mg/kg/day) treatment. **(f)** Representative images of H&E staining showing the muscle fiber CSA at the gastrocnemius muscle in WT mice with and without exogenous LRP4-Pep (10 mg/kg/day) treatment (the left panel). Scale bars, 100 μm. Bar charts of the mean muscle fiber CSA (the right panel).


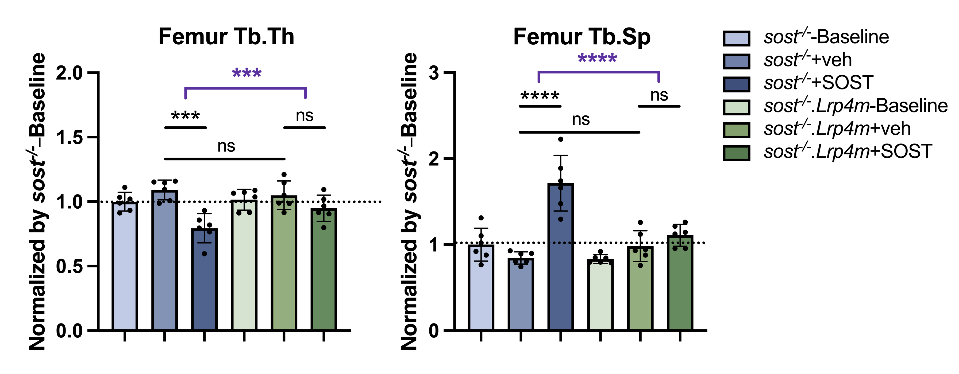


**a**

**b**

**Distal femur**


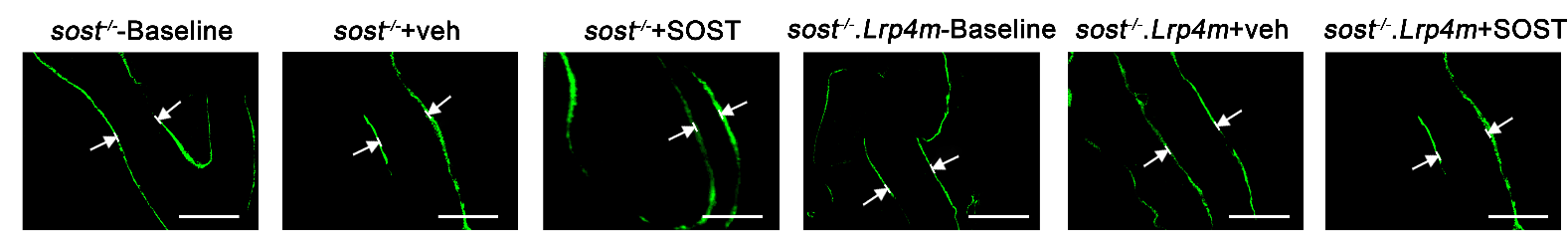


**c**

**Femoral mid-shaft**


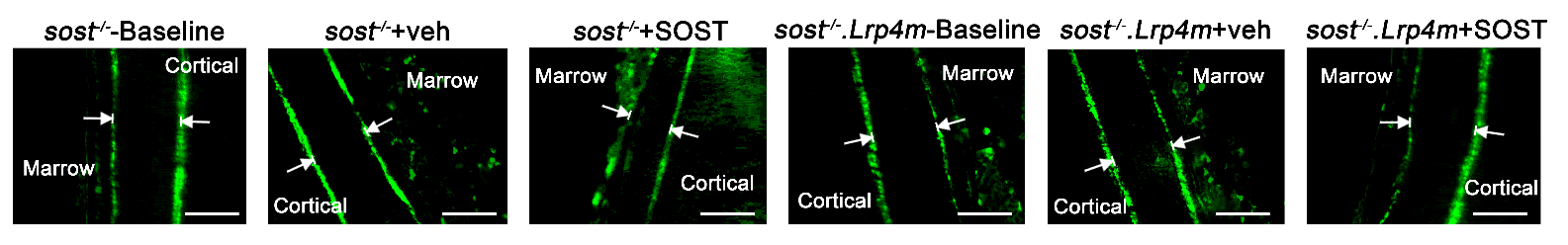


**e**

**d**


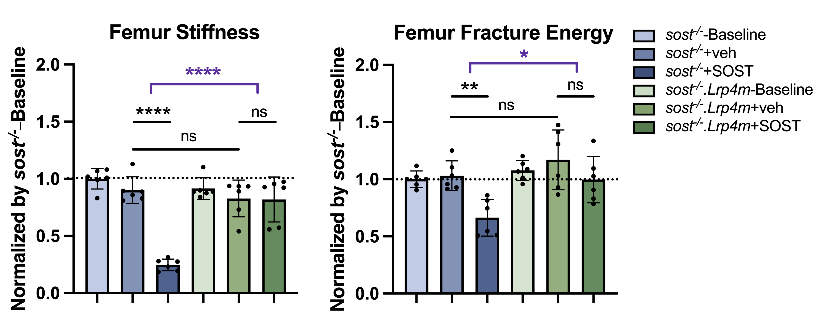


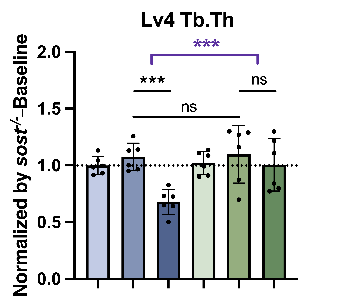

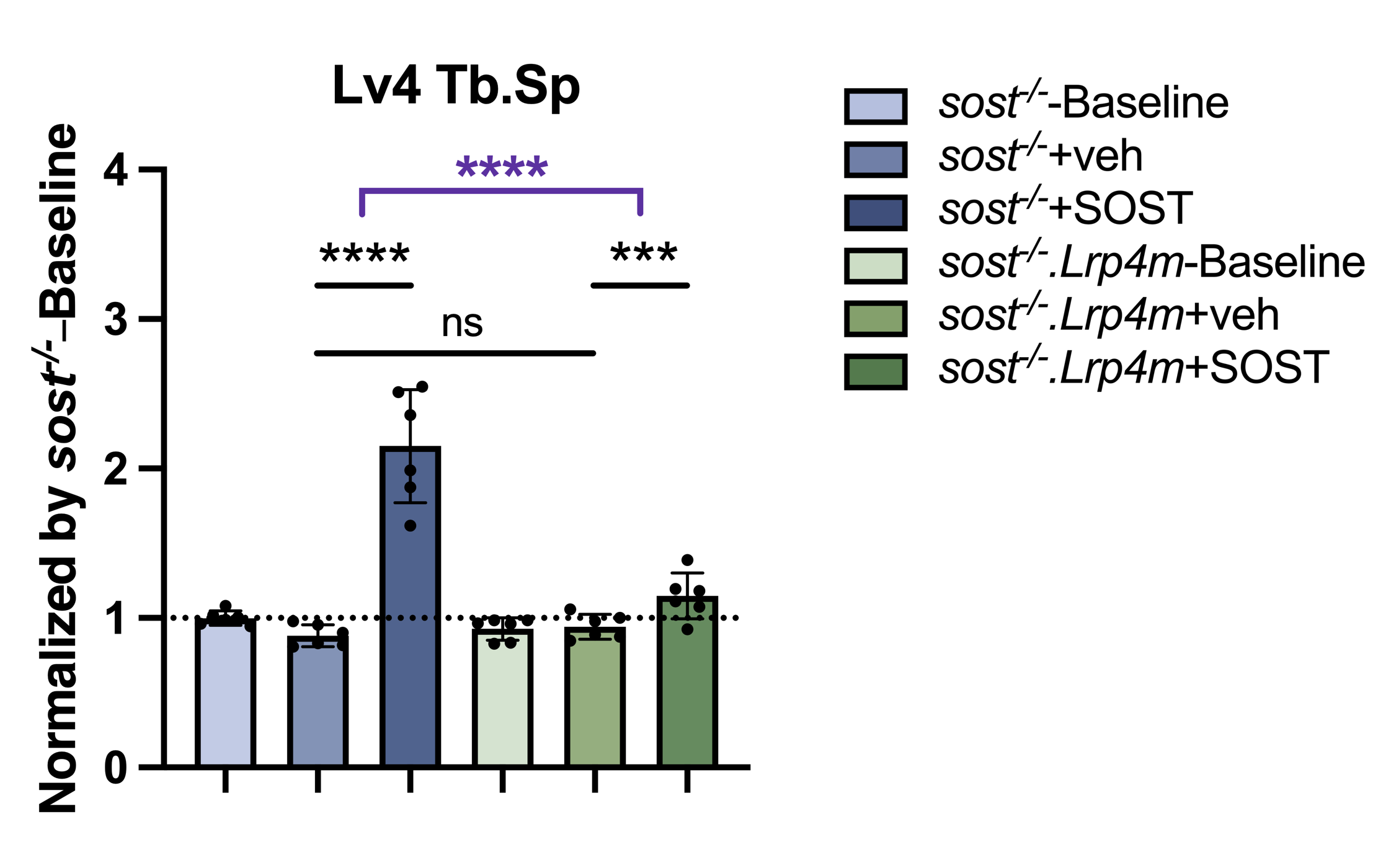


**f**

**Lv4**


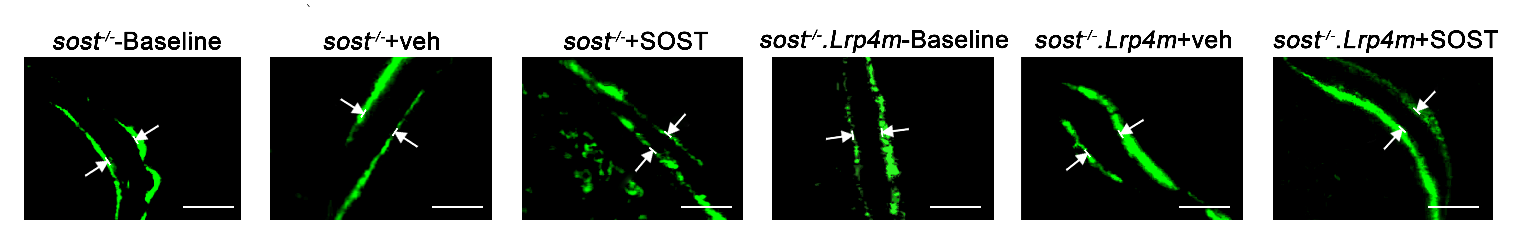


**g**


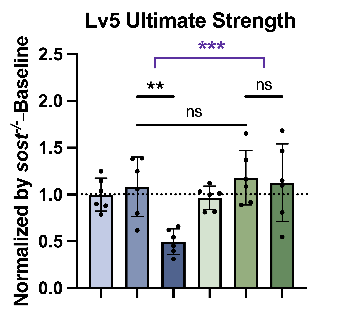


**h**


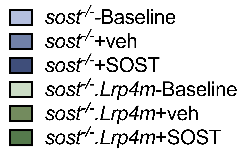


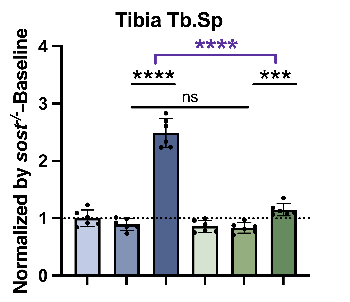

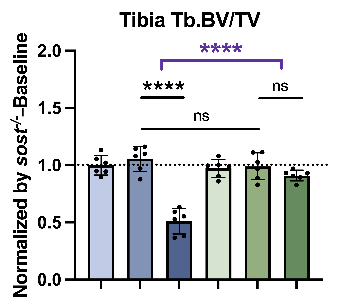

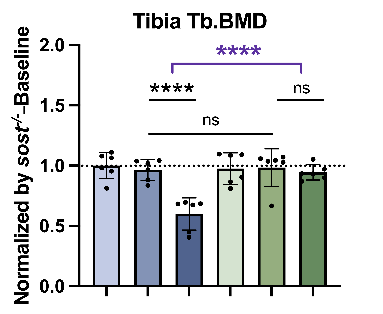

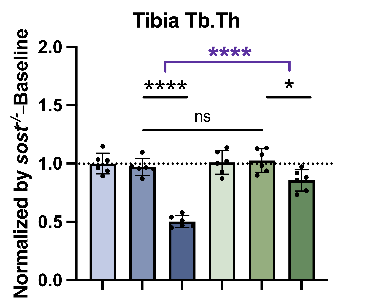


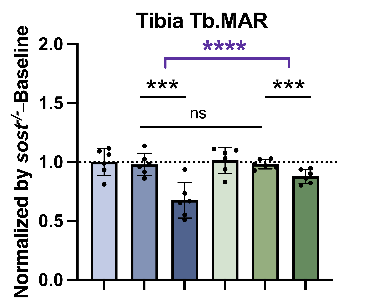

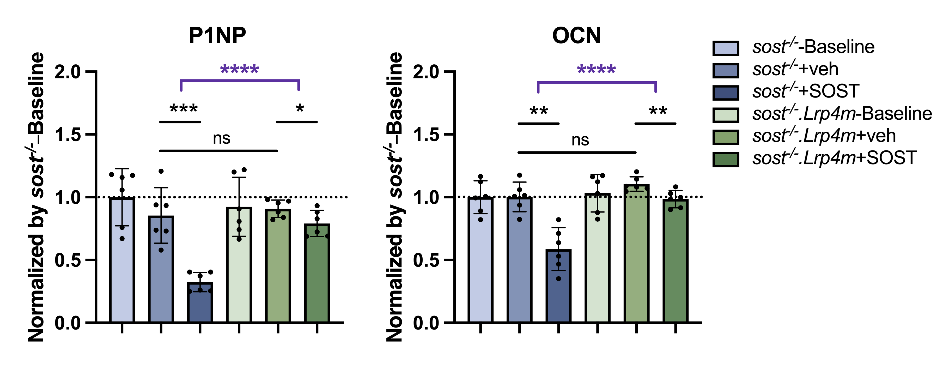

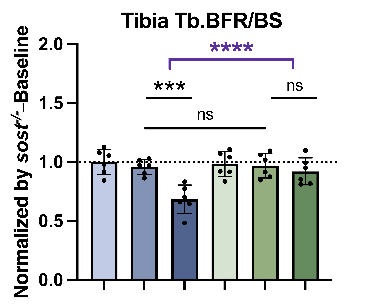


**i**

**j**

**Fig. S10. The bone formation of *sost^-/-^* mice and *sost^-/-^.Lrp4m* mice, with and without *rAAV9*-mediated re-expression of sclerostin. (a)** Bar charts of the structural parameters of Tb.Th and Tb.Sp from *ex vivo* micro-CT examination at the distal femur. **(b)** Representative fluorescent micrographs of the trabecular bone sections showing bone formation at the distal femur, visualized by double calcein green labels. Arrows indicated the space between calcein green labeling. Scale bars, 20 μm. **(c)** Representative fluorescent micrographs of the cortical bone sections showing bone formation at the femoral mid-shaft visualized by double calcein green labels. Arrows indicated the space between calcein green labeling. Scale bars, 20 μm. **(d)** Bar charts of the stiffness and fracture energy at the femoral mid-shaft. **(e)** Bar charts of the structural parameters of Tb.Th and Tb.Sp from *ex vivo micro-CT* examination at the Lv4 (the right panel). **(f)** Representative fluorescent micrographs of the trabecular bone sections showing bone formation at the Lv4, visualized by double calcein green labels. Arrows indicated the space between calcein green labeling. Scale bars, 20 μm. **(g)** Bar charts of ultimate strength at the Lv5. **(h)** Bar charts of the structural parameters of Tb.BV/TV, Tb.BMD, Tb.Th and Tb. Sp from *ex vivo* micro-CT examination at the proximal tibia. **(i)** Bar charts of the dynamic bone histomorphometric parameters of Tb.BFR/BS and Tb.MAR at the proximal tibia. **(j)** Bar charts of serum levels of procollagen type 1 N-terminal pro-peptide (P1NP) and osteocalcin (OCN). Data were expressed as mean ± standard deviation. n = 6 per group. The unpaired t-test was used to determine the intergroup differences; ^ns^*P* > 0.05; **P* < 0.05; ***P* < 0.01; ****P* < 0.001; *****P* < 0.0001 in black color. The unpaired t-test was also used to determine the difference between Means (*sost^-/-^.Lrp4m* + SOST) - (*sost^-/-^.Lrp4m* + veh) ± SD ***vs.*** Means (*sost^-/-^* + SOST) - (*sost^-/^*^-^ + veh) ± SD; ^ns^*P* > 0.05; **P* < 0.05; ***P* < 0.01; ****P* < 0.001; *****P* < 0.0001 in purple color.

**
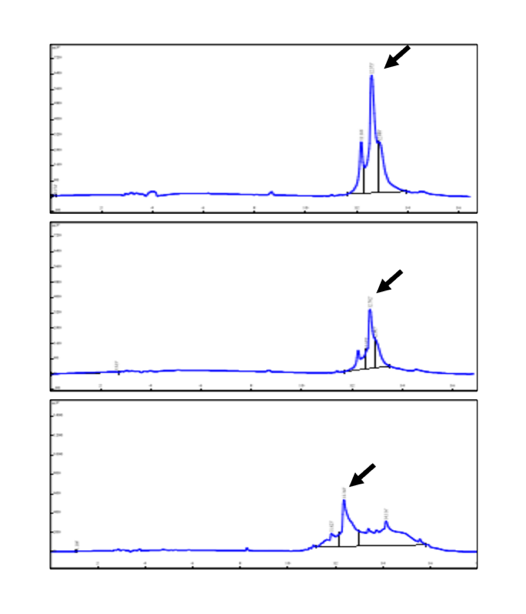
**

**c**


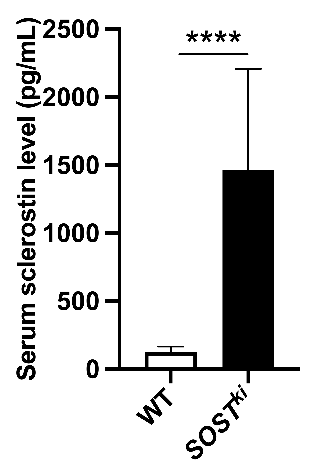

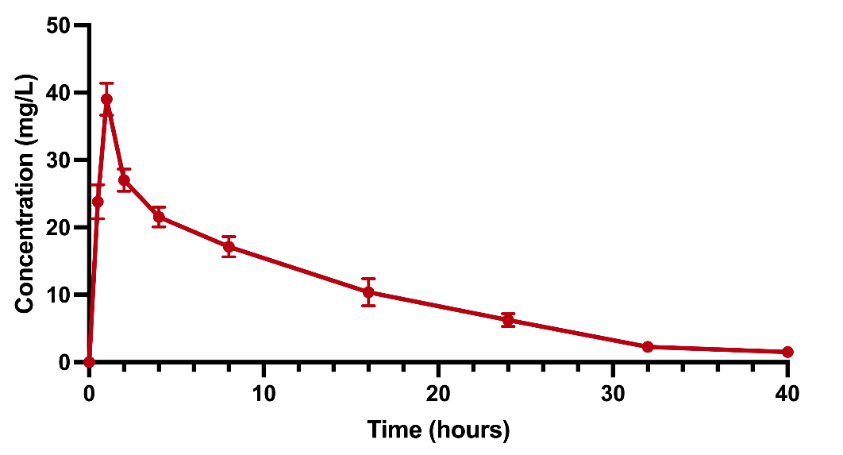


**b**

**a**

**e**


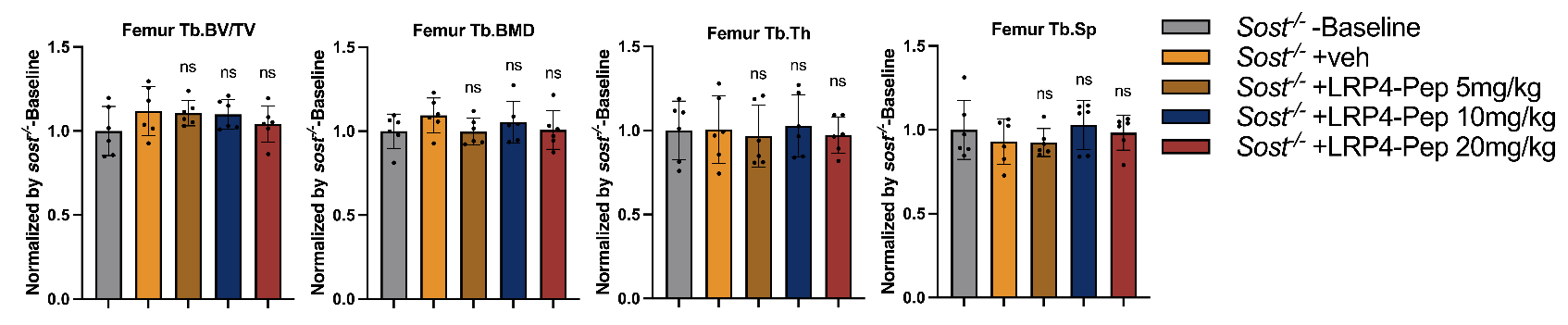


**d**


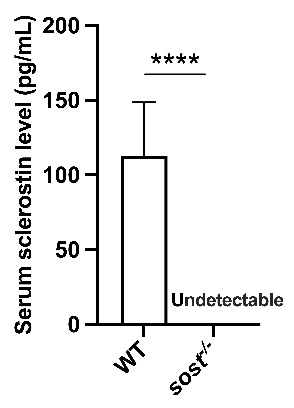


**Fig. S11. Determination of the administration dosage, interval and duration of LRP4-Pep *in vivo.* (a)** Serum stability of the modified LRP4-Pep: Representative HPLC images of the modified LRP4-Pep at baseline (upper), 2 hours (middle) and 4 hours (lower) in 90% mouse serum, respectively. **(b)** Pharmacokinetics of LRP4-Pep in mice, determined by HPLC. Analyses were repeated 3 times for each serum sample. **(c)** Serum sclerostin levels in *SOST^ki^* mice and WT littermates. **(d)** Serum sclerostin levels in *sost^-/-^* mice and WT littermates. **(c-d)** Unpaired t-test was used to determine the intergroup difference. **(e)** Bar charts of the structural parameters of Tb.BV/TV, Tb.BMD, Tb.Th and Tb.Sp from *ex vivo* micro-CT examination at the distal femur of *sost^-/-^* mice, with/without treatment of LRP4-Pep at a dose of 5 mg/kg, 10 mg/kg and 20 mg/kg, respectively. Data were expressed as mean ± standard deviation followed by one-way ANOVA with Tukey’s post-hoc test *vs.* *sost^-/-^* + veh group. ^ns^*P* > 0.05; **P* < 0.05; ***P* < 0.01; ****P* < 0.001; *****P* < 0.0001.


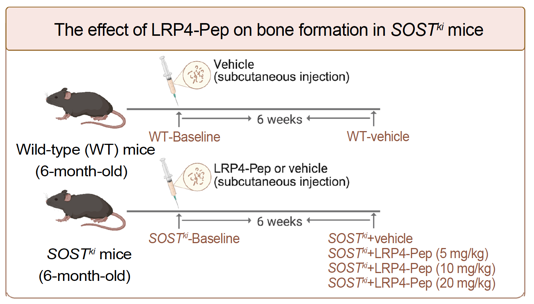


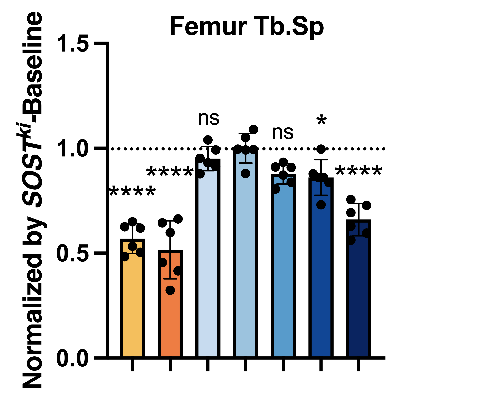

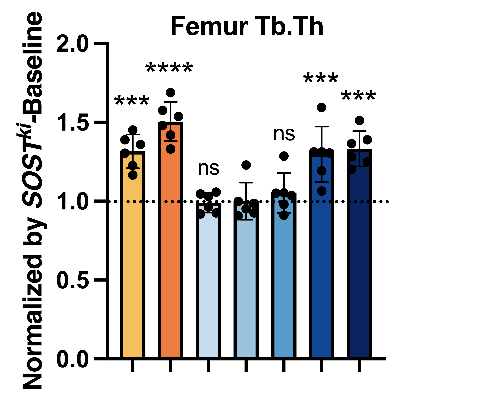

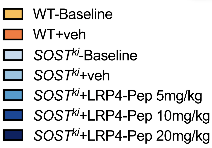


**a**

**b**

**c**

**Distal femur**


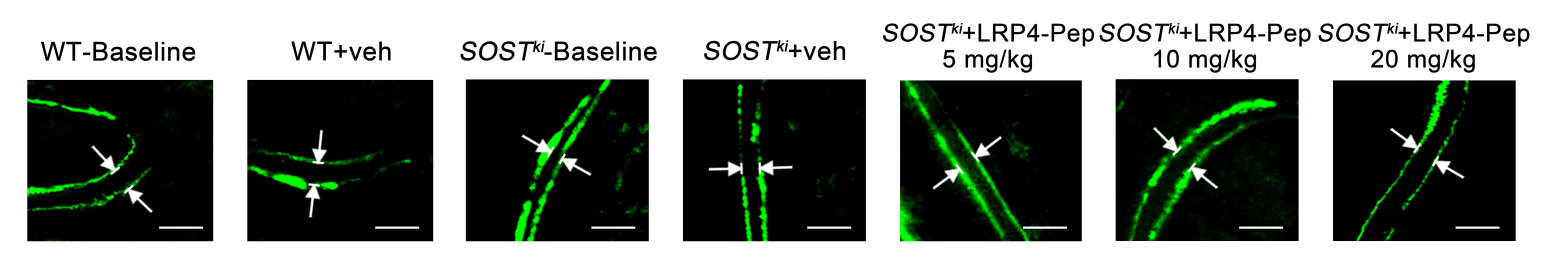


**d**

**Femoral mid-shaft**


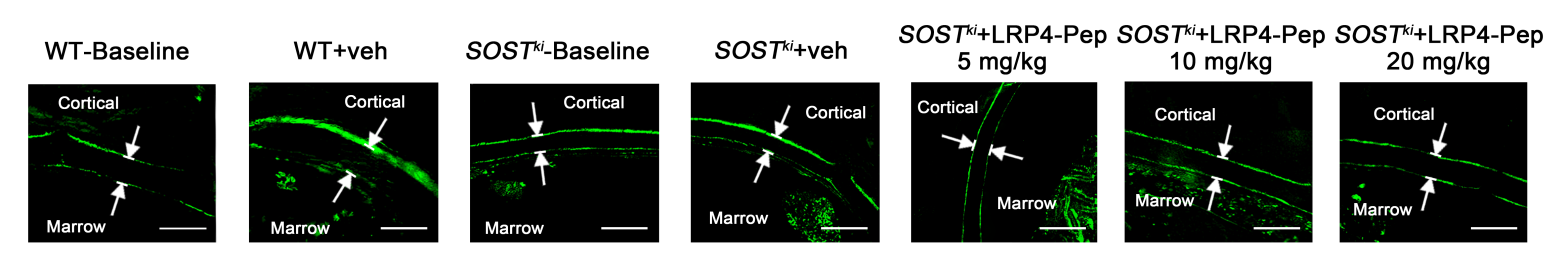


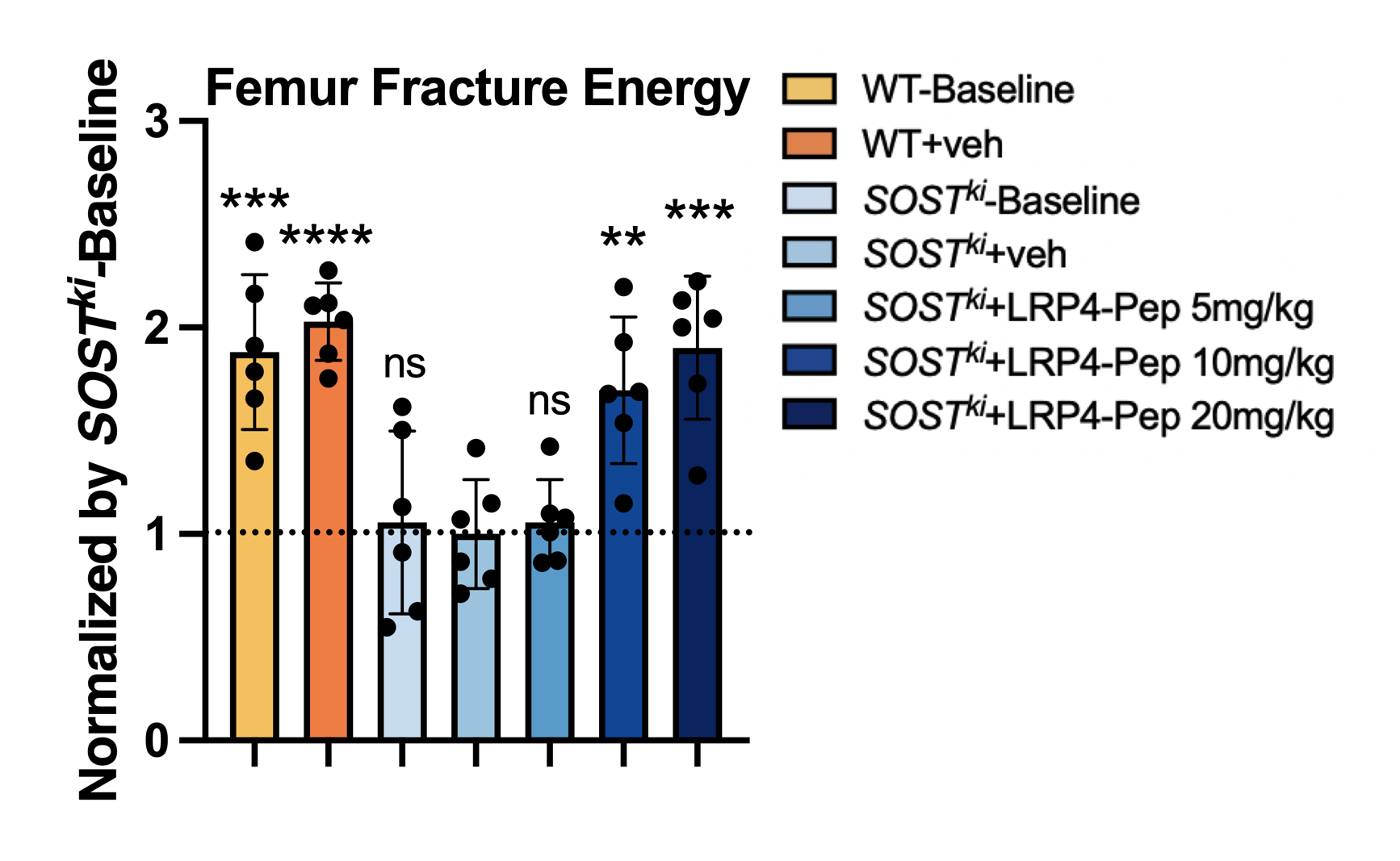

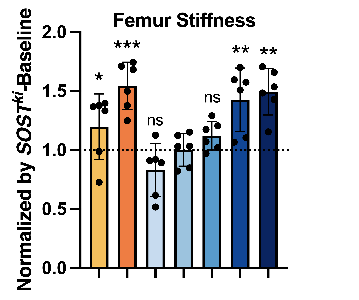


**e**


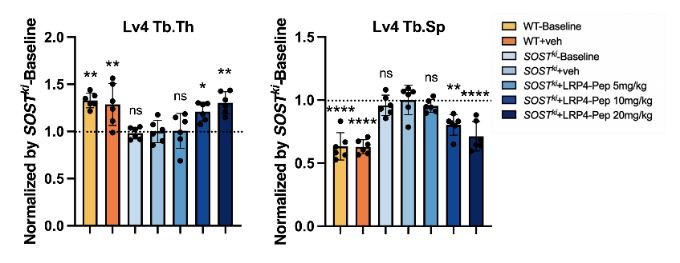


**f**


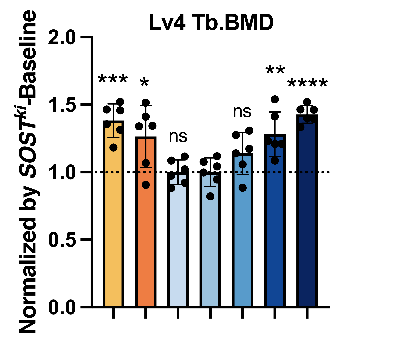


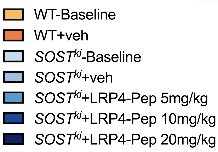


**g**

**Lv4**


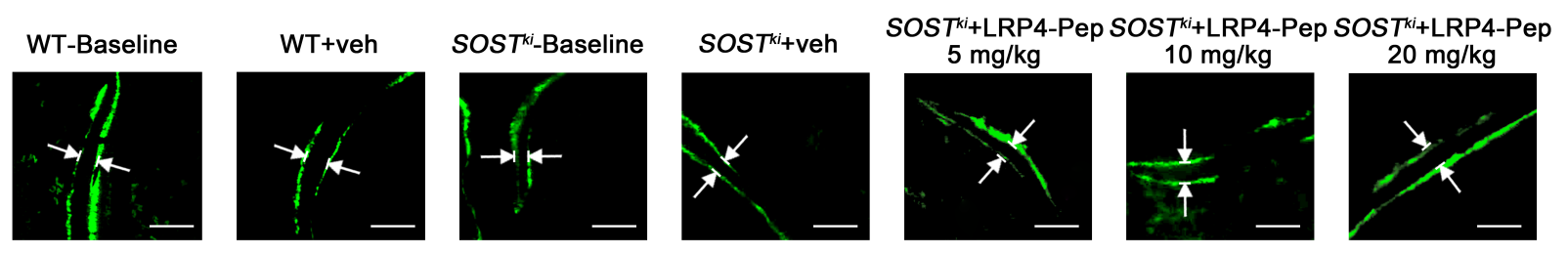


**h**

**i**


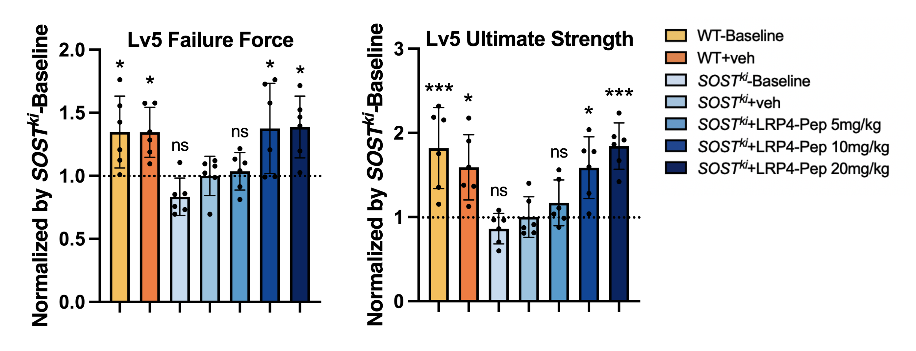


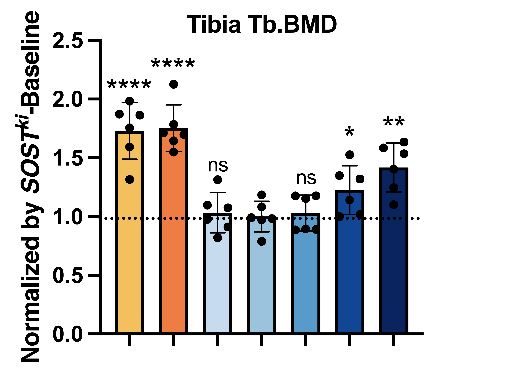

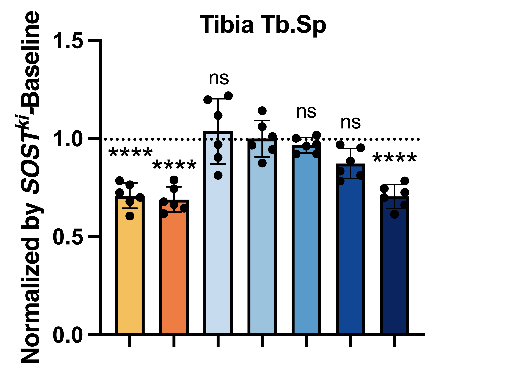

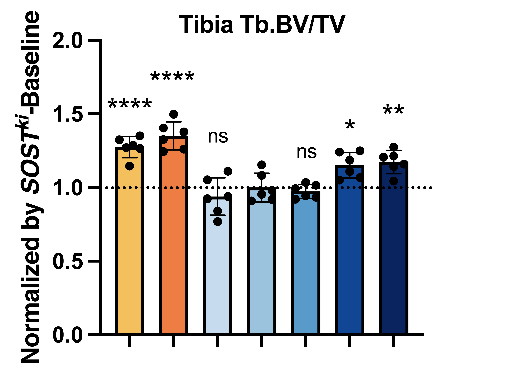

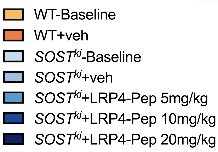


**j**


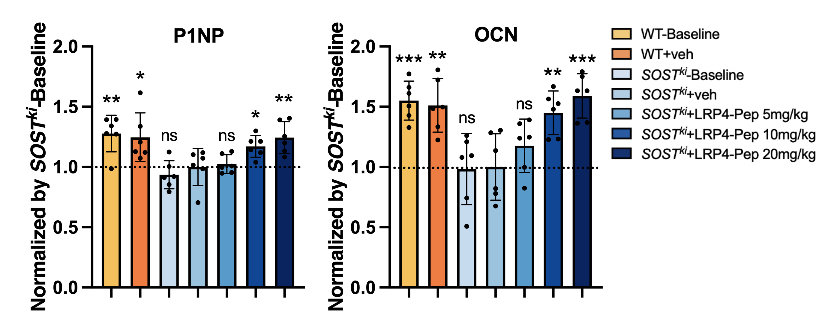


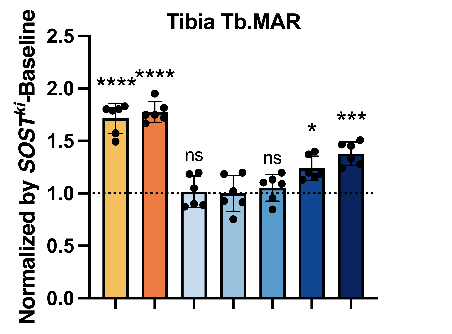

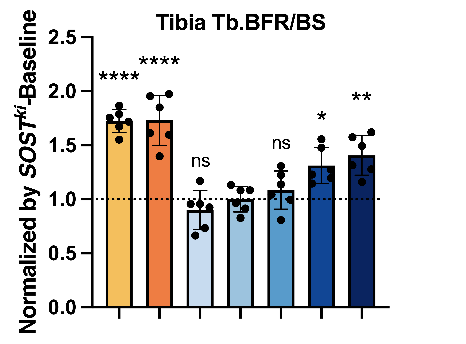


**k**

**Fig. S12. The influence of the exogenous LRP4-Pep in the antagonistic effect of sclerostin on bone formation in *SOST^ki^* mice. (a)** The diagram of experimental design. **(b)** Bar charts of the structural parameters of Tb.Th and Tb.Sp from *ex vivo micro-CT* examination at the distal femur. **(c)** Representative fluorescent micrographs of the trabecular bone sections showing bone formation at the distal femur, visualized by double calcein green labels. Arrows indicated the space between calcein green labeling. Scale bars, 20 μm. **(d)** Representative fluorescent micrographs of the cortical bone sections showing bone formation at the femoral mid-shaft, visualized by double calcein green labels. Arrows indicated the space between calcein green labeling. Scale bars, 20 μm. **(e)** Bar charts of femur stiffness and femur fracture energy. **(f)** Bar charts of the structural parameters of Tb.BMD, Tb.Th and Tb.Sp from *ex vivo micro-CT* examination at the Lv4. **(g)** Representative fluorescent micrographs of the trabecular bone sections showing bone formation at the Lv4, visualized by double calcein green labels. Arrows indicated the space between calcein green labeling. Scale bars, 20 μm. **(h)** Bar charts of ultimate strength at the Lv5. **(i)** Bar charts of the structural parameters of Tb.BV/TV, Tb.BMD and Tb.Sp from *ex vivo micro-CT* examination at the proximal tibia. **(j)** Bar charts of dynamic bone histomorphometric parameters of Tb.BFR/BS and Tb.MAR at the proximal tibia. **(k)** Bar charts of serum levels of procollagen type 1 N-terminal pro-peptide (P1NP) and osteocalcin (OCN). Data were expressed as mean ± standard deviation. n = 6 per group. One-way ANOVA with Tukey’s post-hoc test vs. *SOST^ki^* + veh group was used to determine the intergroup differences. ^ns^*P* > 0.05; **P* < 0.05; ***P* < 0.01; ****P* < 0.001; *****P* < 0.0001.

**a**

**b**

**c**

**d**

**
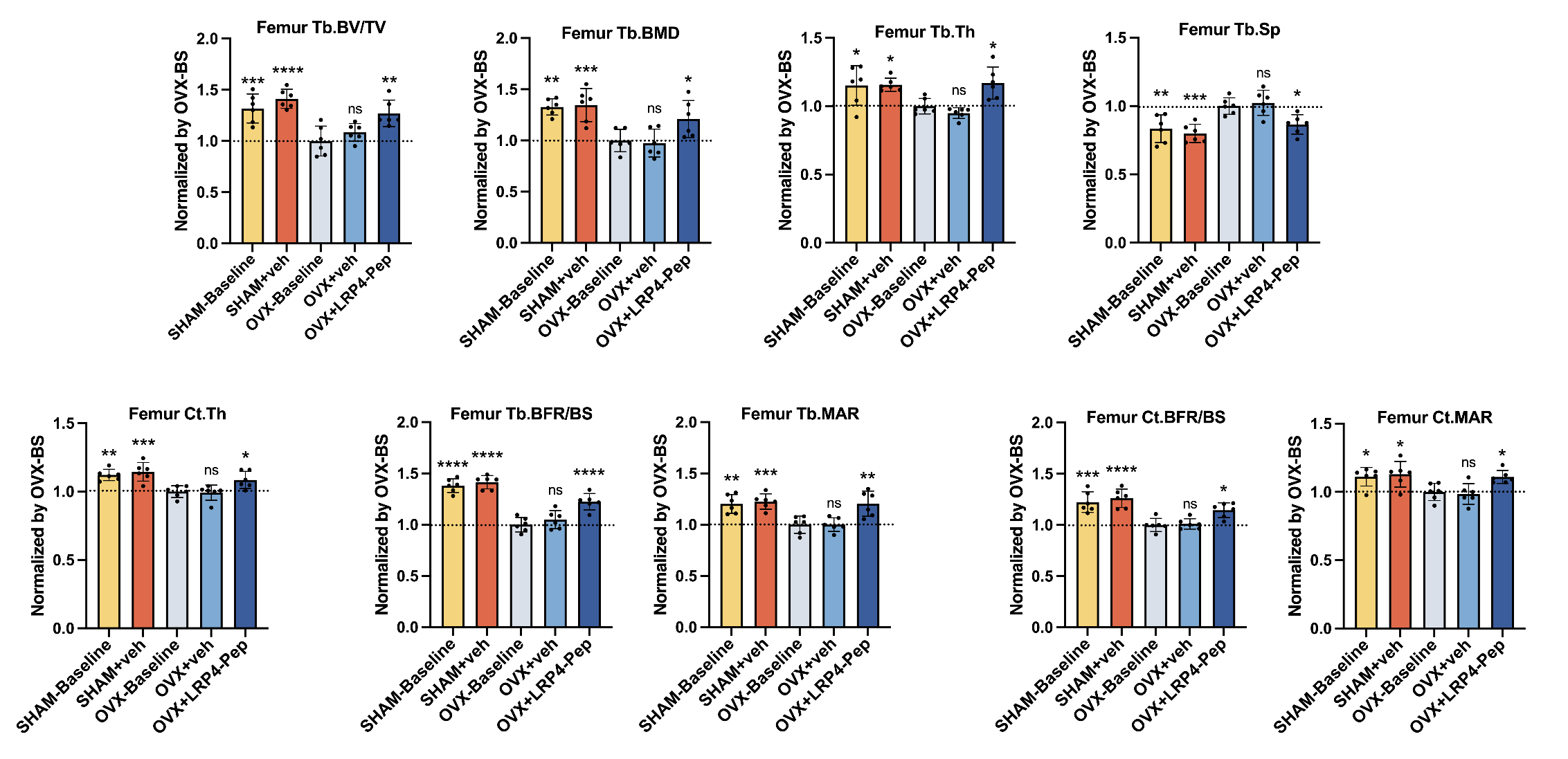
**

**Fig. S13. The effect of the exogenous LRP4-Pep on bone formation in OVX mice. (a)** Bar charts of the structural parameters of Tb.BV/TV, Tb.BMD, Tb.Th and Tb.Sp from *ex vivo micro-CT* examination at distal femur. **(b)** Bar charts of the structural parameters of Ct.Th from *ex vivo micro-CT* examination at the femoral mid-shaft. **(c)** Bar charts of the dynamic bone histomorphometric parameters of Tb.BFR/BS and Tb.MAR at distal femur. **(d)** Bar charts of the dynamic bone histomorphometric parameters of Ct.BFR/BS and Ct.MAR at the femoral mid-shaft. Data were expressed as mean ± standard deviation followed by one-way ANOVA with Tukey’s post-hoc test *vs* OVX-Baseline group. n = 6 per group. ^ns^*P* > 0.05; **P* < 0.05; ***P* < 0.01; ****P* < 0.001; *****P* < 0.0001.

**a**

**b**


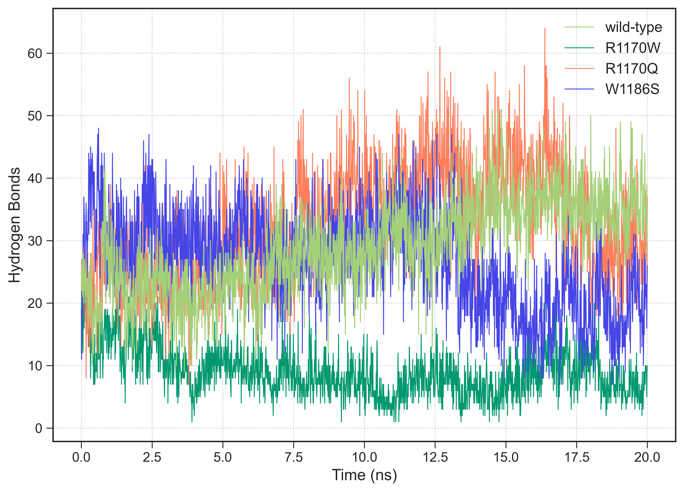

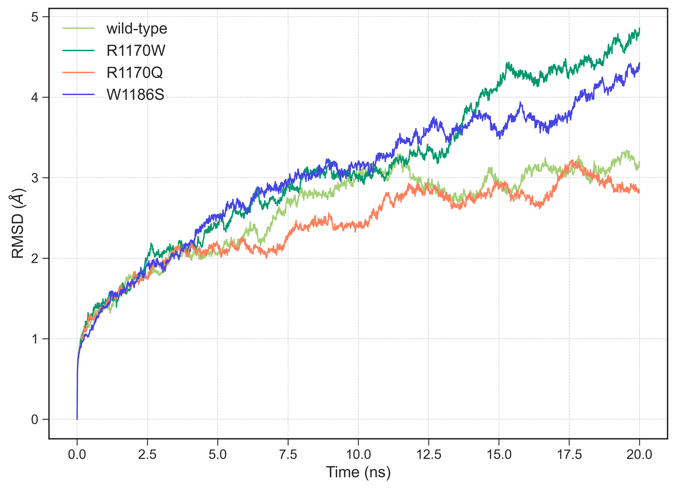


**Fig. S14. Molecular dynamics simulation of interaction between sclerostin and wild-type/mutant LRP4s, with or without LRP6. (a)** Root-mean-square deviation (RMSD) of the complex of sclerostin, wild-type/mutant LRP4s and LRP6 over time. **(b)** Number of hydrogen bonds binding between sclerostin loop3 and LA5 of wild-type/mutant LRP4. The number of hydrogen bonds binding between sclerostin loop3 and LRP4 LA5 was dramatically reduced upon both LRP4 R1170W and LRP4 W1186S, respectively, while the RMSD among sclerostin-LRP4-LRP6 were dramatically higher.

**Fig. S15**. **The binding pocket within the LA5 domain of LRP4, identified by the DoGSiteScorer server, is highlighted in red.** The binding pocket within the LA5 domain of LRP4 exhibited a high drug score of 0.84, along with moderate depth.

**Table S1. Full-length and truncated LRP4** ([Q8VI56](https://www.uniprot.org/uniprotkb/Q8VI56/entry)) **containing different domains**

| **Peptides ID** | **Length** | **Domains of LRP4** | **Molecular weight (kDa)** |
| --- | --- | --- | --- |
| LRP4-FL | 1-1905 | FL | 212 |
| LRP4-T1 | 1-1725 | Topological domain (Extracellular: LA 1-8, EGF like 1-3, LB 1-20, Polar residues) | 192 |
| LRP4-T2 | 1-1306 | LA 1-8, EGF like 1-3, LB 1-15 | 146 |
| LRP4-T3 | 1-998 | LA 1-8, EGF like 1-3, LB 1-10 | 112 |
| LRP4-T4 | 1-737 | LA 1-8, EGF like 1-3, LB 1-5 | 82 |
| LRP4-T5 | 1-434 | LA 1-8, EGF like 1-2 | 48 |
| LRP4-T6 | 1-226 | LA 1-5 | 25 |
| LRP4-T6-1 | 1-183 | LA 1-4 | 20 |
| LRP4-T6-2 | 1-144 | LA 1-3 | 16 |
| LRP4-T6-3 | 1-106 | LA 1-2 | 11 |

**Table S2. Sequence of LA5 domain of wild-type and mutated LRP4**

**(Note:** sequences of LA5 domain within wild-type and mutated LRP4 were shown, the rest residues of LRP4 remained unchanged.)

| **Muteins ID** | **Sequence of LA5 domain within LRP4** |
| --- | --- |
| WT LRP4 | PCNLEEFQCAYGRCILDIYHCDGDDDCGDWSDESDCS |
| LRP4-m1 | AAALEEFQCAYGRCILDIYHCDGDDDCGDWSDESDCS |
| LRP4-m2 | PCNAAAFQCAYGRCILDIYHCDGDDDCGDWSDESDCS |
| LRP4-m3 | PCNLEEAAAAYGRCILDIYHCDGDDDCGDWSDESDCS |
| LRP4-m4 | PCNLEEFQCAAARCILDIYHCDGDDDCGDWSDESDCS |
| LRP4-m5 | PCNLEEFQCAYGAAALDIYHCDGDDDCGDWSDESDCS |
| LRP4-m6 | PCNLEEFQCAYGRCIAAAYHCDGDDDCGDWSDESDCS |
| LRP4-m7 | PCNLEEFQCAYGRCILDIAAADGDDDCGDWSDESDCS |
| LRP4-m8 | PCNLEEFQCAYGRCILDIYHCAAADDCGDWSDESDCS |
| LRP4-m9 | PCNLEEFQCAYGRCILDIYHCDGDAAAGDWSDESDCS |
| LRP4-m10 | PCNLEEFQCAYGRCILDIYHCDGDDDCAAASDESDCS |
| LRP4-m11 | PCNLEEFQCAYGRCILDIYHCDGDDDCGDWAAASDCS |
| LRP4-m12 | PCNLEEFQCAYGRCILDIYHCDGDDDCGDWSDEAAAA |
| LRP4-m46 | PCNLEEFQCAAARCIAAAYHCDGDDDCGDWSDESDCS |
| LRP4-m47 | PCNLEEFQCAAARCILDIAAADGDDDCGDWSDESDCS |
| LRP4-m67 | PCNLEEFQCAYGRCIAAAAAADGDDDCGDWSDESDCS |
| LRP4-m467 | PCNLEEFQCAAARCIAAAAAADGDDDCGDWSDESDCS |
